# Hydrophobic Metal–Organic Frameworks: Assessment, Construction, and Diverse Applications

**DOI:** 10.1002/advs.201901758

**Published:** 2020-01-19

**Authors:** Lin‐Hua Xie, Ming‐Ming Xu, Xiao‐Min Liu, Min‐Jian Zhao, Jian‐Rong Li

**Affiliations:** ^1^ Beijing Key Laboratory for Green Catalysis and Separation Department of Chemistry and Chemical Engineering College of Environmental and Energy Engineering Beijing University of Technology Beijing 100124 P. R. China

**Keywords:** anticorrosion coating, competitive adsorption, hydrophobicity index, postsynthetic modifications, substrate‐selective catalysis

## Abstract

Tens of thousands of metal–organic frameworks (MOFs) have been developed in the past two decades, and only ≈100 of them have been demonstrated as porous and hydrophobic. These hydrophobic MOFs feature not only a rich structural variety, highly crystalline frameworks, and uniform micropores, but also a low affinity toward water and superior hydrolytic stability, which make them promising adsorbents for diverse applications, including humid CO_2_ capture, alcohol/water separation, pollutant removal from air or water, substrate‐selective catalysis, energy storage, anticorrosion, and self‐cleaning. Herein, the recent research advancements in hydrophobic MOFs are presented. The existing techniques for qualitatively or quantitatively assessing the hydrophobicity of MOFs are first introduced. The reported experimental methods for the preparation of hydrophobic MOFs are then categorized. The concept that hydrophobic MOFs normally synthesized from predesigned organic ligands can also be prepared by the postsynthetic modification of the internal pore surface and/or external crystal surface of hydrophilic or less hydrophobic MOFs is highlighted. Finally, an overview of the recent studies on hydrophobic MOFs for various applications is provided and suggests the high versatility of this unique class of materials for practical use as either adsorbents or nanomaterials.

## Graphical abstract

The structural design, preparation strategies, characterization methods, and potential applications of hydrophobic metal–organic frameworks (MOFs), a class of unique materials with both microporosity and hydrophobicity, are overviewed herein. It is highlighted that hydrophobic MOFs can be prepared by some facile procedures, and this type of materials can act as either advanced adsorbents or nanomaterials.

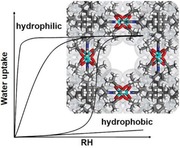

## 1 Introduction

Metal–organic frameworks (MOFs), also called porous coordination polymers (PCPs), are a class of porous materials commonly obtained by the facile hydrothermal or solvothermal reactions of metal ions and bridging organic ligands at relatively low temperatures.^1^, ^2^, ^3^, ^4^, ^5^, ^6^ In the past two decades, extensive research has been devoted to developing new MOFs^7^, ^8^, ^9^, ^10^, ^11^, ^12^, ^13^, ^14^, ^15^, ^16^, ^17^, ^18^, ^19^, ^20^, ^21^, ^22^, ^23^, ^24^, ^25^, ^26^, ^27^, ^28^, ^29^, ^30^ and to exploring their application potential in many fields, such as gas storage,^31^, ^32^, ^33^, ^34^, ^35^ separation,^36^, ^37^, ^38^, ^39^, ^40^, ^41^, ^42^, ^43^, ^44^, ^45^, ^46^, ^47^, ^48^ catalysis,^49^, ^50^, ^51^, ^52^ sensing,^53^, ^54^, ^55^, ^56^, ^57^, ^58^, ^59^, ^60^, ^61^ and biomedicine.^62^, ^63^, ^64^ Early on, a handful of MOFs was found to show a low affinity toward water and a permanent porosity, and this class of hydrophobic MOFs later received increasing attention due to their potential for use in practical adsorption and separation processes, even under humid conditions or in water. On the other hand, the stability of MOFs to water has long been regarded as the major weakness of MOFs in practical use. Many studies have demonstrated that the hydrolytic stability of MOFs could be enhanced by improving the hydrophobicity of their internal pore surface and/or external crystal surface.^65^, ^66^ Recently, a variety of synthetic approaches have emerged to make MOFs more hydrophobic. The integrated attributions of the reported hydrophobic MOFs with respect to porosity, hydrophobicity and stability lead to great promise for this unique class of materials in diverse applications, including humid CO_2_ capture, alcohol/water separation, pollutant removal from air or water, substrate‐selective catalysis, energy storage, anticorrosion coatings and self‐cleaning. Some excellent reviews about hydrophobic MOFs have been recently published.^67^, ^68^, ^69^, ^70^ However, the goal of this contribution is to present a comprehensive review of the development and advancement of hydrophobic MOFs.

In the following sections, the existing techniques for assessing the hydrophobicity of MOFs are first introduced, including the determination of crystal structure, water adsorption measurement, quantitative determination of the hydrophobicity index by the competitive breakthrough adsorption experiment of a hydrocarbon/water mixture, and water contact angle measurement. These measurements offer information about the extent of the hydrophobicity of MOFs at the internal pore surface or external crystal surface. Then, the reported methods to construct hydrophobic MOFs, which are categorized into three classes: presynthetic ligand design and functionalization, postsynthetic hydrophobization, and in situ synthetic hydrophobization, are summarized. A list of hydrophobic MOFs obtained from various predesigned ligands (carboxylic acids, azoles, and phosphonic acid monoesters) is provided. It is highlighted that hydrophobic MOFs normally synthesized from predesigned organic ligands can also be prepared by a postsynthetic modification of the internal pore surface and/or external crystal surface of hydrophilic or less hydrophobic MOFs. Last, the recent studies on hydrophobic MOFs for various applications are overviewed. The interplay of porosity, hydrophobicity, stability, and application performance of the MOF materials is discussed.

## 2 Assessment of Hydrophobicity in MOFs

**Figure 1:**
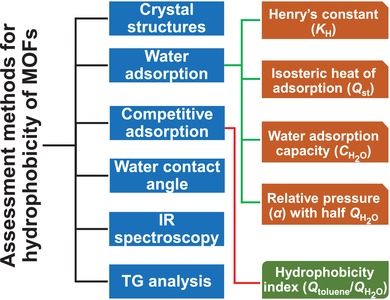
Methods and indicators for assessing the hydrophobicity of MOFs.

Among the over 20 000 MOFs reported so far,^6^ ≈100 MOFs have been described as hydrophobic. Although many of these MOFs were reported without extra experimental evidence except crystal structures supporting their hydrophobicity, some experimental analysis methods are often used to assess the hydrophobicity of MOFs qualitatively or quantitatively (**Figure** 1), namely, the water adsorption isotherm measurement, competitive adsorption of a vapor mixture of water and hydrocarbon vapor (mostly toluene) in a breakthrough experiment, and contact angle of liquid water. These methods are discussed in detail in the following sections. Occasionally, infrared (IR) spectroscopy and thermogravimetric (TG) analysis are also used for accessing the hydrophobicity of MOFs.^71^, ^72^, ^73^ IR spectroscopy can be used to determine the water content in the hydrophobic pore by monitoring absorption near 3680 cm^−1^ for the O–H stretching bands of water and absorption near 1621 cm^−1^ for the H–O–H bending bands of water. Due to hydrogen bonding interactions, O–H stretching bands are commonly observed near 3400 cm^−1^ as well. TG curves not only give the quantities of water adsorbed by the porous materials but also provide clues to evaluate the strength of the adsorbent–water interactions, which are indicated by the temperature range of the weight loss for adsorbed water molecules.

### 2.1 Crystal Structures

The studies of MOFs start with the crystal structure. Most MOFs were structurally determined by single‐crystal X‐ray diffraction (SXRD) experiments. With the SXRD crystal structures, the pore surface of MOFs can be examined, leading to a general judgment of the hydrophobicity/hydrophilicity of their internal surfaces. When the pore surface is mostly covered by nonpolar or weakly polar contents that have low surface energies, such as alkyl groups, aryl groups, fluorinated alkyl and aryl groups, the pore surface hydrophobicity is clearly indicated. On the other hand, the presence of moieties prone to coordinate or hydrogen bond with water molecules (such as open metal sites, as well as hydroxyl, amine, and sulfonic acid groups) on the pore surface generates a high surface energy and thus enhances the hydrophilicity of the inner surface. Indeed, the crystal structures of MOFs provide information to predict their hydrophobicity, especially when the guest‐free crystal structures of MOFs are unambiguously determined. However, sometimes claims that MOFs were described to be hydrophobic based on only their crystal structures, without other supporting experimental results, are not convincing. In addition, it should be noted that the crystal structures of MOFs cannot be used to predict the hydrophobicity/hydrophilicity of their external surfaces, because the external surface is a kind of crystal defect, and the bonding environment of the atoms on the surface can be very different from those inside the bulk.

### 2.2 Water Adsorption

**Figure 2:**
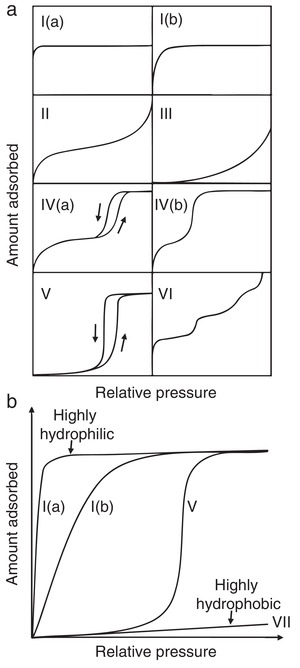
a) Five types of physisorption isotherms as classified by IUPAC. b) Representative water adsorption isotherms of porous adsorbents with different degrees of hydrophobicity/hydrophilicity.

The adsorption isotherms of water vapor recorded near room temperature for MOFs offer important information about the hydrophobicity/hydrophilicity of their internal surfaces.^39^, ^65^, ^74^ There are six types of physisorption isotherms in the IUPAC recommendations updated in 2015 (**Figure** 2a).^75^ Hydrophobic microporous and mesoporous adsorbents commonly show type V isotherms, where the uptakes are low at the low‐relative‐pressure (*P*/*P*_0_) range, indicating the presence of relatively weak adsorbent–adsorbate interactions, and molecular clustering is followed by pore filling at the high‐*P*/*P*_0_ range. For the water adsorption isotherms of some highly hydrophobic MOFs, such as [Zn(2‐mim)_2_] (ZIF‐8),^76^, ^77^ Zn(2‐eim)_2_ (MAF‐6),^78^ Zn(4,5‐dcim)_2_ (ZIF‐71),^79^ and {Ag_2_[Ag_4_(3,5‐tftz)_6_]} (FMOF‐1),^80^ the uptakes are very low, even at the pressures near the saturation pressure (*P*_0_), as with the type VII adsorption isotherm shown in Figure 2b.^81^ As the hydrophilicity of MOFs increases, their water adsorption isotherms gradually transform into type I isotherms (Figure 2b).

Many MOFs have been characterized by their water adsorption isotherms, and some excellent reviews discussing the water adsorption isotherms of MOFs have also been reported.^39^, ^65^, ^74^, ^82^ From the water adsorption isotherms, several indicators for accessing the hydrophobicity of MOFs can be obtained, including Henry's constant (*K*_H_), the heat of water adsorption (*Q*_st_), the water adsorption capacity ($C_{{\text{H}}_{2}\text{O}}$), and the relative pressure (α) at which half of the water adsorption capacity is reached. *K*_H_ is the slope of a water isotherm at the low‐pressure range (the so‐called Henry range), where the uptake is linearly dependent on the *P*/*P*_0_ value. The quantity of *K*_H_ is an indication of the interaction strength between water molecules and the MOF framework. *Q*_st_ can be obtained from determining the heat released during a water adsorption process by an isothermal calorimeter^83^ or calculated from several adsorption isotherms recorded at close temperatures (≈10 K differences) with the Clausius–Clapeyron equation (Equation (1)),^84^, ^85^ where *T* is the temperature, *R* is the universal gas constant, and *C* is a constant. *Q*_st_ also indicates how strong the adsorbent–adsorbate interaction strength is. $C_{{\text{H}}_{2}\text{O}}$, standing for the water uptakes at the pressures close to *P*_0_, are dependent on not only the adsorbent–water affinity but also the pore volume of the adsorbent. Less hydrophobic adsorbents with larger pore volumes have higher $C_{{\text{H}}_{2}\text{O}}$. Normally, the pore volumes of nanoporous adsorbents can be precisely determined by N_2_ adsorption at 77 K or by Ar adsorption at 87 K. Especially, in some cases, when the SXRD structures of the guest‐free phases of MOFs are determined, the experimental pore volumes obtained from N_2_ or Ar adsorption can be very close to the theoretical pore volumes predicted from the guest‐free SXRD structures. By assuming that the pores are occupied by water in a liquid or quasi‐liquid status at a pressure near *P*_0_, a theoretical water adsorption capacity ($C_{{\text{H}}_{2}\text{O}}^{\text{T}}$) can be calculated by Equation (2), where ρ stands for the density of water confined in a pore and *V* is the pore volume determined experimentally. In the literature, either the density of liquid water (1 g cm^−3^)^86^, ^87^ or the density of crystallized water (ice) (0.9168 g cm^−3^)^88^ was adapted as ρ in the calculation. A comparison of the calculated $C_{{\text{H}}_{2}\text{O}}^{\text{T}}$ and $C_{{\text{H}}_{2}\text{O}}$ determined from water adsorption experiments offers information about how hydrophobic the adsorbent is. A large difference between $C_{{\text{H}}_{2}\text{O}}^{\text{T}}$ and $C_{{\text{H}}_{2}\text{O}}$ indicates a high hydrophobicity of the adsorbent. Several hydrophobic nanoporous adsorbents show type V water adsorption isotherms. Due to pore filling at relatively high *P*/*P*_0_, $C_{{\text{H}}_{2}\text{O}}$ at pressures near *P*_0_ may be very close to the maximum water uptake $C_{{\text{H}}_{2}\text{O}}^{\text{T}}$. In such cases, α, the relative pressure at which half of $C_{{\text{H}}_{2}\text{O}}$ is reached, is an important complementary indicator for assessing the hydrophobicity of adsorbents. A high α is an indication of a high hydrophobicity

(1) $${\left(\text{In}\;P\right)}_{N}=-\frac{Q_{\text{st}}}{R}\frac{1}{T}+C$$

(2) $$C_{{\text{H}}_{2}\text{O}}^{\text{T}}=\rho \times V$$

(3) $$\text{HI}=\frac{Q_{\text{toluene}}}{Q_{\text{water}}}$$

### 2.3 Competitive Breakthrough Adsorption

Although water adsorption provides abundant important information, assessing the hydrophobicity of the internal surfaces of adsorbents with water adsorption isotherms and the derived indicators is qualitative. Directly comparing the hydrophobicity of different adsorbents by their water adsorption isotherms can sometimes be misleading because their pore sizes, shapes, and volumes are different, affecting their water adsorption isotherm significantly. In contrast, the hydrophobicity index (HI)—defined by the ratio of the adsorption capacity of toluene (*Q*_toluene_) to that of water (*Q*_water_), which can be determined by the competitive breakthrough adsorption experiment with a toluene/water mixture (Equation (3))—is a promising indicator for quantitatively assessing the hydrophobicity of MOFs. However, the HIs of only two MOFs have been reported so far, namely, [Fe^III^_3_O(H_2_O)_2_F(btc)_2_] (MIL‐100(Fe)) (HI = 2.8)^89^ and [Cr_3_O(H_2_O)_2_F(1,4‐bdc)_3_] (MIL‐101(Cr)) (HI = 11.0).^90^ For comparison, the reported HIs of some zeolites and activated carbons are listed in **Table** 1. The HI of MIL‐100(Fe) is only slightly higher than that of typical hydrophilic zeolites, such as Zeolites beta (1.4) and Zeolites Y (0), but is clearly lower than that of all‐silica zeolites and activated carbons, which are commonly regarded as hydrophobic adsorbents, thus suggesting the relatively high hydrophilicity of MIL‐100(Fe). The HI of MIL‐101(Cr) is higher than those of hydrophilic zeolites, but lower than those of typical hydrophobic adsorbents, suggesting that MIL‐101(Cr) is in an intermediate status between hydrophilic and hydrophobic. This identification explains why MIL‐101(Cr) was described as hydrophobic in some papers,^91^, ^92^ but hydrophilic in others.^90^, ^93^

**Table 1:** Hydrophobicity indexes of some adsorbents obtained from the co‐adsorption of toluene and water

Adsorbent	Hydrophobicity index (HI)	Reference
MIL‐100(Fe)	2.8	89
MIL‐101(Cr)	11.0	90
Zeolites beta	1.4	94
Zeolites beta, all‐silica	66	94
Zeolites Y	0	94
ZSM‐5	8	95
Silicalite‐1	15.2	96
MCM‐41	9	94
Activated carbon (Darco‐KBB)	26.3	97
Activated carbon (SX1G)	26.2	97
Activated carbon (F300)	160	94
Activated carbon (Duksan)	296	90

It should be noted that other methods exist to define the HI of porous adsorbents.^81^ Fox example, the HI can also be determined by the competitive adsorption of mixtures of water and hydrocarbons other than toluene, including methylcyclohexane.^96^ In addition, Anderson and Klinowski introduced an HI for zeolites, which is defined as the ratio of the loss of water at 150 °C to that at 400 °C obtained by thermogravimetric analysis (TGA).^98^ In any case, when assessing the hydrophilicity/hydrophilicity by comparing HIs, the same method for determination of the HIs should be adopted.

### 2.4 Water Contact Angle

**Figure 3:**
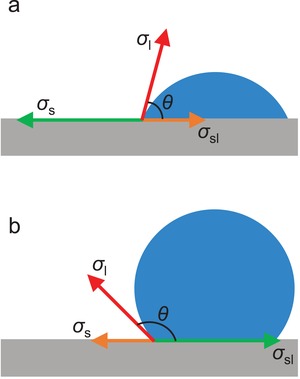
Water contact angles for a) a hydrophilic solid surface and b) a hydrophobic solid surface.

The hydrophobicity of the external surface of MOFs is commonly evaluated by the measurement of the water contact angle of their crystal, powder or/and compressed pellet samples. Water contact angles less than 90° suggest a favorable wetting of the external surfaces of MOFs by water, which are regarded to be hydrophilic (**Figure** 3a). Water contact angles greater than 90° indicate an unfavorable wetting and a hydrophobic external surface of MOFs. The external surface of MOFs is commonly described to be superhydrophobic when it shows water contact angles greater than 150° (Figure 3b).

The contact angle of a liquid drop on an ideal solid surface is defined by Young's equation (Equation (4)), where σ_l_ is the surface tension of the liquid, θ is the contact angle, σ_s_ is the surface free energy of the solid, and σ_sl_ is the interfacial tension between the liquid and solid. Interfacial tension is defined as the work that is required to increase the area of the interface between two adjacent phases, which do not mix completely with one another, in the unit of mJ m^−2^ (also frequently used as mN m^−1^). The liquid/gas interfacial tension is also termed as surface tension, while the solid/gas interfacial tension is also termed as the surface free energy. Therefore, σ_l_ and σ_s_ in Young's equation are sometimes described as the liquid–gas interfacial tension (σ_lg_) and solid–gas interfacial tension (σ_sg_), respectively.^99^ When the liquid is water, the surface tension of water, σ_l_, is a constant at a specific temperature, thus, the water contact angle (θ) is dependent on the surface free energy of the solid surface σ_sl_ and the interfacial tension between the solid surface and the liquid water, σ_sl_. When the surface free energy of the solid is high and dominative (θ < 90°), the small water drop expands and covers more solid surface until the water contact angle reduces to a value by which the Young's equation is balanced (Figure 3a). When the affinity between the solid surface and water is low (low wettability), the solid–water interfacial tension is high and dominative (θ > 90°), and the water drop prefers not to increase the area of the solid–water interface (Figure 3b)

(4) $$\sigma_{\text{l}}\times \cos\theta =\sigma_{\text{s}}-\sigma_{\text{sl}}$$

**Figure 4:**
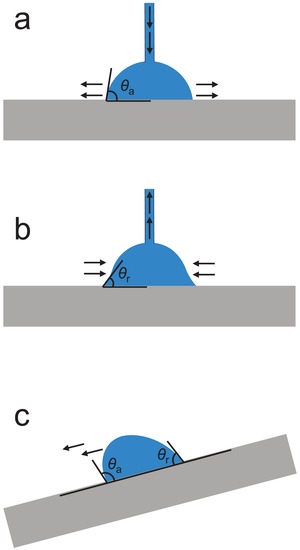
Measurement of dynamic contact angles a,b) by the volume changing method and c) by the tilted plate method.

In Young's equation, the solid surface is assumed to be ideally homogeneous, smooth and inert. However, the surfaces of MOF samples (crystals, powder, and pellets) for the water contact angle measurement are normally heterogeneous and rough. To study how the real surfaces differ from the ideal surface, the dynamic contact angle measurement is commonly performed. Dynamic contact angles can be measured by slowly introducing or removing water from a water drop on the solid surface (the so‐called volume changing method). When water is added slowly enough, the water contact angle increases as the three‐phase boundary remains unchanged. The maximum contact angle obtained before three‐phase boundary starts to expand is termed as the advancing contact angle, θ_a_ (**Figure** 4a). When water is removed from the water drop very slowly, the water contact angle gradually decreases. The minimum water contact angle recorded at the moment the three‐phase boundary starts to contract is called the receding contact angle, θ_r_ (Figure 4b). Contact angle hysteresis is defined as the difference between θ_a_ and θ_r_. Dynamic contact angles are also often recorded by the so‐called tilted plate method. In this measurement, the water drop on a solid surface gradually deforms as the solid surface is tilted slowly enough to keep the water drop stationary. In addition, the contact angle of the downhill side of the water drop gradually increases, and that of the uphill side of the water drop gradually decreases. When the surface is tilted to a specific angle, a maximum contact angle at the downhill side and a minimum contact angle at the uphill side are reached, and the water drop starts to move. The obtained maximum and minimum contact angles are θ_a_ and θ_r_, respectively (Figure 4c).

## 3 Preparation of Hydrophobic MOFs

The reported hydrophobic MOFs are mostly synthesized by ligands with attached hydrophobic groups. In the past few years, some excellent works have demonstrated that hydrophobic MOFs or MOF‐derived materials can also be obtained by the postsynthetic or in situ synthetic modification of less hydrophobic or hydrophilic MOFs. Some representative reported hydrophobic MOFs are listed in **Table** 2 together with their pore characteristics and hydrophobicities.

**Table 2:** A list of representative hydrophobic MOFs reported in the literature

MOF	Formula	Ligand^a)^	*S*_BET_/*S*_L_ ^b)^ [m^2^ g^−1^]	*V*_p_ ^c)^ [cm^3^ g^−1^]	*d*_a_ ^d)^ [Å]	*d*_p_ ^e)^ [Å]	$C_{{\text{H}}_{2}\text{O}}$ ^f)^ [mg g^−1^]	α^g)^ (*P*/*P*_0_)	CA^h)^ [°]	Ref.
[Cd(O_2_C‐CH_2_‐CO_2_)(H_2_O)]	[Cd(ma)(H_2_O)]	H_2_ma			6.95					100
[Mn(O_2_C‐(CH_2_)_3_‐CO_2_)]	[Mn(glu)]	H_2_glu			6.6					100
CPM‐300	[Zn_4_O(RR‐cam)_3_]	RR‐H_2_cam	310.5/ 429.9		8				138.7	101
CPM‐301	[Zn_4_O(RRcam)_3_][Zn_9_(btz)_12_(RR‐cam)_3_]	RR‐H_2_cam, Hbtz							145.0	101
MIL‐140A	[ZrO(1,4‐bdc)]	1,4‐H_2_bdc	415/–	0.18	3.2		–^i)^	0.5		71
MIL‐101(Cr)	[Cr_3_O(H_2_O)_2_F(1,4‐bdc)_3_]	1,4‐H_2_bdc	3124/–	1.58	12, 16 × 14.5	29, 34	1360	0.48		102
MIL‐101‐NO_2_	[Cr_3_O(H_2_O)_2_F(1,4‐bdc‐2‐NO_2_)_3_]	1,4‐H_2_bdc‐2‐NO_2_	2146/–	1.19			1060	0.47		102
MIL‐68(In)	[In(OH)(1,4‐bdc)]	1,4‐H_2_bdc	1100/–	0.42	6.0–6.4; 16–17		320	0.58		103
Banasorb‐22	[(Zn_4_O)(1,4‐bdc‐2‐CF_3_O)_3_]	1,4‐H_2_bdc‐2‐CF_3_O	1113/–				80			72
UiO‐66	[Zr_6_O_4_(OH)_4_(1,4‐bdc)_6_]	1,4‐H_2_bdc	1105/–	0.55	7	8–11	453	0.27		104
UiO‐66−1,4‐Naphyl	[Zr_6_O_4_(OH)_4_(1,4‐ndc)_6_]	1,4‐H_2_ndc	757/–	0.42			297	0.31		104
UiO‐66‐(C_2_H_5_)_2_	[Zr_6_O_4_(OH)_4_(1,4‐bdc‐(C_2_H_5_)_2_)_6_]	1,4‐H_2_bdc‐(C_2_H_5_)_2_	340/–	0.16			122	0.5		87
UiO‐66‐C_2_F_5_	[Zr_6_O_4_(OH)_4_(1,4‐bdc‐C_2_F_5_)_6_]	1,4‐H_2_bdc‐C_2_F_5_	570/–	0.26			252	0.6		87
UiO‐66‐(CF_3_)_2_	[Zr_6_O_4_(OH)_4_(1,4‐bdc‐(CF_3_)_2_)_6_]	1,4‐H_2_bdc‐(CF_3_)_2_	630/–	0.30			190	0.35		87
UiO‐66‐CH_3_	[Zr_6_O_4_(OH)_4_(1,4‐bdc‐CH_3_)_6_]	1,4‐H_2_bdc‐CH_3_	760/–	0.32			253	0.22		87
UiO‐66‐(CH_3_)_2_	[Zr_6_O_4_(OH)_4_(1,4‐bdc‐(CH_3_)_2_)_6_]	1,4‐H_2_bdc‐(CH_3_)_2_	790/–	0.35			279	0.38		87
UiO‐66‐CF_3_	[Zr_6_O_4_(OH)_4_(1,4‐bdc‐CF_3_)_6_]	1,4‐H_2_bdc‐CF_3_	815/–	0.36			279	0.32		87
MIL‐140B	[ZrO(2,6‐ndc)]	2,6‐H_2_ndc	460/–	0.18	4.0		%	0.5		71
MIL(Cr)‐Z1	[Cr_3_O(H_2_O)_2_F(1,4‐ndc)_3_]	1,4‐H_2_ndc	2086/–	1.23	6.9	23.3	318.6	0.64		105
Al(OH)(1,4‐ndc)	[Al(OH)(1,4‐ndc)]	1,4‐H_2_ndc	–/546		3.0–7.7		165	0.47		106
[Cu(1,4‐ndc)(MeOH)]	[Cu(1,4‐ndc)(MeOH)]	1,4‐H_2_ndc	–/133.7		≈3.8		22	0.4		107
UiO‐67‐BN	[Zr_6_O_4_(OH)_4_(bn)_6_]	H_2_bn	1416/–				495	0.54		108
UiO‐67‐4MS	[Zr_6_O_4_(OH)_4_(tmbpd)_6_]	H_2_tmbpd	1153/–					82		109
UiO‐66D‐(CF_3_)_2_	[Zr_6_O_4_(OH)_4_(abdc‐(CF_3_)_2_)_6_]	H_2_abdc‐(CF_3_)_2_	2180/–	0.88			281	0.87		87
NMOF‐1	[Zn(ope‐C_18_)(H_2_O)_2_]	H_2_ope‐C_18_			≈3.3		16	≈0.45	160–162	110
IFMC‐29	[Zn_4_O(CH_3_PhTDC)_3_]	H_2_CH_3_PhTDC	1892/–	0.64					93	111
MOFF‐1	[Cu(bpdc‐F_8_)(MeOH)]	H_2_bpdc‐F_8_	580/–				136	0.45	108	112
Zn(tbip)	[Zn(tbip)]	H_2_tbip	256/–	0.15	4.5		110	0.56		113
Zn‐hfipbb	[Zn(hfipbb)]	H_2_hfipbb	287/288.9	0.112	6.7		≈0			114, 115
[Cu(hfipbb)(H_2_hfipbb)_0.5_]	[Cu(hfipbb)(H_2_hfipbb)_0.5_]	H_2_hfipbb		0.07	3.5	5.1				116, 117
Fe‐hfipbb	[Fe_3_O(hfipbb)_3_]	H_2_hfipbb	269.5/–	0.21	8–12.9				110	118
MAMS‐2	[Zn(H_2_O)(bbpdc)]	H_2_bbpdc			2.9–4.6					119
UHMOF‐100	[Cu(tpda)(DMF)]	H_2_tpda	660/469	1.07	5.9		≈0		177	120
BUT‐66	[Zr_6_O_4_(OH)_4_(1,3‐bdb)_6_]	1,3‐H_2_bdb	1096/1291	0.46	6.0		71	0.4	142.8	88
BUT‐67	[Zr_6_O_4_(OH)_4_(2,7‐ndb)_6_]	2,7‐H_2_ndb	984/1141	0.41	7		124	0.45	137.9	88
CuMBTC	[Cu_3_(mbtc)_2_]	H_3_mbtc	1471/–	0.78	8.8–12.7		184	0.2		121
CuEBTC	[Cu_3_(ebtc)_2_]	H_3_ebtc	1434/–	0.65	7.8–12.2		184	0.1		121
BUT‐12	[Zr_6_O_4_(OH)_8_(H_2_O)_4_(ctta)_8/3_]	H_3_ctta	3387/–	1.52	13–21		434	0.4	138.7	122
BUT‐13	[Zr_6_O_4_(OH)_8_(H_2_O)_4_(ttna)_8/3_]	H_3_ttna	3948/–	2.20	14–28		494	0.52	118.3	122
PESD‐1	[Zn_4_(OH)_2_(btmb)_2_(DMF)_3_(MeOH)]	H_3_btmb	295/570						>150	123
[Pb(H‐BTMB)]	[Pb(Hbtmb)(DMF)]	H_3_btmb	155/–		≈3.3				156.4	124
Cu_2_(TPTC‐O*^n^*Hex)	[Cu_2_(TPTC‐O*^n^*Hex)]	H_4_tptc‐o*^n^*hex	1083/1269							125
UPC‐21	[Cu_2_(pptc)]	H_4_pptc	1253.6/–						145	126, 127
BUT‐155	[Cu_4_(tdhb)]	H_8_tdhb	2070/–	0.82	9.5	16	470	0.28		128
Znpbdc‐8a	–	pbdc‐8a	856/–		9				112	129
FMOF‐1	{Ag_2_[Ag_4_(3,5‐tftz)_6_]}	3,5‐Htftz	810.5/–	0.324	12. × 8	6.6 × 4.9			158	80, 130, 131
FMOF‐2	[Ag(Ag_3_(3,5‐tftz)_4_)]	3,5‐Htftz			18	10				80
MAF‐4/ZIF‐8	[Zn(2‐mim)_2_]	2‐Hmim	–/1870	0.67	3.2	11.4	26	0.88		77
ZIF‐318	[Zn(2‐mim)(mim‐F_3_)]	2‐Hmim, 2‐Hmim‐F_3_	835/1007	0.37	9.3		23	0.58	68.4	132
ZIF‐68	[Zn(bim)(2‐nim)]	Hbim, 2‐Hnim			7.5	10.3	30^j)^	0.52		133, 134
ZIF‐71	[Zn(4,5‐dcim)_2_]	4,5‐Hdcim	1183/1350	0.385	4.2	16.5	4.5	0.3		79, 133, 135, 136
ZIF‐90	[Zn(2‐cim)_2_]	2‐Hcim	1280/1466	0.485	3.5	11.2	331	0.35		79, 137
ZIF‐300	[Zn(2‐mim)_0.86_(5‐bbim)_1.14_]	5‐Hbbim, 2‐Hmim	420/490				6	0.58		138
ZIF‐301	[Zn(2‐mim)_0.94_(5‐cbim)_1.06_]	5‐Hcbim, 2‐Hmim	680/825				5.8	0.52		138
ZIF‐302	[Zn(2‐mim)_0.67_(5‐mbim)_1.33_]	5‐Hmbim, 2‐Hmim	240/270				4.5	0.53		138
MAF‐5	[Zn(2‐eim)_2_]	2‐Heim			2.2–4.9	7–10	≈0			139
MAF‐6	[Zn(2‐eim)_2_]	2‐Heim	1343/1695	0.61	7.6	18.4	16.2	≈0.5	143	78
[Ni_8_(L_3_)_6_]	[Ni_8_(OH)_4_(H_2_O)_2_(1,4‐bdp)_6_]	1,4‐H_2_bdp	1770/–				990	0.65		140
[Ni_8_(L_4_)_6_]	[Ni_8_(OH)_4_(H_2_O)_2_(bpdy)_6_]	H_2_bpdy	1920/–				936	0.5		140
[Ni_8_(L_5_)_6_]	[Ni_8_(OH)_4_(H_2_O)_2_(bpeb)_6_]	H_2_bpeb	2215/–		≈24		1129	0.7		140
[Ni_8_(L_5_‐CH_3_)_6_]	[Ni_8_(OH)_4_(H_2_O)_2_(bpdm)_6_]	H_2_bpdm	1985/–		≈24		720	0.7		140
[Ni_8_(L_5_‐CF_3_)_6_]	[Ni_8_(OH)_4_(H_2_O)_2_(bpdf)_6_]	H_2_bpdf	2195/–		≈24		862	0.83		140
Cu‐tebpz	[Cu_2_(tebpz)]	H_2_tebpz	576/–	0.37	4.6–13		6	0.5		141
Zn(NDI–SEt)	[Zn(ndi–SEt)]	H_2_ndi–SEt	888/–				237	0.4		142
MOFF‐3	[Cu(bptz‐F_8_)(H_2_O)]	H_2_bptz‐F_8_					37	0.3	135	112
MAF‐2	[Cu(3,5‐etz)]	3,5‐Hetz			1.5–4.2	9	7.4	0.5		143
MAF‐7	[Zn(3‐mtz)_2_]	3‐Hmtz	–/1870	0.67	3.4	11.2	443	0.33		77
MAF‐X5	[Zn(3,5‐dmtz)F]	3,5‐Hdmtz, F^−^	155/195		3.6					144
MAF‐52	[Cu_7_Cl(fmtz)_6_]	Hfmtz	1023/848	0.365	7.2 × 8.2, and 7.4 × 8.3	10.6	0.81		148	145
SCUTC‐18	[Zn_2_(1,4‐bdc)_2_(2,2′‐dmbpy)]	1,4‐H_2_bdc, 2,2′‐dmbpy	523/–		8.0					146
SCUTC‐19	[Zn_2_(1,4‐bdc)_2_(3,3′‐dm‐bpy)]	1,4‐H_2_bdc, 3,3′‐dm‐bpy	458/–		6.5					146
*o*CB‐MOF‐1,	[Zn_4_O_2_(1,4‐bdc)2(oCB‐L)_2_(DMF)_2_]	1,4‐H_2_bdc, oCB‐L	296/–		3.2 × 6.4	8.6	50	0.5	140	147
CID‐1	[Zn_2_(ip)_2_(4,4′‐bpy)_2_]	Hip, 4,4′‐bpy	300/–		5 × 6		64^k)^	0.61		148
[Cu(bpbtp)(L)(DMF)]	[Cu(bpbtp)(dbbpy‐F_18_)(DMF)]	H_2_bpbtp. dbbpy‐F_18_								149
[Cd(NO_2_‐bdc)(azbpy)]	[Cd(1,4‐NO_2_‐bdc)(azbpy)]	1,4‐H_2_bdc‐2‐NO_2_, 4,4′‐azbpy					149	0.32		150
SNU‐80	[Cu_4_(pa)_8_(teia)]	Hpa, teia	1035/1167	0.43			10	0.5		151
[Cu(bpy)_2_(otf)_2_]‐3D	[Cu(4,4′‐bpy)_2_(otf)_2_]	4,4′‐bpy, Hotf	740/–	0.27						152
DMOF‐TM2	[Zn_2_(1,4‐bdc‐(CH_3_)_4_)_2_(dabco)]	1,4‐H_2_bdc‐(CH_3_)_4_, dabco	1050	0.51	3.5		412	0.26		153
MOFF‐2	[Cu_2_(bpdc‐F_8_)_2_(dabco)]	H_2_bpdc‐F_8_, dabco	444/–				11	0.53	151	112
DUT‐30(Zn)	[Zn_2_(adb)_2_(dabco)]	H_2_adb, dabco	960/–	0.43			366	0.95		154
Zn‐dmpc	[Zn_4_O(dmpc)_3_]	H_2_dmpc	840/–	0.45	4 × 4	6	437	0.83		155
MAF‐X8	[Zn(mpba)]	H_2_mpba	1161/1306	0.47	6.7–8.8					156
MAF‐X10	[Zn_4_O(tmbpz)_2_(1,4‐bdc)]	1,4‐H_2_bdc, H_2_tmbpz	2032/–	0.80	6.6 × 5.8	9.4 × 9.9 × 13.2				157, 158
MAF‐X12	[Zn_4_O(tmbpz)_2_(1,4‐ndc)]	1,4‐H_2_ndc, H_2_tmbpz	1787/–	0.71	3.0 × 5.8	9.4 × 9.9 × 13.2				157
MAF‐X13	[Zn_4_O(tmbpz)_2_(bpdc)]	H_2_bpdc, H_2_tmbpz	2742/–	1.01	6.6 × 10.0	9.4 × 9.9 × 15.9				157
CALF‐25	–	optp	385/–		4.6 × 3.9		76.9	0.73		85
CALF‐30	[Cu_3_(btp‐^i^Pr)_2_]	H_3_btp‐^i^Pr	244/300		3.57					159
CALF‐33‐Et_3_	[Cu_3_(L1‐Et_3_)_2_]	H_3_L1‐Et_3_	842/1030		7.2 × 16.1					160

^a)^ 1,4‐H_2_bdc‐2‐NO_2_, 2‐nitro terephthalic acid; 1,4‐H_2_bdc‐(C_2_H_5_)_2_, 2,5‐diethylterephthalic acid; 1,4‐H_2_bdc‐C_2_F_5_, 2‐pentafluoroethylterephthalic acid; H_2_ma, propanedioic acid; 1,4‐H_2_bdc‐(CF_3_)_2_, 2,5‐bis(trifluoromethyl)terephthalic acid; 1,4‐H_2_bdc‐CH_3_, 2‐methyl‐terephthalic acid; 1,4‐H_2_bdc‐(CH_3_)_2_, 1,4‐H_2_bdc‐(CH_3_)_4_, 2,3,5,6‐tetramethylterephthalic acid; 2,5‐dimethyl‐terephthalic acid; 1,4‐H_2_bdc‐CF_3_, 2‐trifluoromethyl‐terephthalic acid; 1,4‐H_2_bdc‐F_4_, 2,3,5,6‐tetrafluoroterephthalic acid; 2,6‐H_2_ndc, 2,6‐naphthalenedicarboxylic acid; H_2_bn, 1,1′‐binaphthyl‐4,4′‐dicarboxylic acid; 3‐Hmtz, 3‐methyl‐1,2,4‐triazole; 2‐Hmim, 2‐methylimidazole; 2‐Hmim‐F_3_, 2‐trifluoromethylimidazole; 2‐Hvim, 2‐vinyl‐imidazole; 2‐Hmimc, 4‐methyl‐5‐imidazolecarboxaldehyde; H_2_hfipbb, 4,4′‐(hexafluoroisopropylidene)bis(benzoic acid); 1,4‐H_2_bdp, 1,4‐bis(4‐pyrazolyl)benzene; H_2_bpdy, 4,4′‐buta‐1,3‐diyne‐1,4‐diylbis(1*H*‐pyrazole); H_2_bpeb, 1,4‐bis((1*H*‐pyrazol‐4‐yl)ethynyl)benzene; H_2_bpdm, 4,4′‐(2,5‐dimethyl‐1,4‐phenylene)bis(ethyne‐2,1‐diyl)bis(1*H*‐pyrazole); H_2_bpdf, 4,4′‐(2,5‐bis(trifluoromethyl)‐1,4‐phenylene)bis(ethyne‐2,1‐diyl)bis(1*H*‐pyrazole); H_3_btmb, 1,3,5‐tris(3‐carboxyphenyl)benzene; pbdc, polymeric bdc‐acid; oCB‐L, 1,2‐bis{(pyridin‐3‐yl)methanol}‐1,2‐dicarba‐closo‐dodecarborane, Hip, isophthalic acid; 4,4′‐bpy, 4,4′‐bipyridine; 2,2′‐dm‐bpy, 2,2′‐dimethyl‐4,4′‐bipyridine; 3,3′‐dm‐bpy, 3,3′‐dimethyl‐4,4′‐bipyridine; H_2_bpdc‐F_8_, 2,2′,3,3′,5,5′,6,6′‐octafluorobiphenyl‐4,4′‐dicarboxylic acid; H_2_bptz‐F_8_, 5,5′‐(perfluorobiphenyl‐4,4′‐diyl)bis(1*H*‐tetrazole); dabco, 1,4‐diazabicyclo[2.2.2]octane; H_2_bpbtp, 2,5‐bis(perfluorobutyl)terephthalic acid, dbbpy‐F_18_, 2,5‐bis(perfluorobutyl)‐1,4‐bis(4‐pyridyl)benzene; 4,4′‐azbpy, 4,4′‐azobipyridine; H_3_btc, 1,3,5‐benzenetricarboxylic acid; H_3_mbtc, methyl‐1,3,5‐benzenetricarboxylic acid; H_3_ebtc, ethyl‐1,3,5‐benzenetricarboxylic acid; H_3_NH_2_btc, 2‐aminobenzene‐1,3,5‐tricarboxylic acid; H_4_tptc‐o*^n^*hex, 2′,5′‐di‐n‐hexyloxy‐[1,1′:4′,1′‐terphenyl]‐3,3″,5,5″‐tetracarboxylic acid; teia, 1,3,5,7‐tetrakis(4‐(2‐ethyl‐1*H*‐imidazol‐1‐yl)phenyl)‐adamantane; Hpa, pivalic acid; H_2_tpda, 4,4′‐(3,5‐bis(trifluoromethyl)phenylazanediyl)dibenzoic acid; H_2_ope‐C_18_, 4,4′‐(2,5‐bis(octadecyloxy)‐1,4‐phenylene)bis(ethyne‐2,1‐diyl)dibenzoic acid; H_2_mpba, 4‐(3,5‐dimethylpyrazol‐4‐yl)benzoic acid; H_2_glu, glutaric acid; H_2_abdc‐(CF_3_)_2_, 2,2′‐bistrifluoromethyl‐4,4′‐azobenzenedicarboxylic acid; H_2_tmbpz, 3,3′,5,5′‐tetramethyl‐4,4′‐bipyrazole; H_2_bpdc, biphenyl‐4,4′‐dicarboxylic acid; 3,5‐Hdmtz, 3,5‐dimethyl‐1,2,4‐triazole; Hfmtz, 3‐methyl‐5‐trifluoromethyl‐1,2,4‐triazole; H_2_adb, 4,4′‐(anthracene‐9,10‐diyl)dibenzoic acid; H_3_btp‐^i^Pr, isopropyl benzene‐1,3,5‐triyltris(hydrogen phosphonate); optp, octaethyl pyrene‐1,3,6,8‐tetraphosphonate; H_2_tmbpd, 3,3′,5,5′‐tetrakis(methylthio)biphenyl‐4,4′‐dicarboxylic acid; H_2_CH_3_PhTDC, 3‐methyl‐4‐phenylthieno[2,3‐b]thiophene‐2,5‐dicarboxylic acid; H_2_tbip, 5‐*tert*‐butyl isophthalic acid; 3,5‐Hetz, 3,5‐diethyl‐1,2,4‐triazole; H_2_bbpdc, 4′‐*tert*‐butyl‐biphenyl‐3,5‐dicarboxylic acid; Hotf, trifluoromethanesulfonic acid; H_2_dmpc, 3,5‐dimethyl‐1*H*‐pyrazole‐4‐carboxylic acid; H_4_pptc, pentiptycene‐based tetracarboxylic acid; 2‐Hnim, 2‐nitroimidazole; Hbim, benzimidazole; 2‐Heim, 2‐ethylimidazole; H_2_tebpz, 3,3′,5,5′‐tetraethyl‐4,4′‐bipyrazole; 3,5‐Htftz, 3,5‐bis(trifluoromethyl)‐1,2,4‐triazole; 4,5‐Hdcim, 4,5‐dichloroimidazole; 1,3‐H_2_bdb, 1,3‐di(4‐carboxyphenyl)benzene; 2,7‐H_2_ndb, 2,7‐di(4‐carboxyphenyl)naphthalene; H_3_ctta, 5′‐(4‐carboxyphenyl)‐2′,4′,6′‐trimethyl‐[1,1′:3′,1″‐terphenyl]‐4,4″‐dicarboxylic acid; H_3_ttna, 6,6′,6″‐(2,4,6‐trimethylbenzene‐1,3,5‐triyl)tris(2‐naphthoic acid); H_8_tdhb, 3,3′,5,5′‐tetrakis(3,5‐dicarboxyphenyl)‐2,2′,4,4′,6,6′‐hexamethylbiphenyl; 2‐Hcim, imidazole‐2‐carboxaldehyde; 5‐Hbbim, 5‐bromobenzimidazol; 5‐Hcbim, 5‐chlorobenzimidazole; 5‐Hmbim, 5‐methylbenzimidazole; Hbtz, 1*H*‐benzotriazole; RR‐H_2_cam, (1*R*,3*R*)‐1,2,2‐trimethyl‐1,3‐cyclopentanedicarboxylic acid; H_2_bim, 1,2‐bis((5*H*‐imidazol‐4‐yl)methylene)hydrazine; 2‐H_2_bmim, 1,2‐bis((2‐methyl‐1*H*‐imidazol‐4‐yl)methylene)hydrazine; DIFP, diisopropylfluorophosphate; DES, diethylsulfide; H_4_tcpp, tetrakis(4‐carboxyphenyl)porphyrin

^b)^ *S*_BET_ and *S*_L_ stand for the BET and Langmuir surface areas, respectively

^c)^ *V*_p_ stands for the pore volume

^d)^ *d*_a_ stands for the size of the aperture or channel

^e)^ *d*_p_ stands for the size of the cavity

^f)^ $C_{{\text{H}}_{2}\text{O}}$ stands for the water adsorption capacity near saturation pressure and room temperature unless otherwise specified

^g)^ α stands for the relative pressure at which half of the water adsorption capacity at saturation pressure is reached; $C_{{\text{H}}_{2}\text{O}}$ and α are obtained by reference values or by scanning the reported adsorption isotherm with the software ScanIt

^h)^ CA stands for the water contact angle

^i)^ Qualitative data were not provided

^j)^ $C_{{\text{H}}_{2}\text{O}}$ at 0.7 *P*/*P*_0_

^k)^ $C_{{\text{H}}_{2}\text{O}}$ at 0.63 *P*/*P*_0_.

### 3.1 Presynthetic Ligand Design and Functionalization

#### 3.1.1 Carboxylic Acids

**Scheme 1:**
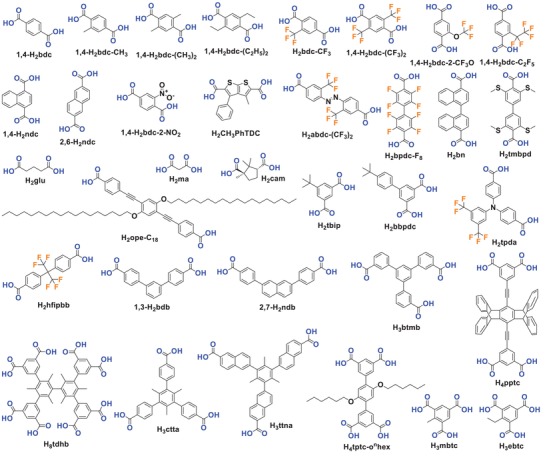
Carboxylic acid ligands used for constructing hydrophobic MOFs.

The carboxylic acid ligands used for constructing reported hydrophobic MOFs are summarized in **Scheme** 1. The ligand 1,4‐benzenedicarboxylic acid (1,4‐H_2_bdc), a representative ditopic carboxylic acid ligand, is one of the most frequently used ligands for the construction of MOFs. Many MOFs, including the well‐known [Zn_4_O(1,4‐bdc)_3_] (MOF‐5), [M(OH)(1,4‐bdc)] (MIL‐53, M = Cr, Al, Fe), MIL‐101(Cr), [Zr_6_O_4_(OH)_4_(1,4‐bdc)_6_] (UiO‐66), [ZrO(1,4‐bdc)] (MIL‐140A), and [In(OH)(1,4‐bdc)] (MIL‐68(In)), are synthesized from this ligand. Although built from the same ligand, these MOFs show quite different degrees of hydrophobicity. MOF‐5 and MIL‐53 are commonly regarded as hydrophilic MOFs, according to the sensitivity of MOF‐5 to water^161^ and the water adsorption isotherm of MIL‐53.^103^ From their water adsorption isotherms,^102^, ^104^, ^162^ which all show relatively gradual increasing uptakes at low‐*P*/*P*_0_ ranges, MIL‐101(Cr) and UiO‐66 are regarded to be slightly hydrophobic, regardless of the presence of some hydrophilic sites (OH^−^ or F^−^) on their inorganic secondary building units (SBUs). Similar to UiO‐66, MIL‐140A is synthesized by the solvothermal reaction of ZrCl_4_ with 1,4‐H_2_bdc, except that the reaction temperature is higher. Surprisingly, the structure of MIL‐140A has been found to be very different from that of UiO‐66. There are less hydrogen‐bond acceptor or donor sites on the pore surface of MIL‐140A, and it is thus regarded as a hydrophobic MOF.^71^ This conclusion is supported by the water adsorption isotherms, where the uptakes nearly linearly increase with the increasing of *P*/*P*_0_. In contrast, the pores of UiO‐66 are almost fully occupied by water at 0.5 *P*/*P*_0_ according to its water adsorption isotherm.^104^, ^162^ A typical type V water adsorption isotherm at room temperature has been observed for MIL‐68(In).^103^ Almost no water is adsorbed below 0.55 *P*/*P*_0_, at which pore filling starts to occur. This type of water adsorption isotherm is commonly found for hydrophobic carbon‐based adsorbents.

Many 1,4‐H_2_bdc‐derived ligands with one or more hydrophobic substituents have been used to construct MOFs.^163^, ^164^, ^165^, ^166^, ^167^ Wu et al. reported a MOF‐5 analogue, Banasorb‐22, built from the ligand 2‐trifluoromethoxy terephthalic acid (1,4‐H_2_bdc‐2‐CF_3_O).^72^ It was expected that the introduction of the strongly water‐repelling trifluoromethoxy groups (CF_3_O—) into the MOF would enhance its stability toward water. After MOF‐5 was treated by the steam of boiling water for several minutes, its BET surface area decreased from 2365 to 50 m^2^ g^−1^; however, the BET surface area of Banasorb‐22 only decreased from 1113 to 210 m^2^ g^−1^ after one week under the same treatment conditions.

**Figure 5:**
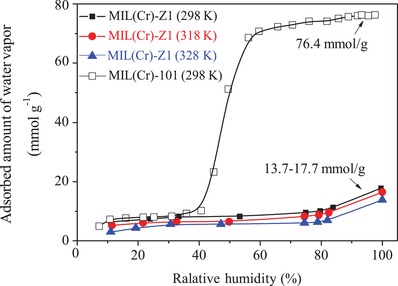
Water adsorption isotherms for MIL(Cr)‐Z1 and MIL(Cr)‐101. Adapted with permission.^105^ Copyright 2017 Elsevier.

Akiyama et al. investigated the water sorption properties of MIL‐101(Cr) and three analogues, MIL‐101‐NO_2_, MIL‐101‐NH_2_, and MIL‐101‐SO_3_H, obtained by —NO_2_, —NH_2_, and —SO_3_H substituted 1,4‐H_2_bdc ligands.^102^ In the water adsorption isotherm of MIL‐101(Cr), the uptake gradually increases at low pressures below 0.4 *P*/*P*_0_ and abruptly increases from 0.4 to 0.6 *P*/*P*_0_ in two steps, which were assigned to the pore filling of its 29 and 34 Å cages, respectively. The water uptake of MIL‐101(Cr) at 0.6 *P*/*P*_0_ reached up to 1200 mg g^−1^. The water adsorption isotherm profiles of MIL‐101‐NO_2_, MIL‐101‐NH_2_, and MIL‐101‐SO_3_H are similar to that of MIL‐101(Cr). However, the pore filling pressures of MIL‐101‐NH_2_ and MIL‐101‐SO_3_H shifted to lower *P*/*P*_0_, and the uptakes of MIL‐101‐NO_2_ were lower than those of MIL‐101(Cr) in the whole range of pressures tested. The isosteric heats of water adsorption for the four tested MOFs were in the order of MIL‐101‐NO_2_ < MIL‐101(Cr) < MIL‐101‐NH_2_ ≈ MIL‐101‐SO_3_H, suggesting that the hydrophobicity (hydrophilicity) of MIL‐101(Cr) could be tuned by introducing —NO_2_ (—NH_2_, —SO_3_H) groups onto its pore surface (although its pore size and surface area were reduced). Zhu et al. reported a MIL‐101(Cr) analog, [Cr_3_O(H_2_O)_2_F(1,4‐ndc)_3_] (MIL(Cr)‐Z1), obtained by using the ligand 1,4‐naphthalenedicarboxylic acid (1,4‐H_2_ndc).^105^ The water adsorption isotherm of MIL(Cr)‐Z1 showed an uptake of 17.7 mmol g^−1^ (318.6 mg g^−1^) at 0.99 *P*/*P*_0_ and 298 K (**Figure** 5), substantially lower than that of MIL‐101(Cr) (76.4 mmol g^−1^, 1375 mg g^−1^) under similar conditions,^76^ indicating that the framework hydrophobicity was significantly improved by replacing the ligand 1,4‐bdc^2−^ with 1,4‐ndc^2−^.

Cmarik et al. investigated the water adsorption properties of UiO‐66 and four analogues, UiO‐66−NH_2_, UiO‐66−2,5‐(OMe)_2_, UiO‐66−NO_2_, and UiO‐66−1,4‐Naphyl, which were obtained from 1,4‐H_2_bdc ligands substituted with —NH_2_, —OMe, —NO_2_, and naphthyl (—Naphyl) groups, respectively.^104^ Compared to that of UiO‐66, the water adsorption isotherms of for UiO‐66—NH_2_, UiO‐66—2,5‐(OMe)_2_, and UiO‐66—NO_2_ showed similar uptakes at 0.9 *P*/*P*_0_, and increased uptakes below 0.3 *P*/*P*_0_, indicating that the introduction of —NH_2_, —OMe, —NO_2_ groups enhanced the affinity between the MOFs and water. The water uptakes of UiO‐66—1,4‐Naphyl were significantly lower than those of UiO‐66−NH_2_, UiO‐66—2,5‐(OMe)_2_, and UiO‐66—NO_2_ at all pressures, but slightly higher than those of UiO‐66 only at pressures below 0.2 *P*/*P*_0_. This observation was explained by the pore size of UiO‐66−1,4‐Naphyl being reduced compared to that of UiO‐66, which increased the adsorption potential; however, full pore filling was depressed at higher *P*/*P*_0_ due to the introduction of hydrophobic naphthyl groups. UiO‐66−1,4‐Naphyl was thus regarded as a promising adsorbent for gas separations under humid conditions. Yu et al. studied the water adsorption properties of 6 UiO‐66 analogues, UiO‐66D‐(CF_3_)_2_, UiO‐66‐(C_2_H_5_)_2_, UiO‐66‐C_2_F_5_, UiO‐66‐(CF_3_)_2_, UiO‐66‐CH_3_, UiO‐66‐(CH_3_)_2_, and UiO‐66‐CF_3_, obtained by alkyl‐ and perfluoroalkyl‐functionalized 1,4‐H_2_bdc ligands.^87^ The authors also showed that the water adsorption behaviors were affected by both the hydrophobicity of the functional group and the effect of pore reduction.

As extended versions of 1,4‐H_2_bdc, some long, linear dicarboxylic acid ligands have been used to construct hydrophobic MOFs, such as H_2_tmbpd,^109^ H_2_abdc‐(CF_3_)_2_,^87^ H_2_ope‐C_18_,^110^ H_2_CH_3_PhTDC,^111^ and H_2_bpdc‐F_8_.^112^ Many angular dicarboxylic acid ligands have also been reported for use in hydrophobic MOFs.^88^, ^113^, ^114^, ^115^, ^116^, ^117^, ^118^, ^119^, ^120^ As early as 2004, Pan et al. reported a Cu_2_ paddle‐wheel SBU based MOF, [Cu(hfipbb)(H_2_hfipbb)_0.5_], with the angular ligand H_2_hfipbb.^116^, ^117^ In this MOF, there are irregular‐shaped small hydrophobic channels with alternating large chambers (7.3 Å) and small necks (3.2 Å) between the chambers. It was demonstrated that [Cu(hfipbb)(H_2_hfipbb)_0.5_] favored the adsorption of propane and butane but not pentane or other higher normal and branched hydrocarbons. The same research group also reported a hydrophobic MOF, Zn(tbip), by the angular ligand H_2_tbip.^113^ The 1D channels of Zn(tbip) are lined with butyl groups at a size of 4.5 Å, accounting for 17.7% of the crystal volume. The material's hydrophobicity was confirmed by water adsorption, which showed a very low uptake (less than 1 mg g^−1^) at 0.65 *P*/*P*_0_ and 298 K, lower than that for pure silica ZSM‐5 (7 mg g^−1^) under similar conditions.

**Figure 6:**
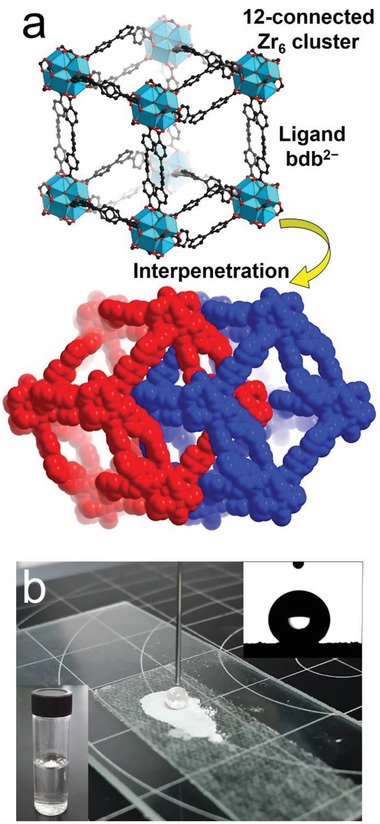
a) Twofold network interpenetration in the structure of BUT‐66. b) An optical photo of a water drop on BUT‐66 powder; inset: the water contact angle of BUT‐66 (upper right) and a photo of BUT‐66 crystals floating on the surface of water (bottom left). Adapted with permission.^88^ Copyright 2018 Elsevier.

Xie et al. recently reported two zirconium‐based MOFs, [Zr_6_O_4_(OH)_4_(1,3‐bdb)_6_] (BUT‐66) and [Zr_6_O_4_(OH)_4_(2,7‐ndb)_6_] (BUT‐67), by using the angular dicarboxylic acid ligands 1,3‐H_2_bdb and 2,7‐H_2_ndb, respectively.^88^ The MOFs contain 1D channels with a size of 6–7 Å, the wall of which is predominately made of the aromatic rings of ligands. The water adsorption studies at 298 K for BUT‐66 and BUT‐67 showed uptakes of 5.7 mmol g^−1^ (103 mg g^−1^) and 6.9 mmol g^−1^ (124 mg g^−1^) at 0.95 *P*/*P*_0_, lower than the predicted water uptakes (420 and 380 mg g^−1^) assuming that their pore volumes (determined from N_2_ adsorption at 77 K) are fully filled with crystallized water (density: 0.9168 g cm^−3^). The MOFs were thus regarded as adsorbents with moderately hydrophobic channels. The authors proposed that a 2‐fold network interpenetration of their structures contributed to the hydrophobicity of the channel surfaces of the MOFs (**Figure** 6a), where some hydrophilic surfaces in one network were covered by the hydrophobic phenyl groups of ligands in the other network. In addition, it was found that BUT‐66 and BUT‐67 could float on the surface of water, although their densities calculated from single‐crystal structures were higher than the density of water (1 g cm^−3^), suggesting a high hydrophobicity of the external crystal surface of the MOFs. The external hydrophobicity was verified by water contact angle measurements, which gave 143° and 138° water contact angles (Figure 6b).

**Figure 7:**
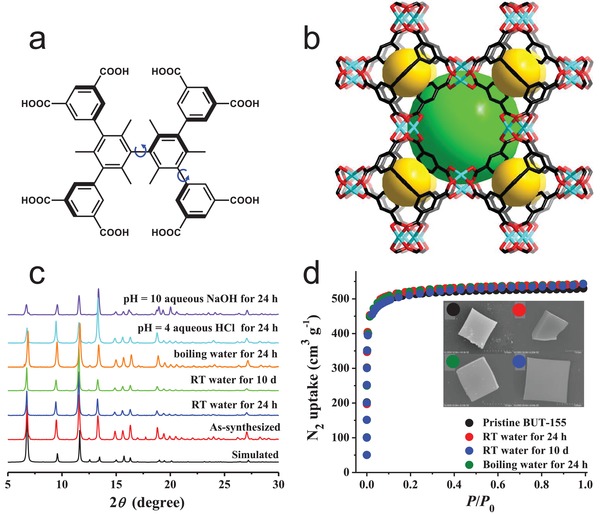
a) Molecular structure of the octatopic carboxylic acid ligand H_8_tdhb. b) The framework structure of BUT‐155 built from Cu_2_ paddle‐wheel SBUs and tdhb^4−^. c) PXRD patterns, d) N_2_ adsorption isotherms recorded at 77 K, and SEM images (d, inset) for BUT‐155 samples after being treated under different conditions. Adapted with permission.^128^ Copyright 2017 American Chemical Society.

MOFs synthesized from some tritopic, tetratopic, and octatopic carboxylic acid ligands have also shown characteristics of hydrophobicity.^121^, ^122^, ^123^, ^125^, ^126^, ^127^, ^128^ Cai et al. reported two Cu_2_ paddle‐wheel SBU based MOFs, [Cu_3_(mbtc)_2_] (CuMBTC) and [Cu_3_(ebtc)_2_] (CuEBTC), which were obtained from methyl‐ and ethyl‐ group‐functionalized H_3_btc ligands, H_3_mbtc and H_3_ebtc, respectively.^121^ The assembly of H_3_btc and Cu_2_ paddle‐wheel SBUs produces the well‐studied MOF [Cu_3_(btc)_2_] (HKUST‐1, also called CuBTC or MOF‐199). However, due to the steric impact of the alkyl groups, the carboxyl groups in H_3_mbtc and H_3_ebtc are not coplanar. As a result, both CuMBTC and CuEBTC are not isostructural to HKUST‐1 but still highly porous. Although the difference in pore volumes for the three MOFs (0.83, 0.79, and 0.65 cm^3^ g^−1^ for HKUST‐1, CuMBTC, and CuEBTC, respectively) is not significant, the water adsorption isotherms show an uptake of 184 mg g^−1^ for both CuMBTC and CuEBTC at 0.9 *P*/*P*_0_ and 298 K, considerably lower than that of HKUST‐1 (587 mg g^−1^) under the same conditions. It was pointed out that hydrophobic functional groups on pore surface could depress the strong adsorption preference of water for the MOFs, even there were open metal sites (coordinatively unsaturated metal sites). However, similar to HKUST‐1, CuMBTC and CuEBTC still degraded after exposure to water, according to powder X‐ray diffraction (PXRD) patterns. Recently, Chen et al. reported a Cu_2_ paddle‐wheel SBU based MOF, [Cu_4_(tdhb)] (BUT‐155), which was synthesized by an octatopic carboxylic acid ligand, H_8_tdhb.^128^ There exist six methyl groups, compelling the ligand to adopt a rigid tetrahedral backbone geometry (**Figure** 7a,b). Surprisingly, BUT‐155 showed a high porosity (BET surface area: 2070 m^2^ g^−1^ and pore volume: 0.82 cm^3^ g^−1^), as well as exceptional stability against water (even boiling water), which was verified by PXRD patterns, N_2_ adsorption studies, scanning electron microscopy (SEM) images and single‐crystal X‐ray diffraction (SXRD) studies for the BUT‐155 crystals after treatment in water for 10 days at room temperature or in boiling water for 24 h (Figure 7c,d). The authors proposed that a combined effect of the high connectivity and constrained geometry of the ligand, the hydrophobicity of the pore surface, and the electron donation contribution of the six methyl substituents of the ligand to Cu—O coordination bonds led to the unexpectedly high hydrolytic stability of BUT‐155, although copper(II) paddlewheel‐based MOFs were commonly considered to be intrinsically unstable in water.

Zhang et al. also reported a hydrophobic and water stable copper(II) paddlewheel‐based MOF, [Cu_2_(pptc)] (UPC‐21), obtained by a pentiptycene‐based tetratopic carboxylic acid ligand, H_4_pptc.^126^, ^127^ Due to the high hydrophobicity of the pentiptycene moieties in the ligands, UPC‐21 showed a high water contact angle (145°), and it could float on the surface of water for a long time without the collapse of its crystalline structure. However, the water adsorption isotherm for UPC‐21 was not reported. UPC‐21 was also highly porous (BET surface area: 1725 m^2^ g^−1^) when it was prepared by a so‐called “diauxic growth” method, whereby large pure crystals of UPC‐21 were formed in a filtrate after the reaction mixture was first reacted in an oven at 75 °C for 25 h, and the resultant small crystals were filtered off.

**Figure 8:**
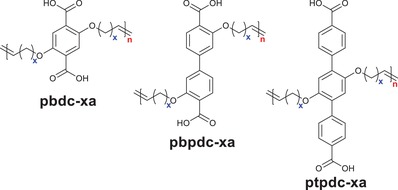
Polymer ligands used to prepare polyMOFs. The “x” in the names of the polymer ligands represents the number of methylene spacers between each H_2_bdc, H_2_bpdc or H_2_tpdc unit, while “a” denotes the acid form of the polymer ligands.^129^

Commonly, MOFs are prepared by small molecular ligands. Recently, Cohen and co‐workers demonstrated that certain linear organic polymers with aromatic dicarboxylic acids in their backbones could be used as ligands to synthesize highly crystalline and porous MOFs, called polyMOFs.^129^, ^168^, ^169^, ^170^ A series of polymer ligands were synthesized (**Figure** 8) and used to prepare polyMOFs with crystalline structures isostructural to MOF‐5, [Zn_2_(bdc)_2_(dabco)], [Zn_2_(bdc)_2_(4,4′‐bpy)], UiO‐66, UiO‐67, and UiO‐68, respectively. The polyMOFs showed a permanent porosity, as their parent MOF structures do, and some of these polyMOFs inherited the hydrophobicity of their polymer ligands. Moreover, compared to the parent MOFs, the polyMOFs exhibited an enhanced stability against water. For example, Zn‐pbdc‐8a showed a BET surface area of 856 m^2^ g^−1^ and a water contact angle of 112°. After being exposed in open air for 3 days, Zn‐pbdc‐8a retained its crystalline structure. In contrast, MOF‐5 degraded into another phase (MOF‐69c)^161^ after exposure in air for 1 day.

**Figure 9:**
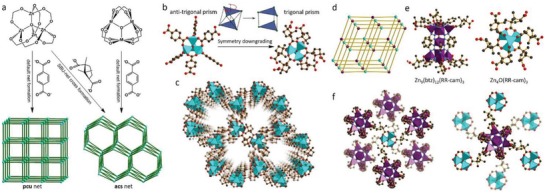
a) SBU‐net cross formation in CPM‐300. b) A comparison of the Zn_4_O(CO_2_)_6_ SBU in MOF‐5 and that in CPM‐300 showing a symmetry reduction from antitrigonal prism (octahedron) to trigonal prism, when the geometry of the ligands is taken into account. c) A perspective view of CPM‐300, showing the chiral hexagonal channels along the *c* axis. d) In CPM‐301, by introducing the [Zn_9_(btz)_12_]^6+^ cluster, the 6‐connected [Zn_9_(btz)_12_]^6+^ and [Zn_4_O]^6+^ clusters are arranged alternatingly to form a pcu net of CPM‐301. e) The [Zn_9_(btz)_12_]^6+^ and [Zn_4_O]^6+^ clusters in CPM‐301. f) The connection between the [Zn_4_O]^6+^ and [Zn_9_(btz)_12_]^6+^ clusters. Adapted with permission.^101^ Copyright 2018, American Chemical Society.

Polytopic carboxylic acids used for the construction of hydrophobic MOFs are mostly ligands with aromatic backbones, and examples constructed from aliphatic carboxylic acid ligands are rare. As early as 2003, Vaidhyanathan et al. reported a cadmium malonate [Cd(O_2_C‐CH_2_‐CO_2_)(H_2_O)] and a manganese glutarate [Mn(O_2_C‐(CH_2_)_3_‐CO_2_)], both possessing 1D hydrophobic channels with sizes of 6.95 and 6.64 Å, respectively. However, neither gas nor water vapor adsorption study was performed to confirm their permanent porosity and hydrophobicity. Recently, Zhao et al. reported two hydrophobic MOFs, [Zn_4_O(RR‐cam)_3_] (CPM‐300) and [Zn_4_O(RRcam)_3_][Zn_9_(btz)_12_(RR‐cam)_3_] (CPM‐301), synthesized from a pentane‐ring‐based chiral dicarboxylic acid, RR‐H_2_cam.^101^ CPM‐300 was a 6‐connected framework with the acs topology constructed by 6‐connected [Zn_4_O]^6+^ clusters and RR‐cam^2−^ ligands, and CPM‐301 was a binodal 6‐connected framework with the pcu topology constructed by 6‐connected [Zn_4_O]^6+^ and 6‐connected [Zn_9_(btz)_12_]^6+^ (with btz^−^ serving as terminal ligands) clusters bridged by RR‐cam^2−^ ligands (**Figure** 9). The N_2_ sorption study at 77 K revealed a moderate porosity for CPM‐300 with a BET surface area of 310.5 m^2^ g^−1^ and a Langmuir surface area of 429.9 m^2^ g^−1^. The hydrophobic natures of CPM‐300 and CPM‐301 were confirmed by water contact angle measurements, which gave contact angles of 138.7° and 145.0° for compressed pellets of the MOFs, respectively. In contrast, an initial contact angle of ≈17.0° was observed for MOF‐5, and the water droplet completely wet the MOF‐5 sample in less than 10 s. The water contact angle was also recorded for the ligand RR‐H_2_cam, which showed a high initial contact angle (114.5°), but the water droplet gradually wet the ligand sample within 1 min. Noteworthily, the [Zn_4_O]^6+^ cluster is well‐known for its poor stability toward water in MOF‐5; however, CPM‐300 and CPM‐301, built from the same cluster, show high stabilities, even in boiling water for 1–2 days. The authors proposed that small methyl groups on the chiral ligand RR‐cam^2−^ shielded the [Zn_4_O]^6+^ clusters from degradation by water, even at high temperatures.

#### 3.1.2 Azoles

**Scheme 2:**
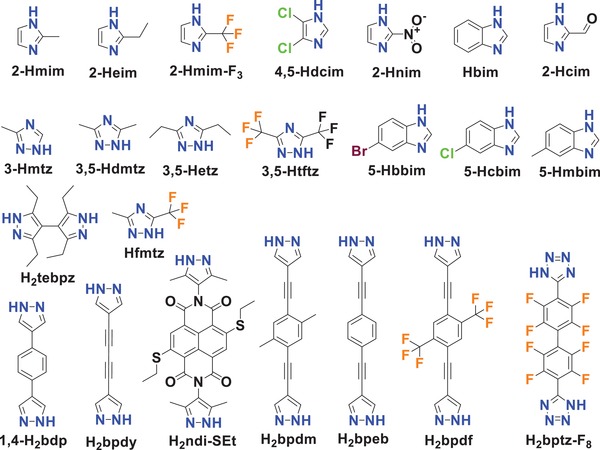
Azole acid ligands used for constructing hydrophobic MOFs.

Azole ligands, mostly imidazoles, polypyrazoles, triazoles, and tetrazoles, have been extensively used for the construction of MOFs,^15^, ^171^, ^172^ some of which are shown in **Scheme** 2. Many metal azolates are extremely hydrophobic with a high porosity. A representative example is ZIF‐8, also called MAF‐4,^173^, ^174^ which is also one of the most well‐known MOFs. ZIF‐8, a 3D zinc 2‐methylimidazolate with the SOD zeolitic topology, shows both a high porosity (BET surface area: 1870 m^2^ g^−1^ and pore volume: 0.67 cm^3^ g^−1^)^77^ and high stability under nonacidic conditions.^174^, ^175^, ^176^ Moreover, ZIF‐8 is highly hydrophobic, which was confirmed by water adsorption studies. Kaskel et al. first reported the water adsorption isotherm for ZIF‐8 at 25 °C, which showed a very low uptake of 10 cm^3^ g^−1^ (8 mg g^−1^) at *P*/*P*_0_ = 0.8, and a final steep increase to 150 cm^3^ g^−1^ (121 mg g^−1^) at *P*/*P*_0_ = 0.97.^76^ Several other works have also reported water adsorption isotherms for ZIF‐8. The water adsorption isotherm for ZIF‐8 at 25 °C reported by Zhang et al. showed a very low water uptake at *P*/*P*_0_ < 0.9 and an uptake of 32 cm^3^ g^−1^ (26 mg g^−1^) at *P*/*P*_0_ = 0.99.^77^ Chance and coworkers reported the water adsorption isotherm for ZIF‐8 at 35 °C, which indicated a low uptake of 10 mg g^−1^ at *P*/*P*_0_ = 0.98.^79^ These results all demonstrated the high hydrophobicity of the internal pore surface of ZIF‐8, although, there were some differences among the water vapor adsorption isotherms, which should originate from the differences in those ZIF‐8 samples obtained by distinct synthesis and/or activation methods. However, it should be mentioned that most ZIF‐8 samples reported in the literature have hydrophilic exterior crystal surfaces, as indicated by their water contact angles (0°–56°).^78^, ^176^, ^177^

Some other reported metal imidazolates have also been proven highly hydrophobic. Lively et al. found that ZIF‐71 (RHO topology, formulated as [Zn(4,5‐dcim)_2_]) synthesized in methanol at room temperature shows a high porosity and high hydrophobicity.^136^ The BET surface area of the ZIF‐71 sample was 1186.5 m^2^ g^−1^, evidently higher than that of the ZIF‐71 sample synthesized from DMF by a solvothermal reaction (652 m^2^ g^−1^).^135^ The water adsorption isotherm of ZIF‐71 revealed a very low uptake of 0.3 mmol g^−1^ (5.4 mg g^−1^) at *P*/*P*_0_ = 0.95 and 35 °C. Later, the same team reported the water adsorption isotherms of ZIF‐8, ZIF‐71, and ZIF‐90 (formulated as [Zn(2‐cim)_2_]).^79^ Type III isotherms with low water uptakes, even at near saturation pressure, were observed for ZIF‐8 and ZIF‐71. At high *P*/*P*_0_, the water uptakes of ZIF‐8 were slightly higher than those of ZIF‐71, indicating the stronger affinity toward water of the former. This result is not surprising because the cages in ZIF‐71 (16.5 Å) are larger than those in ZIF‐8 (11.6 Å). The water adsorption isotherm of ZIF‐90 showed low uptakes below 0.3 *P*/*P*_0_ and pore filling at higher pressures, with an uptake of 18.6 mmol g^−1^ (335 mg g^−1^) at *P*/*P*_0_ = 0.98. The authors suggested that ZIF‐90 was less hydrophobic than ZIF‐8 and ZIF‐71 due to the existence of the more‐hydrophilic carbonyl group in the ligands of ZIF‐90, which was also a SOD‐type zeolitic framework like ZIF‐8.

He et al. reported a zinc 2‐ethylimidazolate with the RHO zeolitic topology, MAF‐6, showing a large surface area (Langmuir surface area: 1695 m^2^ g^−1^), high pore volume (0.61 cm^3^ g^−1^), large pore (*d* = 18.4 Å), and aperture size (*d* = 7.6 Å), and highly hydrophobic internal pore and external crystal surfaces.^78^ The water uptake of MAF‐6 at *P*/*P*_0_ = 0.97 and 298 K was only 0.90 mmol g^−1^ (16.2 mg g^−1^). It was also found that powder samples of MAF‐6 could float on the water surface. The MOF's hydrophobic external crystal surface was confirmed by water contact angle measurement, which showed a large contact angle of 143°. Surprisingly, MAF‐5,^139^ an isomer of MAF‐6 with the ANA zeolitic topology, showed a highly hydrophilic external crystal surface with a contact angle of 0°.

**Figure 10:**
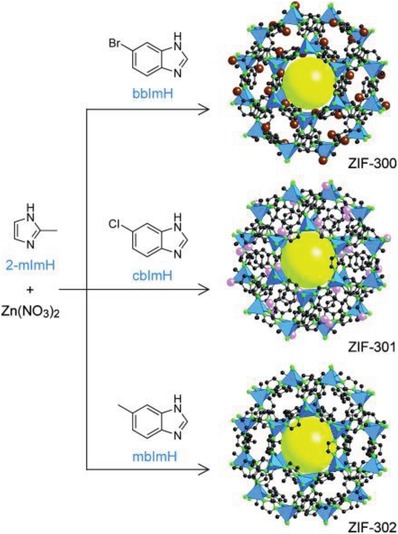
The ligands and crystal structures for ZIF‐300, ZIF‐301 and ZIF‐302. Adapted with permission.^138^ Copyright 2014, John Wiley and Sons.

Nguyen et al. reported three hydrophobic zinc imidazolates, [Zn(2‐mim)_0.86_(5‐bbim)_1.14_] (ZIF‐300), [Zn(2‐mim)_0.94_(5‐cbim)_1.06_] (ZIF‐301), and [Zn(2‐mim)_0.67_(5‐mbim)_1.33_] (ZIF‐302), acquired by using two distinct imidazole ligands for each (2‐Hmim/5‐Hbbim, 2‐Hmim/5‐Hcbim, and 2‐Hmim/5‐Hmbim), which are 3D framework structures with the chabazite (CHA) zeolite topology (**Figure** 10).^138^ Ar gas‐adsorption measurements at 87 K showed the moderate porosities of the three ZIFs with BET surface areas of 420, 680, and 240 m^2^ g^−1^. PXRD patterns confirmed the high stability of ZIF‐300, ZIF‐301, and ZIF‐302 in boiling water for 7 days. Water adsorption measurements at 298 K were carried out and gave type III isotherms for all three ZIFs with low uptakes (6, 5.8, and 4.5 mg g^−1^) at *P*/*P*_0_ ≈ 0.8.

**Figure 11:**
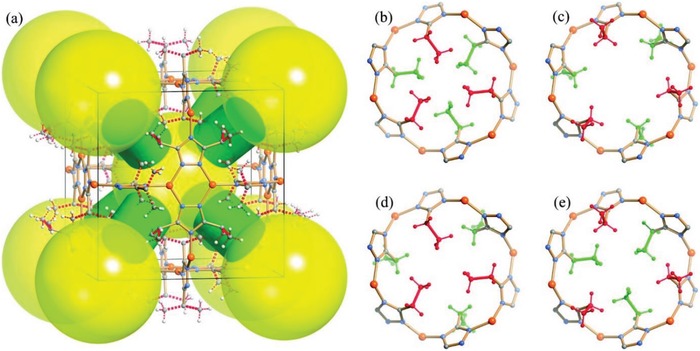
a) The framework of MAF‐2 with large cages (large yellow spheres) interconnected by small windows (green columns). The window size is kinetically controlled by the rotation/swing motions of the pendant ethyl groups. b−e) A perspective view of four representative channel states: double closed, double open, closed‐open, and open‐closed (the ethyl groups are colored in red and green to highlight the two different gates). Adapted with permission.^143^ Copyright 2008, American Chemical Society.

Some highly hydrophobic MOFs have been prepared from 3‐substituted or 3,5‐substituted 1,2,4‐triazoles. In 2008, Zhang and Chen reported a cuprous 3,5‐diethyl‐1,2,4‐triazolate, [Cu(3,5‐etz)] (MAF‐2), having a 3D framework with the nbo topology and so‐called “kinetically controlled” hydrophobic channels.^143^ The large cages in MAF‐2 are interconnected by small windows defined by six pendant ethyl groups (**Figure** 11a). Those windows are blocked by the ethyl groups below a critical or onset adsorption temperature and are tuned to be open at higher temperatures (Figure 11b–e). The water adsorption isotherm of MAF‐2 at 298 K showed a very low uptake (3 mg g^−1^) at *P*/*P*_0_ = 0.97, which was attributed to particle surface adsorption on uncoordinated defects. Although the pores in MAF‐2 were closed for water, they could adsorb large amounts of small organic molecules such as MeOH, EtOH, and MeCN. It was also found that MAF‐2 could adsorb benzene, but cyclohexane could not diffuse into its “kinetically controlled” pores. The authors recently reported a partially fluorinated Cu triazolate, MAF‐52, containing the ligand Hfmtz.^145^ MAF‐52 is a 5‐connected 3D framework (bnn topology) consisting of Cu_5_(µ_5_‐Cl) clusters interconnected by fmtz^−^ ligands. It was demonstrated that MAF‐52 was porous (Langmuir surface area: 1023 m^2^ g^−1^, and pore volume: 0.365 cm^3^ g^−1^), highly chemically stable (tolerant to water and aqueous solutions with pH ranging 2–13), and highly hydrophobic, as suggested by its water contact angle (148°) and low water uptake (0.8 mg g^−1^) at *P*/*P*_0_ = 0.95 and 25 °C. The same group also reported a zinc 3‐methyl‐1,2,4‐triazolate, [Zn(3‐mtz)_2_] (MAF‐7), which was an SOD‐type zeolitic framework structure similar to MAF‐4/ZIF‐8 but with one uncoordinated triazolate N on the pore surface. The authors determined that MAF‐7 was also stable in water, similar to MAF‐4/ZIF‐8, and the N_2_ adsorption measurements showed the close porosity of MAF‐7 to that of MAF‐4/ZIF‐8 (Langmuir surface area: 1870 m^2^ g^−1^, and pore volume: 0.67 cm^3^ g^−1^). The water adsorption isotherm of MAF‐7 was a type V isotherm, where low uptakes (<20 mg g^−1^) observed at *P*/*P*_0_ < 0.24 were followed by pore filling at higher *P*/*P*_0_ (with an uptake of 380 mg g^−1^ reached at *P*/*P*_0_ = 0.98). Clearly, MAF‐7 is more hydrophilic than MAF‐4 due to the presence of uncoordinated triazolate N atoms on its pore surface.

Yang et al. reported a highly hydrophobic Ag(I)‐based MOF named FMOF‐1 (formulated as {Ag_2_[Ag_4_(3,5‐tftz)_6_]}) obtained by the perfluorinated ligand 3,5‐Htftz.^80^, ^130^ The fluorous MOF has a 3D framework consisting of 6‐connected tetranuclear [Ag_4_(3,5‐tftz)_6_] clusters linked by 3‐coordinated Ag(I) centers with cylindrical channels in a semirectangular shape (≈12.2 Å × 7.3 Å) and diamond‐shaped small cavities (≈6.6 Å × 4.9 Å). The hydrophobic —CF_3_ groups of the perfluorinated ligand 3,5‐tftz^−^ are pointed into the channels. The N_2_ adsorption study revealed a BET surface area of 810.5 m^2^ g^−1^ and a pore volume of 0.324 cm^3^ g^−1^ for FMOF‐1. The high hydrophobicity of FMOF‐1 was confirmed by a water adsorption study, which showed a negligible uptake at *P*/*P*_0_ = 0.9, regardless of its large channel size. The negligible water adsorption of FMOF‐1 was further confirmed by the single‐crystal X‐ray diffraction analysis of a water‐soaked single crystal of FMOF‐1, which showed a unit cell volume (6999 Å^3^) comparable to that of a guest‐free FMOF‐1 crystal (7063.0 Å^3^). The authors also confirmed the absence of water in the channels of FMOF‐1 by IR measurement, which showed no O–H stretches from H_2_O molecules for a water‐treated sample, as observed for the guest‐free FMOF‐1 sample. Moreover, the authors found that FMOF‐1 possessed a superhydrophobic external surface, as indicated by the high water contact angle up to 158° of a pellet sample of FMOF‐1.^131^

**Figure 12:**
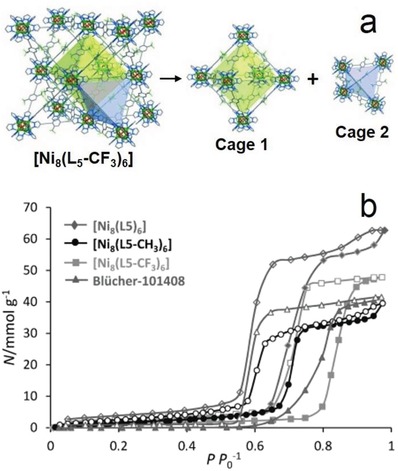
a) The 3D framework of [Ni_8_(L_5_‐CF_3_)_6_] with two types of cages: octahedral (yellow polyhedron) and tetrahedral (gray polyhedron) cages. b) H_2_O adsorption isotherms at 298 K for the isoreticular [Ni_8_(L_5_‐R)_6_] series and the activated carbon Blücher‐101408. Solid symbols denote adsorption, and open symbols denote desorption. Adapted with permission.^140^ Copyright 2013, John Wiley and Sons.

Padial et al. reported a series of MOFs named as [Ni_8_(L)_6_] (L stands for ligand) obtained by using the bipyrazole ligands 1,4‐H_2_bdp (L_3_ in the reference), H_2_bpdy (L_4_), H_2_bpeb (L_5_), H_2_bpdm (L_5_‐CH_3_), and H_2_bpdf (L_5_‐CF_3_).^140^ [Ni_8_(L)_6_] are isoreticular 12‐connected frameworks of the fcu topology with [Ni_8_(OH)_4_(H_2_O)_2_] as 12‐connected SBUs, which are bridged by the bipyrazole ligands (**Figure** 12a). The framework structures of the [Ni_8_(L)_6_] series are analogous to the well‐known UiO series with 12‐connected [Zr_6_O_4_(OH)_4_] SBUs.^178^ The [Ni_8_(L)_6_] series exhibited high porosities (BET surface areas: 205–2215 m^2^ g^−1^) and high stabilities in water and basic media at room temperature for 24 h. In contrast, two isoreticular MOFs, [Ni_8_(L_1_)_6_] and [Ni_8_(L_2_)_6_], synthesized from mixed pyrazolate/carboxylate linkers (H_2_L_1_ = 1*H*‐pyrazole‐4‐carboxylic acid, and H_2_L_2_ = 4‐(1*H*‐pyrazole‐4‐yl)benzoic acid) were not stable to moisture. Type V water adsorption isotherms were observed for [Ni_8_(L_3_)_6_], [Ni_8_(L_4_)_6_], [Ni_8_(L_5_)_6_], [Ni_8_(L_5_‐CH_3_)_6_], and [Ni_8_(L_5_‐CF_3_)_6_], with partial pressures corresponding to the beginning of water vapor condensation in the range of 0.30 to 0.80 *P*/*P*_0_ (Figure 12b). Especially, [Ni_8_(L_5_‐CF_3_)_6_], containing fluorinated methyl groups, was considered to be more hydrophobic than prototypical mesoporous MOFs such as MIL‐100(Fe) and MIL‐101(Cr) and even the highly hydrophobic, commercially available, activated carbon Blücher‐101408, which was employed in the state‐of‐the‐art Saratoga filtering systems.

Wang et al. also reported a bipyrazole‐ligand‐based hydrophobic MOF, [Cu_2_(tebpz)] (Cu‐tebpz), which is a rare (3,4)‐connected framework structure built from triangular Cu_3_(tebpz)_3_ and tetrahedral Cu_4_(tebpz)_4_ SBUs.^141^ There are three types of 1D channels with sizes of 4.6 × 4.7, 6.5 × 6.5, and 6.3 × 13.2 Å^2^, and dangling tetraethyl groups are located on the interior surface of the channels. The MOF showed a moderate porosity with a BET surface area of 576 m^2^ g^−1^ and a pore volume of 0.37 cm^3^ g^−1^. Like many other stable metal bipyrazolate MOFs,^179^, ^180^, ^181^, ^182^, ^183^ Cu‐tebpz was proven stable in boiling solvent (THF, toluene, hexane, or DMSO), water, and even acidic (1 × 10^−3^ m HCl) and basic (1 × 10^−3^ m NaOH) aqueous solutions for 24 h by PXRD patterns. The hydrophobicity of Cu‐tebpz was confirmed by its water adsorption isotherm, which showed almost negligible uptakes, even at high humidity up to 0.9 *P*/*P*_0_ at 298 K, but an abundance of benzene and n‐hexane could be adsorbed by the MOF under the same conditions.

#### 3.1.3 Mixed Ligands and Ligands with O and N Donors

**Scheme 3:**
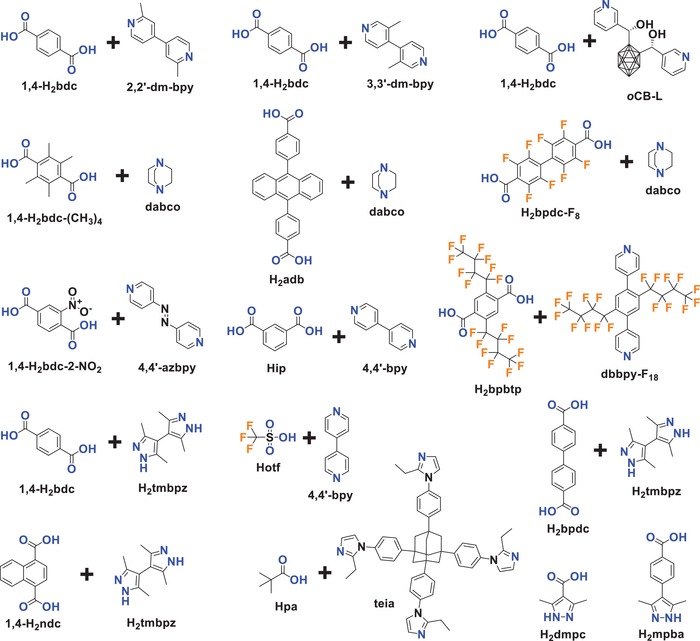
Mixed ligands and ligands with O and N donors used for constructing hydrophobic MOFs.

Some hydrophobic MOFs have been synthesized from more than one type of ligand or by one type of ligand with different coordination donors, mostly O and N atoms, as shown in **Scheme** 3.^112^, ^148^, ^149^, ^150^, ^152^, ^154^, ^184^

**Figure 13:**
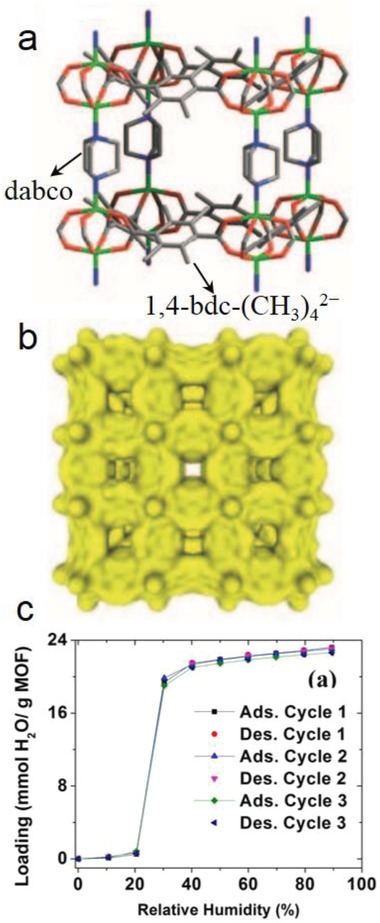
a) The framework structure, b) 1D small channels, and c) cyclic water adsorption isotherms at 298 K and 1 bar for DMOF‐TM2. Adapted with permission.^153^, ^185^ Copyright 2005, John Wiley and Sons for panels (a) and (b); and Copyright 2013, American Chemical Society for panel (c).

Jasuja et al. reported that a zinc‐base MOF with a pillared‐layer structure, [Zn_2_(1,4‐bdc‐(CH_3_)_4_)_2_(dabco)] (DMOF‐TM2), was synthesized from the dicarboxylic acid ligand 1,4‐H_2_bdc‐(CH_3_)_4_ and the neutral ligand dabco and possessed a high porosity, hydrophobicity and stability toward water.^153^ DMOF‐TM2 was first reported by Kim and coworkers.^185^ It is a mixed‐ligand MOF in which paddle‐wheel‐shaped dimeric zinc clusters are connected by 1,4‐bdc‐(CH_3_)_4_^2−^ ligands, forming 2D layers, and the 2D layers are pillared by the neutral dabco ligands, resulting in an open framework with 1D channels of 3.5 Å (**Figure** 13a,b). The N_2_ adsorption measurement at 77 K revealed the high porosity of DMOF‐TM2, showing a BET surface area of 1050 m^2^ g^−1^ and a pore volume of 0.51 cm^3^ g^−1^. DMOF‐TM2 was proven stable toward moisture (90% RH), as confirmed by the PXRD patterns and BET surface areas measured before and after water exposure. The authors confirmed that DMOF‐TM2 maintained its crystallinity after exposure to lab air for approximately one year. When the dicarboxylic ligand 1,4‐bdc‐(CH_3_)_4_^2−^ in DMOF‐TM2 was replaced by 1,4‐bdc^2−^ and/or its fluorine‐ or methyl‐substituted derivatives, 1,4‐bdc‐CH_3_^2−^, 1,4‐bdc‐(CH_3_)_2_^2−^, and 1,4‐bdc‐F_4_^2−^, six isostructural pillared‐layer MOFs were obtained, and they almost completely lost their crystallinity after exposure to moisture. DMOF‐TM2 showed type V water adsorption isotherms with low uptakes below 0.2 *P*/*P*_0_ and pore filling at higher partial pressures (e.g., 412 mg g^−1^ at 0.9 *P*/*P*_0_), indicating the moderately hydrophobic nature of the MOF. Three cycles of water adsorption/desorption measurements were performed, and no change in the isotherms (Figure 13c) was noted. While PXRD patterns and BET surface area measurements also confirmed no change in the crystal structure after the three adsorption/desorption cycles. According to experimental results and molecular simulations, the authors concluded that the improved moisture stability of DMOF‐TM2 over that of the other isostructural MOFs originated from the shielding of the carboxylate oxygen in the structure by nearby hydrophobic methyl groups, which prevented hydrogen‐bonding interactions and subsequent structural transformations occurring, and as a result, the electrophilic zinc atoms in this structure became inaccessible to the nucleophilic oxygen atoms in water, thus, preventing any ligand displacement hydrolysis reactions occurring.

Ma et al. reported the two MOFs, [Zn_2_(1,4‐bdc)_2_(2,2′‐dmbpy)] (SCUTC‐18) and [Zn_2_(1,4‐bdc)_2_(3,3′‐dm‐bpy)] (SCUTC‐19),^146^ isostructural to [Zn_2_(1,4‐bdc)_2_(4,4′‐bpy)] (MOF‐508),^186^ a pillared‐layer MOF built from layers of 1,4‐bdc^2−^‐bridged paddle‐wheel Zn_2_ clusters with neutral 4,4′‐bpy ligands as pillars. In SCUTC‐18 and SCUTC‐19, the pillars involve 4,4′‐bpy that are methyl‐substituted at *ortho*‐ and *meta*‐positions with respect to the pyridine atoms, namely, 2,2′‐dm‐bpy, 3,3′‐dm‐bpy, respectively. The three MOFs are all 2‐fold interpenetrated structures with 1D channels of 6.4, 6.5, and 8.0 Å. N_2_ sorption measurements showed the moderate porosity of the MOFs, and the BET surface areas were 398, 523, and 458 m^2^ g^−1^ for MOF‐508, SCUTC‐18, and SCUTC‐19, respectively. The authors determined that MOF‐508 was unstable in air and that its structure fully collapsed after moisture exposure for one week; however, SCUTC‐18 and SCUTC‐19 showed improved stabilities in ambient air. After exposure to air for 7 days, no new peaks appeared for SCUTC‐19, but all the peak intensities of SCUTC‐19 decreased. For SCUTC‐18, no new peaks or apparent loss in peak intensity of PXRD patterns was observed after being exposed to air for up to 30 days. N_2_ sorption measurements were also carried out after the MOFs were exposed to air for one week, and the BET surface areas of MOF‐508 and SCUTC‐19 deceased significantly (87% and 55% reduction, respectively). However, only a slight change was observed for SCUTC‐18 (from 523 to 506 m^2^ g^−1^, maintaining 97%). This result was interpreted as the hydrophobic methyl groups at the *ortho*‐positions of the coordinating nitrogen atoms of the pillar ligands were close to the Zn centers in SCUTC‐18, which shielded the Zn ions from attack by water molecules; however, the methyl groups at the *meta*‐positions are far from the metal centers in SCUTC‐19, which resulted in a weak protection effect.

**Figure 14:**
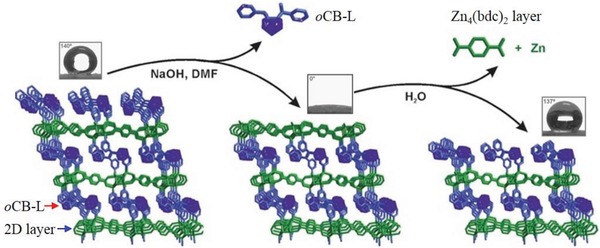
The proposed mechanism for the switchable surface hydrophobicity–hydrophilicity of oCB‐MOF‐1. Pillaring oCB‐L linkers are represented in blue, and Zn_4_(bdc)_2_ layers in green. Inset: water contact angles for the oCB‐MOF‐1 samples. Adapted with permission.^147^ Copyright 2016, John Wiley and Sons.

Rodríguez‐Hermida et al. reported a unique pillared‐layer MOF, [Zn_4_O_2_(1,4‐bdc)_2_(oCB‐L)_2_(DMF)_2_] (*o*CB‐MOF‐1),^147^ which was synthesized by mixed ligands of 1,4‐H_2_bdc and a neutral hydrophobic carborane‐based linker, oCB‐L. The 3D framework of *o*CB‐MOF‐1 consists of square‐grid‐like Zn_4_(1,4‐bdc)_2_ layers, which are pillared by the neutral oCB‐L ligands. There are 1D channels with small apertures (3.2 × 6.4 Å) and larger cavities with a diameter of 8.6 Å (**Figure** 14), and the carborane moieties of oCB‐L ligands are located on the pore surface. The N_2_ adsorption measurement at 77 K for *o*CB‐MOF‐1 showed no uptake of N_2_ at 77 K; however, evident CO_2_ adsorption at 195 K with a type I isotherm and a saturated uptake of 69.4 cm^3^ g^−1^ was noted. The BET surface area of *o*CB‐MOF‐1 was estimated to be 296 m^2^ g^−1^. The authors explained that the small pore aperture in *o*CB‐MOF‐1 should be responsible the adsorption selectivity of CO_2_ over N_2_. It was found that oCB‐MOF‐1 was stable in aqueous solutions with a wide range of pH (2–12) for at least 15 h at room temperature. The high stability was attributed to the high hydrophobicity of oCB‐MOF‐1, which was suggested by the water adsorption and water contact angle measurements. A type III water adsorption isotherm was observed for oCB‐MOF‐1, and the water uptake at 0.95 *P*/*P*_0_ and 298 K was 50 mg g^−1^. The water contact angles for a hand‐packed powder sample on a glass surface and for a pellet sample (formed under a pressure of 10 tons for 5 min) with a roughness factor of 1.7 were 140° and 108°, respectively. Interestingly, a reversible transformation between hydrophobic and superhydrophilic (water contact angle: 0°) surfaces was found when oCB‐MOF‐1 was immersed in a solution of NaOH in DMF and subsequently in a slightly acidic aqueous solution. By ^1^H‐ and ^11^B‐{^1^H}‐NMR spectroscopy, mass spectrometry and ICP‐MS analyses, the switching of the surface hydrophobicity/hydrophilicity of the oCB‐MOF‐1 crystals was attributed to the selective removal of hydrophobic oCB‐L linkers from the crystal surface under basic conditions and the selective removal of hydrophilic Zn_4_(bdc)_2_ layers under slightly acidic aqueous conditions (Figure 14). When the layers of hydrophobic oCB‐L linkers were removed, the hydrophilic Zn_4_(bdc)_2_ layers were exposed to the crystal surface, and when the hydrophilic Zn_4_(bdc)_2_ layers were removed, the crystal surface recovered hydrophobicity, as the oCB‐L linkers were exposed on the crystal surface. The authors proposed that this type of material with a switchable hydrophobic/hydrophilic surface would be useful for myriad applications.

**Figure 15:**
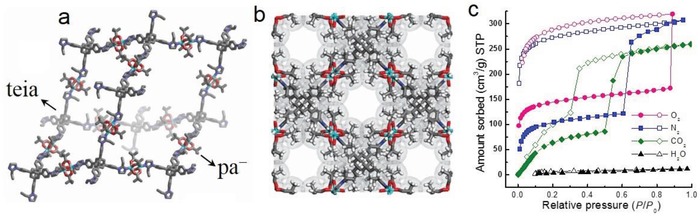
a) A single diamondoid net formed by the interconnection of teia ligands and paddle‐wheel‐shaped [Cu_2_(Me_3_CCOO)_4_] units in SNU‐80. b) 1D channels in SNU‐80 formed by the sixfold interpenetrated diamondoid nets, which are lined with the hydrophobic *tert*‐butyl and ethyl groups of the ligands. c) Adsorption isotherms for N_2_ (77 K), O_2_ (77 K), CO_2_ (195 K), and H_2_O (298 K). Filled shapes: adsorption and open shapes: desorption. Adapted with permission.^151^ Copyright 2011, John Wiley and Sons.

Xie and Suh reported a flexible and hydrophobic MOF, [Cu_4_(pa)_8_(teia)] (SNU‐80), which was synthesized by mixed ligands of the monocarboxylic acid Hpa and a tetrahedral shaped neutral ligand, teia.^151^ The MOF is a 3D framework with 6‐fold interpenetrated diamondoid nets, which consist of [Cu_2_(Me_3_CCOO)_4_] paddle‐wheel units interconnected by the tetrahedral teia ligands (**Figure** 15a). There are 1D channels with a diameter of ≈6.5–7.0 Å, the surfaces of which are predominantly covered by the hydrophobic *tert*‐butyl groups of pivalate ligands and by the ethyl groups of teia ligands (Figure 15b). Due to the structural transformation after activation, SNU‐80 showed stepwise adsorptions of N_2_, O_2_, and CO_2_ gases at 77, 77, and 195 K, respectively (Figure 15c). It was believed that SNU‐80 underwent a structural transformation due to pore expansion during the gas adsorption processes. The Langmuir and BET surface areas and the pore volume of the expanded‐pore phase of SNU‐80 were estimated as 1167 m^2^ g^−1^, 1035 m^2^ g^−1^, and 0.43 cm^3^ g^−1^ from the desorption branch of the N_2_ adsorption isotherm at 77 K. Additionally, according to the adsorption branch of the N_2_ adsorption isotherm, the Langmuir and BET surface areas and the pore volume for the shrunken‐pore phase of SNU‐80 were 456 m^2^ g^−1^, 398 m^2^ g^−1^, and 0.18 cm^3^ g^−1^. The highly hydrophobic nature of SNU‐80 was confirmed by the water adsorption experiment, TGA and elemental analysis (EA). The water adsorption isotherm at 298 K showed a low uptake of 12 cm^3^ g^−1^ (9.6 mg g^−1^) at 0.97 *P*/*P*_0_. TGA also confirmed only a 1.0% weight loss at 25–150 °C for adsorbed water after a SNU‐80 sample was exposed at saturated water vapor at room temperature for one week. The EA data of the saturated water vapor sample of SNU‐80 were almost identical to those of a fresh activated sample of SNU‐80. In addition, the PXRD patterns suggested that SNU‐80 was stable in saturated water vapor and liquid water for one week; however, it was also found that the as‐synthesized phase of SNU‐80 (with *n*‐propanol as guest) turned amorphous after being immersed in water for just 6 h and degraded to a colorless powder of teia ligands after 2 days. The authors believed that the structural shrinkage from the as‐synthesized phase to the activated phase of SNU‐80 after guest removal enhanced the hydrophobicity of the pore surface and hydrolytic stability of the MOF.

While mixed‐ligand hydrophobic MOFs are mostly constructed by the combination of an anionic ligand and a neutral ligand, there are also examples built from two types of anionic ligands. Chen and co‐workers reported a series of MOFs (MAF‐X10 formulated as [Zn_4_O(tmbpz)_2_(1,4‐bdc)], MAF‐X12 formulated as [Zn_4_O(tmbpz)_2_(1,4‐ndc)], and MAF‐X13 formulated as [Zn_4_O(tmbpz)_2_(bpdc)]) from the mixed ligands of a linear dicarboxylic acid (1,4‐H_2_bdc, 1,4‐H_2_ndc or H_2_bpdc) and a linear bipyrazole (H_2_tmbpz).^157^, ^158^ These MOFs are isostructural to the well‐known MOF‐5, consisting of 6‐connected Zn_4_O clusters bridged by linear ligands. Their BET surface areas range from 1787 to 2742 m^2^ g^−1^. The MOFs were regarded as hydrophobic, because their channel surfaces are lined with hydrophobic methyl groups and aromatic rings.

With some similar structural characteristics to the MOFs built from mixed ligands, some hydrophobic MOFs were synthesized by ligands with two types of coordination donors, mostly O and N atoms. Montoro et al. reported a MOF‐5‐type MOF, [Zn_4_O(dmpc)_3_] (Zn‐dmpc), created by using a mixed pyrazolate/carboxylate ligand H_2_dmpc.^155^ Zn‐dmpc showed smaller channels (4–6 Å) and a lower porosity (BET surface area: 840 m^2^ g^−1^), but a higher stability than those of MOF‐5. PXRD patterns indicated Zn‐dmpc remained crystalline after being suspended in water at room temperature for 24 h. The high hydrophobicity of Zn‐dmpc was confirmed by water adsorption measurements at 298 K, showing a type V isotherm with low uptakes below 0.7 *P*/*P*_0_ and gradual pore filling at higher partial pressures (437 mg g^−1^ at 0.83 *P*/*P*_0_). Reproducible water adsorption isotherms for two cycles were observed, further supporting the high water stability of Zn‐dmpc. He et al. obtained a pillared‐column type MOF, [Zn(mpba)] (MAF‐X8), by using a mixed pyrazolate/carboxylate ligand, H_2_mpba (an expanded version of H_2_dmpc),^156^ which showed large 1D channels in a size of 6.7–8.8 Å, and high BET (1161 m^2^ g^−1^) and Langmuir surface areas (1306 m^2^ g^−1^). The pores of MAF‐X8 were believed to be hydrophobic because the methyl groups of the ligand on the pore surface almost entirely blocked the Zn^2+^ ions and partially blocked the carboxylate O atoms.

#### 3.1.4 Phosphonate Monoesters/Phosphonic Acid Monoesters

**Scheme 4:**
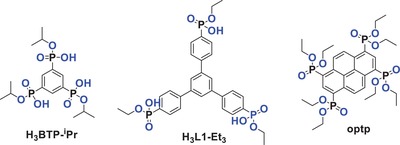
Phosphonate monoester/phosphonic acid monoester ligands for constructing hydrophobic MOFs.

Phosphonate monoesters (RPO_2_OR′^−^) and deprotonated phosphonic acid monoesters (H_2_RPO_2_OR′) are a type of ionic ligands offering O donors. Unlike carboxylates or azolates, phosphonate monoesters are less explored as ligands for the construction of MOFs. Three phosphonate monoester/phosphonic acid monoester ligands used for constructing hydrophobic MOFs reported in the literature are shown in **Scheme** 4.

**Figure 16:**
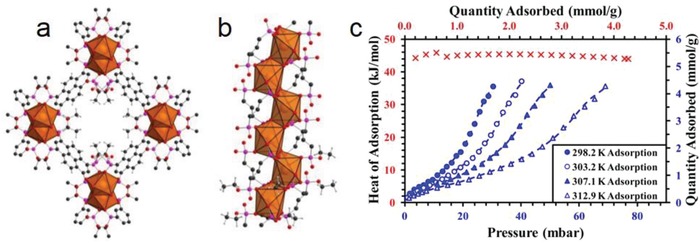
a) Framework structure of CALF‐25 with ethyl‐lined 1D channels. b) 1D barium phosphonate chain. c) Water adsorption isotherms (blue) and the isosteric heat of water adsorption (red) for CALF‐25. Adapted with permission.^85^ Copyright 2012, American Chemical Society.

Shimizu's group reported a series of MOFs derived from phosphonate monoesters, some of which have been proven as hydrophobic.^85^, ^159^, ^160^, ^187^, ^188^ In 2012, they reported a barium tetraethyl‐1,3,6,8‐pyrenetetraphosphonate MOF, CALF‐25, from the solvothermal reaction of BaBr_2_ and the ligand optp, which turned into a phosphonate monoester by in situ partial hydrolysis.^85^ CALF‐25 is a 3D framework structure consisting of 1D barium phosphonate chains crosslinked with four neighboring chains by the ligands. There are 1D channels of size 4.59 × 3.89 Å with the ethyl ester groups lining the corners of the pores and with the pyrene backbone of ligands defining their walls (**Figure** 16a,b). N_2_ adsorption at 77 K revealed the moderate porosity of CALF‐25, and the BET surface area was estimated to be 385 m^2^ g^−1^. It was demonstrated by PXRD patterns and N_2_ adsorption measurements that CALF‐25 remained crystalline and porous after exposure to harsh humid conditions (90% relative humidity at 353 K). Water adsorption isotherms were recorded at 5 K intervals between 298 and 313 K and showed type III isotherms with an uptake of 4.27 mmol g^−1^ (76.9 mg g^−1^) at 0.97 *P*/*P*_0_ (298 K) (Figure 16c). The heat of water adsorption was calculated to be ≈45 kJ mol^−1^ for the whole loading range (Figure 16c), slightly higher than the heat of vaporization of water and comparable to those of graphite and other hydrophobic MOFs, indicting the hydrophobic pore surface of CALF‐25. The authors believed that the ethyl groups of the ligands on the pore surface shielded the polar barium phosphonate chains and resulted in the hydrophobicity of CALF‐25.

Shimizu and co‐workers later reported another copper(II) phosphonate monoester CALF‐30, formulated as [Cu_3_(btp‐^i^Pr)_2_], obtained by using the phosphonic acid monoester ligand H_3_btp‐^i^Pr.^159^ CALF‐30 was synthesized as a microcrystalline blue powder, and it was proposed to be a 2D layered structure possessing small hexagonal pores of 3.57 Å that were defined by isopropyl ester groups. The gas adsorption studies for CALF‐30 revealed no essential uptakes for N_2_ (77 K), H_2_ (77 K), Ar (195 K), and CH_4_ (195 and 273 K), but an uptake of ≈55 cm^3^ g^−1^ for CO_2_ at 195 K. The BET and Langmuir surface areas were estimated to be 244 and 312 m^2^ g^−1^, respectively, close to the calculated value (352 m^2^ g^−1^) from its model structure. PXRD patterns and CO_2_ adsorption measurements suggested that CALF‐30 was stable after a treatment at 90% RH and 353 K for 24 h, although it dissolves in water. It was thus proposed that CALF‐30 showed a kinetic stability to water, rather than a thermodynamic stability, and the kinetic stability originated from the steric blocking of copper sites by the isopropyl esters of the ligands. Recently, the same group reported another phosphonate monoester derived copper(II) MOF CALF‐33‐Et_3_, formulated as [Cu_3_(L1‐Et_3_)_2_], obtained by the solvothermal reaction of Cu(NO_3_)_2_ and the H_3_L1‐Et_3_ ligand.^160^ By controlling the reaction conditions, an isomorphous MOF of CALF‐33‐Et_3_, CALF‐33‐Et_2_H, was also obtained, where one of the phosphonate monoesters of the ligand was in situ hydrolyzed to hydrogen phosphonate. CALF‐33‐Et_3_ and CALF‐33‐Et_2_H are 3D frameworks consisting of 1D copper–phosphonate chains crosslinked by the ligands, and 1D channels with a size of 7.2 × 16.1 Å were formed along the copper–phosphonate chains. Their BET/Langmuir surface areas were estimated to be 842/1030 and 810/950 m^2^ g^−1^, respectively. Due to the pore surface being lined with ethyl ester groups, the pores of CALF‐33‐Et_3_ were believed to be hydrophobic, as indicated by the low heats of CO_2_ adsorption (16.8–20.8 kJ mol^−1^). The heats of CO_2_ adsorption for CALF‐33‐Et_2_H were higher (20.8–29.6 kJ mol^−1^) than those for CALF‐33‐Et_3_, as a result of the hydrolysis of one of the phosphonate monoesters into hydrogen phosphonate being on its pore surface.

The above‐mentioned examples of hydrophobic MOFs are mostly constructed from the various ligands with hydrophobic moieties. When the pore surface is predominately lined with the hydrophobic moieties, the MOF shows a low affinity to water molecules, and thus exhibits an interior hydrophobicity. One direct consequence is that the hydrolytic stability of the MOF can be improved. In some cases, the MOF shows negligible water uptakes even near saturated partial pressure. Such a highly hydrophobic MOF would be promising in some applications where the water adsorption of adsorbents must be avoided. In addition, the onset pressure for pore filling of water adsorption for a hydrophobic MOF can be fine‐tuned by the modification of organic ligands. This appealing attribution of MOFs should be of high significance for water adsorption‐based applications.

### 3.2 Postsynthetic Hydrophobization

In fact, most reported MOFs are not hydrophobic, and many hydrophilic MOFs are promising adsorbents because of their advantages including facile synthesis, large surface area, high stability, and/or potential for application. A general, facile, and effective approach to impart hydrophobicity to these hydrophilic MOFs is highly desirable. The postsynthetic modification (PSM) of MOFs has been greatly developed over the past decade.^12^, ^189^, ^190^ Among those studies, the postsynthetic hydrophobization of MOFs has received considerable attention. The reported methods for the postsynthetic hydrophobization of MOFs can be roughly divided into two categories: internal surface hydrophobization and external surface hydrophobization. The internal surface refers to the pore surface of MOFs, which can be examined by the crystal structure. The external surface refers to the outer surface of MOF crystals or crystallites, and the property of the external surface of MOFs is normally judged by water contact angle measurement. It should be noted that a high exterior hydrophobicity is not indicative of a high interior hydrophobicity for porous solids, and vice versa.^88^

#### 3.2.1 Internal Surface Hydrophobization

Mostly, the postsynthetic hydrophobization of the internal surface of MOFs is accomplished by a PSM organic reaction of the ligands. As early as in 2008, Cohen and coworkers reported the postsynthetic hydrophobization of two amine‐tagged MOFs, IRMOF‐3 (formulated as [Zn_4_O(1,4‐bdc‐NH_2_)_3_]) and MIL‐53(Al)‐NH_2_ (formulated as [Al(OH)(1,4‐bdc‐NH_2_)]), by the introduction of hydrophobic long alkyl groups or bulky alkyl groups onto their pore surface via PSM reactions of the amine groups with alkyl anhydrides (forming amide substituents).^191^, ^192^ IRMOF‐3 was hydrophilic, absorbing water droplets and displaying a water contact angle of 0°. After long or bulky alkyl groups were introduced by PSM reactions, the resultant MOFs, IRMOF‐3‐AM4 (AM4 = amide with a 4‐carbon chain), IRMOF‐3‐AM5, IRMOF‐3‐AM6, IRMOF‐3‐AM15, IRMOF‐3‐AM*i*Pr (AM*i*Pr = amide with isopropyl group), and IRMOF‐3‐AM*i*Bu (AM*i*Pr = amide with isobutyl group), showed water contact angles of 105°–125°, indicating an enhancement in the external surface hydrophobicity. In contrast, the water contact angles of the MOFs with short alkyl chains (IRMOF‐3‐AM1, IRMOF‐3‐AM2, and IRMOF‐3‐AM3) were still 0°; however, the conversions of IRMOF‐3 to the corresponding amide frameworks with short alkyl chains were high (≈99%), and those with long or bulky alkyl groups were low (25–99%), as estimated by the ^1^H NMR spectra of digested MOFs. The high contact angles of PSM‐resultant MOFs were supported by the observation that IRMOF‐3‐AM6 and IRMOF‐3‐AM15 could float on water, but IRMOF‐3 could not. PXRD patterns and SEM images suggested improved stabilities of the hydrophobic MOFs in open air or in liquid water. Similarly, a water contact angle of 0° was observed for MIL‐53(Al)‐NH_2_ and MIL‐53(Al)‐AM1, but MIL‐53(Al)‐AM4 and MIL‐53(Al)‐AM6 showed water contact angles >150°, suggesting their superhydrophobicity. The superhydrophobicity of the MIL‐53(Al)‐NH_2_‐derived phases was attributed to the combined effect of the hydrophobic functional groups and their submicrometer crystallite size.

Utilizing the facile amide formation reaction of amine groups of ligands with alkyl anhydrides, Rubin and Reynolds investigated the hydrophobicity and stability of postsynthesis modified samples of [Cu_3_(NH_2_BTC)_2_].^193^ Although only low conversions of PSM were achieved, some Cu_3_(NH_2_BTC)_2_ samples grafted with long alkyl chains showed high water contact angles. For example, Cu_3_(NH‐AM10‐BTC)_2_ (AM10 = amide with a 10‐carbon chain) showed a water contact angle of 147°, although, the PSM conversion was only 13%. Interestingly, the water contact angle increased as the number of carbons in the PSM grafted linear alkyl chains increased, and a linear relationship was observed between the number of carbons and the water contact angle. As observed for Cu_3_(BTC)_2_,^194^ Cu_3_(NH_2_BTC)_2_ degraded after water submersion for 30 min. In contrast, PXRD patterns showed that Cu_3_(NH‐AM10‐BTC)_2_ remained crystalline, but the intensity of the PXRD peaks decreased after the same treatment. The difference in water stabilities of the unmodified and modified samples of Cu_3_(NH_2_BTC)_2_ was also supported by SEM images of their surface characteristics before and after water submersion. Wittmann et al. reported that the PSM of the mesoporous MOF MIL‐101(Al)‐NH_2_, formulated as [Al_3_O(H_2_O)_2_F(1,4‐bdc‐NH_2_)_3_], obtained by the reaction of the amine groups of ligands with phenyl isocyanate, forming the phenylurea‐group‐functionalized MOF MIL‐101(Al)‐URPh (URPh = phenylurea), with a conversion of 86%.^195^ Due to the shielding effect of the hydrophobic phenylurea groups, MIL‐101(Al)‐URPh showed a decreased affinity to water compared to that of MIL‐101(Al)‐NH_2_, as indicated by their water adsorption isotherms at 298 K. The authors also found that MIL‐101‐URPh remained stable in liquid water after seven days, but MIL‐101(Al)‐NH_2_ transformed to the thermodynamically more stable MIL‐53(Al)‐NH_2_ after being suspended in water for 5 min.

The PSM of MOFs via the coordination of metal centers with new ligands (so‐called dative PSM^12^) has also been applied to the construction of hydrophobic MOFs. Bae et al. reported the dative PSM of Ni‐MOF‐74 (formulated as [Ni_2_(dobdc)], also named as CPO‐27‐Ni, NiDOBDC or Ni‐DOBDC) with pyridine molecules, which made the hydrophilic internal surface of Ni‐MOF‐74 more hydrophobic.^196^ The BET surface area of a pristine Ni‐MOF‐74 sample was 798 m^2^ g^−1^, which was significantly lower than the theoretical value calculated for the perfect crystal structure of Ni‐MOF‐74 (1182 m^2^ g^−1^) but close to the calculated value (796 m^2^ g^−1^) for the hydrated Ni‐MOF‐74 (hy‐Ni‐MOF‐74) in which all of the open metal sites are occupied by H_2_O molecules. After the PSM with pyridine, the modified MOF showed a surface area of 409 m^2^ g^−1^ and a pore volume of 0.18 cm^3^ g^−1^. The researchers proposed that in this modified MOF, 33% of open metal sites were occupied by pyridine molecules, and the others were occupied by H_2_O molecules because such a structure (Py‐c‐hy‐Ni‐MOF‐74) shows a similar surface area (433 m^2^ g^−1^) and pore volume (0.23 cm^3^ g^−1^). It was pointed out that a structure model of Ni‐MOF‐74 with all open metal sites occupied by pyridine molecules showed no accessible pore space by using nitrogen as a probe. The water adsorption isotherms at 298 K showed substantially less uptake of pyridine modified Ni‐MOF‐74 than did the pristine Ni‐MOF‐74. The authors believed that such a dramatic decrease in H_2_O uptake resulted from the increased hydrophobicity of the modified Ni‐MOF‐74 due to the presence of pyridine groups, rather than a complete contribution from the reductions of surface area and pore volume.

Drache et al. reported the PSM of an 8‐connected (reo topology) Zr(IV)‐based MOF, [Zr_6_O_6_(OH)_2_(tdc)_4_(CH_3_COO)_2_] (DUT‐67),^197^ produced by exchanging coordinated monocarboxylate ligands (formate, fa^−^; acetate, ac^−^; or propionate, pa^−^) of DUT‐67 with fluorinated monocarboxylates, including trifluoroacetate (tfa^−^), 4‐(trifluoromethyl)benzoate (tfmba^−^), pentafluorobenzoate (pfba^−^), and perfluorooctanoate (pfoa^−^), in DMF solutions of the respective carboxylic acid for 5 days. A conversion of 87% was confirmed by ^1^H NMR spectra. The water adsorption isotherms for the DUT‐67 samples before and after the PSM revealed that the exchanged DUT‐67 samples showed decreased water uptakes, especially DUT‐67‐tfmba, DUT‐67‐pfba, and DUT‐67‐pfoa, suggesting that the internal hydrophobicity of DUT‐67 could be tuned by the PSM with these hydrophobic fluorinated monocarboxylates. It was also found that the PSM‐exchanged DUT‐67 samples showed enhanced water stabilities. The pristine DUT‐67 samples lost 20–34% porosity after water adsorption. In contrast, only 0.3–13% loss of porosity was observed for the PSM‐exchanged DUT‐67 samples after water adsorption. In addition, the water contact angle measurements revealed a very hydrophilic external surface for DUT‐67‐fa, which completely absorbed the water droplet. The water contact angles of DUT‐67‐ac, DUT‐67‐pa, and DUT‐67‐tfa were similar, close to 60°, but DUT‐67‐pfba and DUT‐67‐pfoa were regarded as hydrophobic materials due to their large water contact angles (103° and 119°).

**Figure 17:**
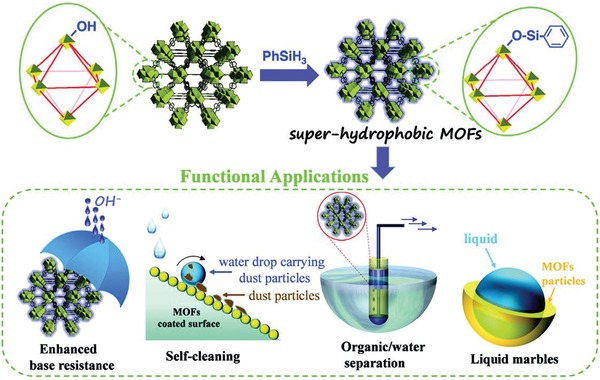
Schematic representation of the preparation of UiO‐66‐NH_2_‐shp and its potential applications. Adapted with permission.^198^ Copyright 2019, John Wiley and Sons.

Instead of using the coordination bonding between open metal sites and hydrophobic terminal ligands, covalent bonding between inorganic SBUs and reactive species has also been applied to the hydrophobization of MOFs. Recently, Sun et al. reported the hydrophobization of UiO‐66‐NH_2_ (formulated as [Zr_6_O_4_(OH)_4_(1,4‐bdc‐NH_2_)_6_]) by the reaction of phenylsilane (PhSiH_3_) with the hydroxyl groups on its Zr_6_O_4_(OH)_4_ clusters (**Figure** 17).^198^ While the pristine UiO‐66‐NH_2_ showed a water contact angle of 0°, in contrast, a silylated sample of UiO‐66‐NH_2_ (UiO‐66‐NH_2_‐shp) showed a very high water contact angle of 161°, although, its BET surface area decreased from 974.4 m^2^ g^−1^ to 656.5 m^2^ g^−1^. According to the analyses of ^1^H NMR and GC‐MS spectra, it was confirmed that the reaction occurred between phenylsilane and the hydroxyl groups of Zr_6_O_4_(OH)_4_ clusters rather than the —NH_2_ group of the ligand. UiO‐66‐NH_2_‐shp showed an enhanced stability under a basic condition (0.1 m NaOH) and great potential in toluene/water separation, self‐cleaning, and fabricating magnetic liquid marbles (by rolling a water droplet containing Fe_3_O_4_ over the powder of the superhydrophobic UiO‐66‐NH_2_‐shp).

Decoste et al. reported the PSM of HKUST‐1 by a plasma‐enhanced chemical vapor deposition (PECVD) of perfluorohexane.^199^ PXRD patterns showed that the PECVD‐treated sample of HKUST‐1 (referred to as HKUST‐1 Plasma) matched that of pristine HKUST‐1, but a decrease in the intensity of the PXRD peaks was observed. This result was attributed to the pore filling by perfluorohexane or nonperiodic minor structural changes in the HKUST‐1 framework. HKUST‐1 Plasma showed an enhanced water stability, and it could float on water, indicative of its external surface hydrophobicity. In addition, HKUST‐1 degraded after immersion in water at room temperature for 24, but HKUST‐1 Plasma retained its structure under the same treatment. The improvement in water stability was further evidenced by SEM images of HKUST‐1 and HKUST‐1 Plasma before and after exposure to 90% humidity, where the cracking of crystal surfaces and the loss of regular crystal morphology were only observed for HKUST‐1. The ^19^F magic‐angle spinning (MAS) nuclear magnetic resonance (NMR) spectra indicated the presence of perfluorohexane and minor other unknown perfluoro products in HKUST‐1 Plasma, but no evidence of polymerization of perfluorohexane. The authors believed that the unknown perfluoro products were from the diffusion of reactive CF_3_ species across the plasma−solid interface, and the formation of the unknown perfluoro products on the pore surface of HKUST‐1 prompted the further loading of perfluorohexane (16.1 wt%) because no loading of fluorinated species was found in a HKUST‐1 sample after it was directly exposed to liquid or gaseous perfluorohexane and subsequently treated by light heating (note: from TGA, the desorption of perfluorohexane from HKUST‐1 Plasma only occurred at temperatures above 175 °C). Interestingly, HKUST‐1 Plasma showed higher NH_3_ uptakes (8.7 mmol g^−1^) than HKUST‐1 (6.4 mmol g^−1^) under dry conditions and wet conditions (80% RH, 11.8 vs 10.4 mmol g^−1^), although a decrease in the BET surface area (25.9%) and in the total pore volume (31.7%) was observed after the PSM. The enhancement in NH_3_ adsorption capacity of HKUST‐1 Plasma was explained by an improved stability in the presence of NH_3_ and/or water.

**Figure 18:**
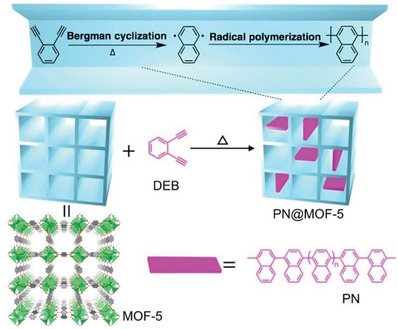
Illustration of the polymerization of DEB in MOF‐5, giving the composite PN@MOF‐5. Adapted with permission.^200^ Copyright 2016, American Chemical Society.

Ding et al. reported the PSM of water‐sensitive MOF‐5 by the polymerization of 1,2‐diethynylbenzene (DEB) in its channels.^200^ The monomer DEB was first adsorbed in MOF‐5, which was followed by Bergman cyclization and a subsequent radical polymerization at an elevated temperature, producing PN@MOF‐5 with polynaphthylene (PN) inside its channels (**Figure** 18). A low BET surface area (1200 m^2^ g^−1^) and small pores (6 Å) were observed for PN@MOF‐5 compared to those of the pristine MOF‐5 (3200 m^2^ g^−1^ and 12 Å). The PXRD patterns and N_2_ adsorption experiments (77 K) revealed that PN@MOF‐5 retained its crystallinity and porosity upon exposure to humid air (RH = 40%) for over 40 h; however, the pristine MOF‐5 completely loses its porosity and transformed into MOF‐69c after the same treatment.^201^ PN@MOF‐5 also showed a hydrophobic external crystal surface, as confirmed by its large water contact angle (135°). In contrast, the pristine MOF‐5 showed a water contact angle of 0°. From the pore size distribution analysis, PXRD measurements, and microscopy images, the authors concluded that most of the PN polymer is formed inside the crystal channels rather than on the external crystal surface, serving as partitions to segregate the channels of the crystals into confined compartments. It was also demonstrated that the postsynthetic hydrophobization method via the polymerization of aromatic acetylenes in MOF channels also worked for another [Zn_4_O]^6+^‐cluster‐based MOF, UMCM‐8.^202^

#### 3.2.2 External Surface Hydrophobization

The hydrophobization of MOFs by the modification of their internal surface with hydrophobic species is straightforward. On the other hand, diverse approaches have been developed to only modify the external surface of MOFs, which also effectively enhances their hydrophobicity, stability, and/or other physical and chemical properties. The materials that have been applied to modify the external surface of MOFs include hydrophobic small molecules/ligands, inorganic materials (e.g., amorphous carbon and silica), and organic polymers (e.g., polydimethylsiloxane, PDMS and PVDF).

**Figure 19:**
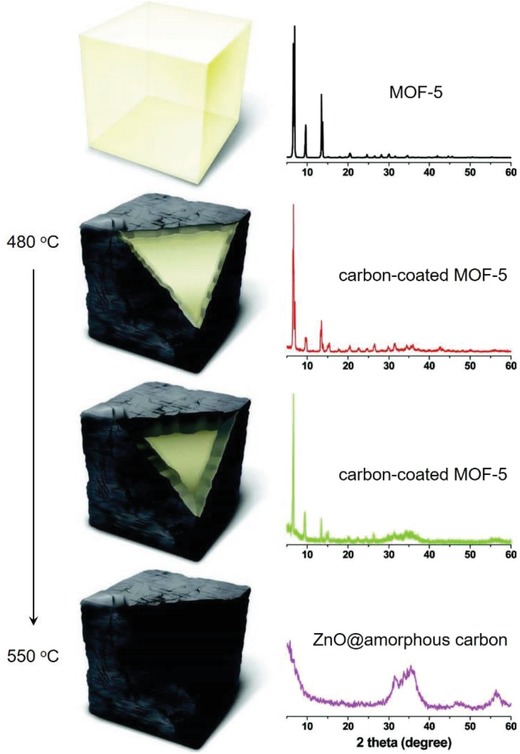
Schematic representations of MOF‐5, amorphous‐carbon‐coated MOF‐5 samples, and ZnO nanoparticles@amorphous carbon prepared by thermal modification at different temperatures. Adapted with permission.^203^ Copyright 2012, John Wiley and Sons.

Yang and Park reported that an amorphous‐carbon‐coated MOF‐5 could be prepared by a simple thermal modification under a N_2_ atmosphere in a temperature range of 480–530 °C.^203^ Whereas higher temperatures produced MOF‐5 samples with thicker carbon coatings, and overheating at 550 °C resulted in the complete transformation of MOF‐5 into a ZnO@amorphous carbon composite (**Figure** 19). It was demonstrated that the amorphous‐carbon‐coated MOF‐5 samples showed lower BET surface areas (1740 vs 3450 m^2^ g^−1^) but higher water stabilities than pristine MOF‐5. The PXRD patterns showed that MOF‐5 initiated a phase transformation after exposure in air for 3 days, but the carbon‐coated MOF‐5 samples showed no new PXRD peaks or reduction in peak intensity after exposure in air for 14 days. After the air exposure for 14 days, the BET surface area of MOF‐5 was reduced from 3450 to 960 m^2^ g^−1^, but only a decrease of 1% was noted in the BET surface area (1740 to 1720 m^2^ g^−1^) was observed for a 530 °C thermally modified MOF‐5 sample after the same treatment. The carbon‐coated MOF‐5 even retained its structure after it was soaked in liquid water for 2 h; however, the well‐faceted cubic shaped crystals of MOF‐5 rapidly turned to a powder sample after being immersed in water for 10 seconds, and the crystal structure was completely changed after the water treatment. All the findings indicated that a hydrophobic amorphous carbon coating on the MOF‐5 crystal surface could prevent the MOF from hydrolysis by water to some extent. Ying et al. reported the coating of MIL‐101(Cr) with a thin layer (≈30 nm) of hydrophobic mesoporous silica (mSiO_2_).^204^ The core−shell‐structured MIL‐101(Cr)@mSiO_2_ nanoparticles showed an enhanced hydrophobicity compared with that of MIL‐101(Cr).

**Figure 20:**
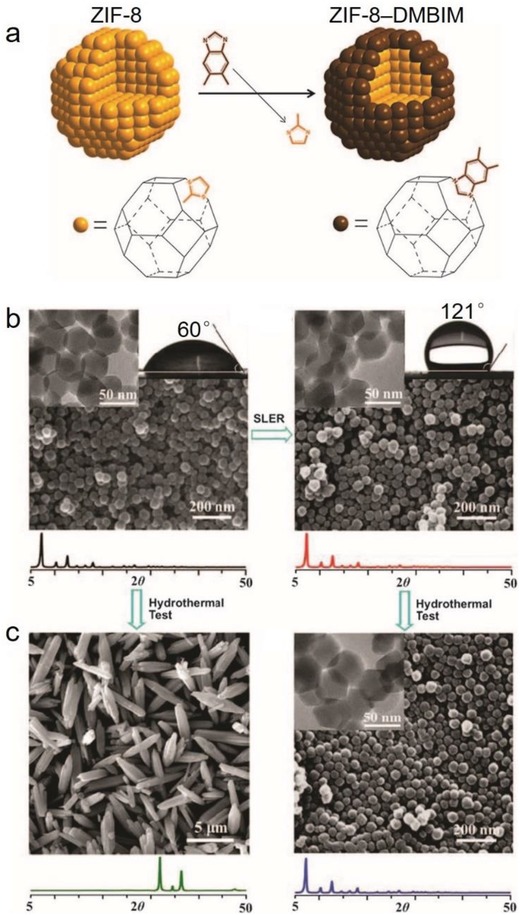
a) Schematic representation of the surface ligand exchange reaction from ZIF‐8 to ZIF‐8–DMBIM. SEM images, PXRD patterns, and water contact angles for ZIF‐8 (left) and ZIF‐8–DMBIM (right) b) before and c) after being treated with water at 80 °C for 24 h. Adapted with permission.^176^ Copyright 2013, Royal Society of Chemistry.

Liu et al. reported the postsynthetic hydrophobization of ZIF‐8 by a shell‐ligand exchange reaction of the external surface of crystals.^176^ The authors observed that ZIF‐8 underwent hydrolysis in excessive water, although this material was commonly regarded as a highly stable MOF. A ligand exchange reaction was carried out to replace the 2‐methylimidazolate ligands on the outermost shell of ZIF‐8 particles with a more hydrophobic ligand, 5,6‐dimethylbenzimidazolate (DMBIM^−^), producing the modified phase, referred to as ZIF‐8–DMBIM (**Figure** 20a). ZIF‐8–DMBIM showed a hydrophobic surface and enhanced water stability. The success of the ligand exchange reaction was confirmed by FTIR‐ATR, UV‐vis and Raman spectroscopies, as well as water contact angle measurements, which revealed the enhanced hydrophobicity of the external surface of ZIF‐8–DMBIM (water contact angle: 121°) compared to that of ZIF‐8 (water contact angle: 60°). From the N_2_ adsorption studies, ZIF‐8–DMBIM showed an only slightly lower BET surface area (1346 m^2^ g^−1^) and pore volume (0.567 cm^3^ g^−1^) than those of ZIF‐8 (1360 m^2^ g^−1^ and 0.572 cm^3^ g^−1^), suggesting that the ligand exchange reaction occurred only at the sample surface. After being treated with water at 80 °C for 24 h, ZIF‐8 transformed into ZnO, but the crystalline structure and morphology of ZIF‐8–DMBIM remained unchanged, as indicated by PXRD patterns and SEM images (Figure 20b,c). The enhanced hydrophobicity and hydrolytic stability of ZIF‐8–DMBIM was attributed to a water‐repellent effect and the steric hindrance effect of DMBIM ligands.

Sanil et al. reported that the external surface of HKUST‐1 could be functionalized by aminopropylisooctyl polyhedral oligomeric silsesquioxane (O‐POSS), a hybrid molecule consisting of cubic octameric silica cages with 8 hydrophobic isooctyl groups at the cube corners.^205^ The coating of HKUST‐1 by O‐POSS was carried out in refluxing hexane under nitrogen for 48 h by a driving force from the coordination between the open metal sites of HKUST‐1 and the amine groups of O‐POSS. It was demonstrated that the O‐POSS‐modified HKUST‐1 showed a larger water contact angle (83° vs 46°) and improved water stability than did the pristine HKUST‐1. The postsynthetic external surface hydrophobization method with O‐POSS was also proven valid for two other MOFS containing open metal sites, M‐MOF‐74 (M = Ni or Co) and MIL‐100.

**Figure 21:**
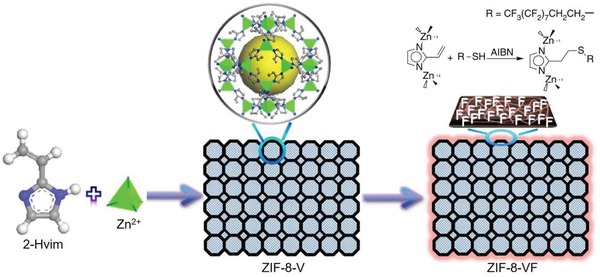
Schematic illustration of the postsynthetic modification of ZIF‐8‐V by a thiol‐ene click reaction with 1*H*,1*H*,2*H*,2*H*‐perfluorodecanethiol, resulting in the amphiphobic ZIF‐8‐VF. Adapted with permission.^206^ Copyright 2016, Springer Nature.

Sun et al. reported that a vinyl‐functionalized MOF [Zn(2‐vim)_2_] (ZIF‐8‐V), isostructural to ZIF‐8 and synthesized by the ligand 2‐Hvim, could be surface‐modified by a thiol‐ene click reaction with 1*H*,1*H*,2*H*,2*H*‐perfluorodecanethiol, affording ZIF‐8‐VF (**Figure** 21).^206^ ^] 13^C and ^19^F MAS NMR spectra revealed that only 2% of the vinyl groups in the 2‐Hvim ligands were grafted with perfluoroalkyl groups after the postsynthetic modification, suggesting the thiol‐ene click reaction occurred only on the exterior surface of ZIF‐8‐V. ZIF‐8‐VF showed a very large water contact angle (173°), considerably higher than that of ZIF‐8‐V (89°). The improved surface hydrophobicity should be a result of the grafting of perfluoroalkyl groups on the MOF crystal surface. The researchers also found that ZIF‐8‐VF showed high contact angles for some organic compounds, including glycerol (150°), 2‐hydroxybenzaldehyde (143°), benzonitrile (130°), chlorobenzene (129°), and dodecane (92°), indicating the amphiphobic nature of ZIF‐8‐VF. The amphiphobicity of ZIF‐8‐VF was further confirmed by water and toluene adsorption studies at 298 K, which showed lower uptakes (negligible for water and 120 mg g^−1^ for toluene at 0.9 *P*/*P*_0_) than those of ZIF‐8‐V (20 and 240 mg g^−1^), even at near‐saturated vapor pressures. After being exposed to saturated water vapor in a CO_2_ atmosphere at 45 °C for 10 days, ZIF‐8 and ZIF‐8‐V both showed structural degradation, but ZIF‐8‐VF retained well its crystalline structure and porosity, as indicated by PXRD patterns, SEM images and N_2_ adsorption isotherms.

**Figure 22:**
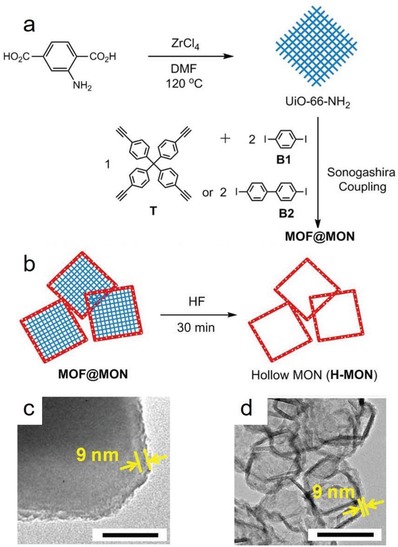
Synthesis schemes for a) MOF@MON and b) hollow MONs (H‐MONs). TEM images of c) MOF@MON‐4 and d) H‐MON‐4. Adapted with permission.^207^ Copyright 2014, American Chemical Society.

Chun et al. reported the coating of a highly stable MOF, UiO‐66‐NH_2_, with microporous organic networks (MONs), resulting in hybrid microporous materials with hydrophobic external surfaces.^207^ The coating of UiO‐66‐NH_2_ with MONs was carried out by dispersing UiO‐66‐NH_2_ powders in a reaction mixture for the Sonogashira coupling of the MON monomers. Three samples, MOF@MON‐1, MOF@MON‐2, and MOF@MON‐3, were obtained from the coupling of tetra(4‐ethynylphenyl)methane (10, 20, and 30 mg, respectively) with 2 equiv of 1,4‐diiodobenzene and 100 mg UiO‐66‐NH_2_, and MOF@MON‐4 was obtained when 1,4‐diiodobenzene was replaced by the longer monomer 4,4′‐diiodobiphenyl (**Figure** 22a). SEM and transmission electron microscopy (TEM) images showed that the MON layers on UiO‐66‐NH_2_ were 8–30 nm for the four MOF@MONs (Figure 22c). The MON layers were distinguished by a contrast that was lighter than that of UiO‐66‐NH_2_ in the TEM images. The authors also dissolved UiO‐66‐NH_2_ inside the MOF@MONs by an HF solution, to confirm the core‐shell structures of the MOF@MONs, and imaged the resultant hollow MONs, referred as to H‐MON‐1, H‐MON‐2, H‐MON‐3, and H‐MON‐4 (Figure 22b,d). The PXRD patterns revealed that the crystal structure of UiO‐66‐NH_2_ was retained in MOF@MONs and that H‐MONs were amorphous. The N_2_ sorption isotherms at 77 K showed a decrease in the BET surface area for MOF@MONs (703–809 m^2^ g^−1^) compared to that for UiO‐66‐NH_2_ (1070 m^2^ g^−1^) and H‐MONs (866–1138 m^2^ g^−1^), and this decrease was attributed to the partial pore occupancy of UiO‐66‐NH_2_ by MONs. The pellets of MOF@MONs show water contact angles of 121°–145°, indicative of highly hydrophobic external surfaces. In contrast, a water drop was completely adsorbed by UiO‐66‐NH_2_, suggesting its highly hydrophilic external surface. In addition, the researchers observed that UiO‐66‐NH_2_ was well‐immersed in the water, but MOF@MONs could float on water for a week, even when vigorous shaking was applied.

**Figure 23:**
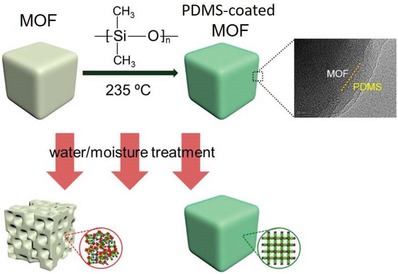
Illustration of PDMS coating process for the surface of MOFs and the improvement in the moisture/water stability of MOFs. Adapted with permission.^208^ Copyright 2014, American Chemical Society.

Some studies have revealed that the postsynthetic hydrophobization of MOFs could be achieved by simply coating their surface with certain commercially available hydrophobic polymers.^208^ Zhang et al. demonstrated a PDMS‐coating approach for three MOFs, MOF‐5, HKUST‐1, and [Zn(1,4‐bdc)(dabco)_0.5_]^209^ (denoted as ZnBT). Typically, the coating process was carried out by heating the MOFs together with a PDMS stamp in a sealed glass container at 235 °C. The authors purported that volatile and low‐molecular‐weight silicone molecules from the thermal degradation of PDMS deposited and crosslinked on the MOF surfaces. A high‐resolution TEM image showed that the thickness of the PDMS layer coated on MOF‐5 was ≈10 nm (**Figure** 23). In contrast to the hydrophilic surfaces of the pristine MOFs, the PDMS‐coated MOFs show hydrophobic external surfaces, as indicated by their water contact angles ranging from 128° to 130°. It was also observed that the PDMS‐coated HKUST‐1 could float on water for three months, indicating the stability of the PDMS coating. Noteworthily, N_2_ adsorption studies of the pristine and PDMS‐coated MOF samples showed that the porosities of the pristine MOFs were essentially unaltered after the PDMS coating, suggesting that the MOF pores were not blocked at all after the deposit of the crosslinked silicone molecules. The PDMS‐coated MOF samples also showed significantly improved water stabilities than those of the pristine ones. For example, after exposure to water for 3 days, the pristine HKUST‐1 turned into a nonporous phase, but the BET surface area of the PDMS‐coated HKUST‐1 remained close to that of the pristine HKUST‐1 (1544 vs 1547 m^2^ g^−1^). Water adsorption isotherms were also recorded for the HKUST‐1 samples at 298 K. The water uptakes of PDMS‐coated HKUST‐1 (511 mg g^−1^ at 0.94 *P*/*P*_0_) were lower than those of the pristine HKUST‐1 (728 mg g^−1^ at 0.94 *P*/*P*_0_) for the entire pressure range. As the water adsorption measurement took a longer time for PDMS‐coated HKUST‐1 (48 h) than for the pristine HKUST‐1 (24 h), the authors concluded that after the PDMS coating, the MOF exhibited slower water adsorption kinetics, which resulted from the difficulty in the diffusion of water molecules through the hydrophobic PDMS coating layer. Qian et al. later reported the postsynthetic hydrophobization of three MOFs, NH_2_‐MIL‐125(Ti),^210^ ZIF‐67,^133^ and HKUST‐1,^211^ with organosilicone (DC 1–2577) by a solution‐immersion process.^212^ The method was believed to be facile because the process was simple and heating was not involved. The solution‐immersion coating process was just mixing the organosilicone DC 1–2577 (≈40 mg) with powder samples of the MOFs (500 mg) in heptane (5 mL) by ultrasonication for 10 min, followed by drying of the mixture under vacuum at room temperature for 12 h. The organosilicone‐coated MOFs showed no blocking of their intrinsic pores or the hydrophobic external surface as indicated by their high water contact angles (≈146°), and an enhanced water stability compared to that of the pristine MOFs was achieved, as indicated by PXRD patterns, SEM images and N_2_ adsorption studies at 77 K for the MOF samples before and after exposure to liquid water for 5 days. Fernandez et al. reported the coating of MIL‐101(Cr) and Ni‐MOF‐74 with a triblock copolymer, Pluronic P123.^213^ The polymer‐modified MOFs showed uncompromised CO_2_ sorption capacities, low water uptakes, and improved water stability compared to the pristine MOFs. DeCoste et al. found that HKUST‐1 in mixed‐matrix membranes prepared from the hydrophobic polymer polyvinylidene difluoride (PVDF) yielded an improved stability upon exposure to humid environments for prolonged periods of time and an uncompromised adsorption capacity for NH_3_.^214^

### 3.3 In Situ Synthetic Hydrophobization

Some hydrophobic MOFs and MOF‐derived materials have been prepared by hydrophobic ligands generated in situ or by introducing new hydrophobic species into the synthetic reactions of previously known MOFs. Zu et al. reported the preparation of HKUST‐1/graphite oxide composites by introducing graphite oxide into a solution containing the starting materials for the synthesis of HKUST‐1 before the mixture was heated at 85 °C for 21 h.^215^ Different amounts of graphite oxide were introduced, providing for different samples denoted as MG‐1, MG‐2, MG‐3, MG‐4, and MG‐5. SEM images revealed that the HKUST‐1/graphite oxide composites consisted of thin platelets stacked together; this outcome was different from both HKUST‐1 (octahedral shaped crystals) and graphite oxide (dense agglomerates of stacked graphene sheets). The authors proposed that the growth of crystalline HKUST‐1 occurred between graphene layers and their thin agglomerates, which were generated from the exfoliation of graphite oxide during the synthesis. Interestingly, the composites showed higher surface areas and pore volumes than did pristine HKUST‐1 except MG‐5, the one with the highest graphite oxide content. MG‐2, with 8.7 wt% graphite oxide introduced, showed the highest surface area (1257 m^2^ g^−1^) and pore volume (0.552 cm^3^ g^−1^), higher than those of HKUST‐1 (841 m^2^ g^−1^ and 0.433 cm^3^ g^−1^). The formation of minor meso/macropores in the composites was also suggested by the small adsorption‐desorption hysteresis loops observed at high relative pressures. It was believed that extra pores were formed between HKUST‐1 and the graphene layers in the composites. The hydrophobicity of the composites was suggested by the water and benzene adsorption isotherms. For HKUST‐1, the water uptake (10.7 mmol g^−1^) was significantly higher than the benzene uptake (3.0 mmol g^−1^) at saturated partial pressures. In contrast, MG‐2 showed a water uptake of 1.2 mmol g^−1^ and a benzene uptake of 5.5 mmol g^−1^. The incorporation of graphite oxide made HKUST‐1 not only hydrophobic but also more stable to water. After being exposed to water vapor at 90 °C for 12 h, the PXRD patterns suggested the structural degradation of HKUST‐1, but no structural change was observed for MG‐2, MG‐3, and MG‐4. Too little and too much graphite oxide was introduced into MG‐1 and MG‐5 and did not efficiently improve their water stability.

**Figure 24:**
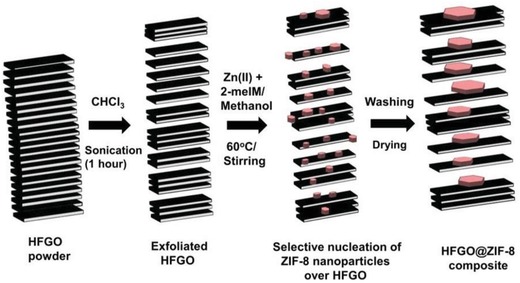
Schematic representation of the synthesis of the HFGO@ZIF‐8 composite. Adapted with permission.^177^ Copyright 2016, John Wiley and Sons.

Jayaramulu reported the preparation of a composite comprising highly fluorinated graphene oxide (HFGO) and ZIF‐8 (denoted as HFGO@ZIF‐8) through a bottom‐up solution‐assisted self‐assembly method.^177^ HFGO was exfoliated before it was introduced into the synthetic reaction mixture for nanoscale ZIF‐8. The authors proposed that exfoliated HFGO nanosheets acted as a support for the nucleation of ZIF‐8 nanoparticles, and the ZIF‐8 nanoparticles (2–25 nm) acted as pillars that were firmly intercalated between single‐/few‐layered HFGO sheets in the HFGO@ZIF‐8 composite (**Figure** 24). Additionally, mesoporosity developed between the HFGO layers due to the presence of ZIF‐8 nanopillars. The proposed structure of HFGO@ZIF‐8 was further supported by its TEM, HAADF‐STEM, and AFM images. From the N_2_ adsorption studies, ZIF‐8, HFGO, and HFGO@ZIF‐8 showed BET surface areas of 1150, 5, and 590 m^2^ g^−1^, respectively. The N_2_ adsorption isotherm of HFGO@ZIF‐8 was a combination of type I and type IV isotherms, indicating the presence of both micropores from ZIF‐8 and mesopores formed by ZIF‐8‐nanocrystal‐pilllared HFGO layers. HFGO@ZIF‐8 possessed a superhydrophobic surface, as indicated by its large water contact angle (162°), that was considerably higher than that of ZIF‐8 (56°) or HFGO (125°). Furthermore, an oil droplet completely penetrated into the HFGO@ZIF‐8 composite in less than 15 seconds (oil contact angle = 0°), indicating its superoleophilic nature.

**Figure 25:**
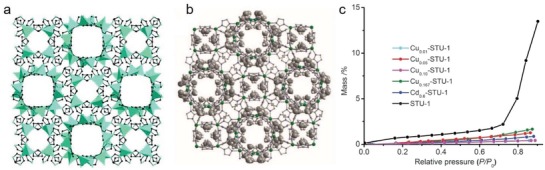
The framework structures of a) STU‐1 and b) STU‐5 and c) water adsorption isotherms of STU‐1 and metal‐ion‐doped STU‐1 samples at 298 K. Adapted with permission.^216^, ^217^, ^218^ Copyright 2011, American Chemical Society for panel (a); and Copyright 2013 and 2016, Royal Society of Chemistry for panels (b) and (c), respectively.

Li and coworkers reported a series of gyroidal MOFs, STU‐1, ‐2, ‐3, and ‐4, formulated as [M(bim)] (M = Zn, Mn, Cu, and Ni, respectively) with the gie topology (**Figure** 25a), which were constructed from the H_2_bim ligand and four types of metal ions, namely, Zn^2+^, Mn^2+^, Cu^2+^, and Ni^2+^, respectively.^216^ Although STU‐1 was found to be stable at temperatures up to 650 °C, it would transform into an unknown phase after being treated with boiling water for 24 h. Later, the same group reported another gyroidal MOF, [Cd(2‐bmim)] (STU‐5), obtained by the in situ generation of the 2‐H_2_bmim ligand from the condensation of 2‐methyl‐1*H*‐imidazole‐4‐carbaldehyde and hydrazine.^217^ STU‐5 contains 1D channels with a pore surface decorated by methyl groups (Figure 25b). The N_2_ adsorption isotherm recorded at 77 K suggested a micropore volume of 0.628 cm^3^ g^−1^ and Langmuir and BET surface areas of 1858 and 1258 m^2^ g^−1^, respectively. Notably, STU‐5 showed not only a high thermal stability but chemical stability. No loss of crystallinity in STU‐5 was observed by PXRD measurements after the sample was treated at 550 °C, boiling water or methanol for 24 h. The authors believed that the introduction of hydrophobic methyl groups on the pore surface resulted in the enhanced water stability of STU‐5 compared to that of STU‐1. Recently, the group reported that the hydrophobicity and water stability of STU‐1 could be improved by doping metal ions (Cu^2+^, Cd^2+^, or Fe^2+^) into its framework.^218^ The metal ions were doped by their introduction to the synthetic reaction mixture of STU‐1, unlike some published works reporting the postsynthetic exchange of metal ions in the frameworks of MOFs.^219^, ^220^ However, doping STU‐1 with Mn^2+^, Co^2+^, and Ni^2+^ by the one‐pot synthesis method was found to be unsuccessful. N_2_ adsorption studies at 77 K revealed that two of the Cu^2+^‐doped STU‐1 samples, Cu_0.05_‐STU‐1 (Zn/Cu: 99/1) and Cu0.10‐STU‐1 (Zn/Cu: 90/10), showed higher porosities than STU‐1, as suggested by their Langmuir (1320, 1407, and 1255 m^2^ g^−1^) and BET surface areas (891, 948, and 775 m^2^ g^−1^). Interestingly, the authors observed that all metal‐ion‐doped STU‐1 samples retained their crystalline structures after being soaked in boiling water for 7 days, as confirmed by both PXRD patterns and gas adsorption measurements. In contrast, STU‐1 transformed to an unknown phase in boiling water after 24 h. Moreover, type VII water adsorption isotherms at 298 K with low uptakes (<17 mg g^−1^ at ≈0.9 *P*/*P*_0_) were observed for the metal‐ion‐doped STU‐1 samples, suggesting their highly hydrophobic internal pore surfaces. In contrast, the water adsorption isotherm of STU‐1 was a type III or incomplete type V isotherm with low uptakes below 0.7 *P*/*P*_0_ and followed by pore filling at higher partial pressures, with an uptake of 136 mg g^−1^ at 0.9 *P*/*P*_0_ (Figure 25c). Indeed, as the authors pointed out, the improvements in the hydrophobicity and water stability of MOFs are normally achieved by incorporating hydrophobic groups onto the pore surface, and such an effect being achieved by doping metal ions was not observed before in MOFs. The mechanism was not fully understood, and it was proposed that the doped metal ions might result in a perturbation of the pore surfaces of MOFs, which hindered the formation of water clusters inside the pores.

Hu et al. reported some N‐coordination‐modified UiO‐66 samples, which were synthesized by introducing dopamine into the synthetic reaction for UiO‐66.^221^ The dopamine‐embedded UiO‐66 samples showed a dark‐brown color rather than colorless (the color of UiO‐66) and their surface charge (ζ potential) in water increased with the amount of dopamine introduced (from 28.4 to 49.5 mV). It was thus proposed that dopamine was introduced into the framework of UiO‐66 by N‐coordination with Zr^4+^ or Zr_6_O_4_(OH)_4_ clusters, and the incorporation of dopamine led to variations in the electron density on UiO‐66. PXRD patterns revealed that the crystal structure of UiO‐66(Zr) remained in the dopamine‐embedded UiO‐66 samples. One of the dopamine‐embedded UiO‐66 samples, M‐UiO‐66(Zr‐N_3.0_), showed a 20% decrease in H_2_O uptake and considerably higher adsorption capacities for harmful volatile organic compounds (VOCs), chlorobenzene and acetaldehyde, than those of UiO‐66, indicating an enhancement in hydrophobicity achieved by introducing dopamine into the structure of UiO‐66; however, the precise structure of the N‐coordination‐modified UiO‐66 was not clear.

According to the reported works, the hydrophobicity of MOFs can be facilely imparted by a postsynthetic treatment or the in situ introduction of certain species into the synthetic systems for the MOFs. Compared to the design and synthesis of new ligands with hydrophobic moieties, the postsynthetic hydrophobization and in situ synthetic hydrophobization methods have advantage in the feasibility and generalizability for the preparation of hydrophobic MOFs, however, the precise structures of the materials prepared by these methods are complicated and the structure‐property relations require further validation.

## 4 Potential Applications

Hydrophobic MOFs possess both porosity and hydrophobicity. This unique attribution renders them highly desired for certain special applications. For example, separation is commonly regarded to be the most important application of MOFs. Hydrophobic MOFs have inherent advantages for some important separation systems in which water is unavoidably involved and brings negative effects to the performance of the adsorbents, such as humid CO_2_ capture separation, alcohol/water separation, and the removal of environmental pollutants from air or water. Hydrophobic MOFs are also a class of special heterogeneous catalysts that show improved catalytic activities for the same reactions and/or selectivities for certain hydrophobic substrates. Due to their low affinity toward water, some highly hydrophobic MOFs essentially show no water uptake, even at saturated water vapor pressures. This attribution can be utilized for storing energy by the forced intrusion of water in their hydrophobic pores at high pressures. The abovementioned applications rely on both the porosity and the internal hydrophobicity of hydrophobic MOFs. On the other hand, many hydrophobic MOFs feature external hydrophobicity. These MOFs are promising coating materials for anticorrosion or self‐cleaning purposes, where the MOFs act more similar to a class of hydrophobic nanomaterials than hydrophobic porous materials. Certainly, there are also some reported studies on hydrophobic MOFs for other applications, such as heat transformation,^142^, ^157^, ^222^, ^223^, ^224^ sensing,^122^, ^225^, ^226^ oil–water separation,^120^, ^127^, ^177^, ^227^, ^228^, ^229^, ^230^, ^231^ enrichment,^232^, ^233^, ^234^, ^235^ and membrane separation,^236^, ^237^ however, those works are not discussed herein for lack of space.

### 4.1 Humid CO_2_ Capture

CO_2_ capture by MOFs has been extensively studied during the last decade.^44^, ^45^ Commonly, different contents of water vapor are present together with CO_2_ in the gases to be separated. For example, a typical untreated flue gas contains 73–77% N_2_, 15–16% CO_2_, 3–4% O_2_, and 5–7% water vapor, as well as other minor gases.^238^ The competitive adsorption of water and its impact to the stability of MOFs have been a major concern for practical applications in CO_2_ capture. Many MOFs have been demonstrated to undergo structural degradation and thus exhibit an impermanent CO_2_ capture performance after being exposed to a humid CO_2_ atmosphere.^175^, ^239^ Hydrophobic MOFs, with an improved water stability and low affinity toward water, are a class of promising adsorbents for CO_2_ capture under humid conditions. Many works on the CO_2_ adsorption of hydrophobic MOFs have been reported.^138^, ^160^, ^196^, ^200^, ^208^, ^212^, ^213^, ^240^, ^241^

**Figure 26:**
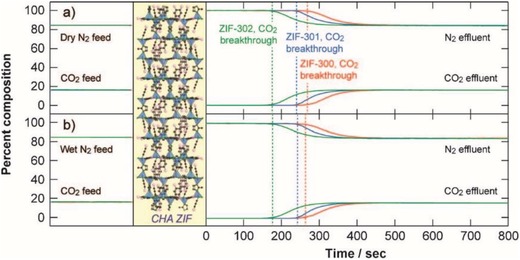
Breakthrough curves for a CO_2_/N_2_ mixture (16:84, v/v) obtained with ZIF‐300 (red), ZIF‐301 (blue), and ZIF‐302 under a) dry and b) wet (80% relative humidity) conditions. The breakthrough time is indicated by the dashed line. Adapted with permission.^138^ Copyright 2014, John Wiley and Sons.

The selective CO_2_ adsorption properties of hydrophobic ZIF‐300, ZIF‐301, and ZIF‐302 were studied by Nguyen et al.^138^ The three MOFs showed very low affinities toward water, as confirmed by their low water uptakes (6, 5.8, and 4.5 mg g^−1^ at *P*/*P*_0_ ≈ 0.8 and 298 K, respectively). CO_2_ and N_2_ adsorption isotherms at 273, 283, and 298 K were recorded for the three MOFs, which indicated CO_2_ uptakes of 40, 40, and 36 cm^3^ cm^−3^ at 298 K and 800 Torr; these values were significantly higher than those for N_2_ adsorption (2.9, 3.8, and 4.0 cm^3^ cm^−3^). The CO_2_/N_2_ adsorption selectivities calculated by the ratios of the initial slopes of adsorption isotherms based on Henry's law were 22, 19, 17 for ZIF‐300, ZIF‐301, and ZIF‐302, respectively. Although their CO_2_ adsorption capacities and CO_2_/N_2_ adsorption selectivities were not high, it was demonstrated that the MOFs showed an uncompromised performance in the selective adsorption of CO_2_ over N_2_ under humid conditions. Dynamic breakthrough experiments of a mixed gas comprising 16% (v/v) CO_2_ and 84% (v/v) N_2_ flowing through fixed beds of the MOF samples were carried out. The breakthrough curves confirmed their CO_2_ capture capabilities, whereby N_2_ passed through the MOFs earlier than CO_2_ (**Figure** 26a). The CO_2_ uptake capacities obtained in the dynamic breakthrough experiments were estimated to be 10.4, 8.0, and 5.5 cm^3^ cm^−3^, which were very dependent on the CO_2_/N_2_ adsorption selectivities calculated using the single‐component gas adsorption isotherms. To test the CO_2_ adsorption performance under humid conditions, the MOF samples were first exposed to a wet N_2_ gas stream (80% relative humidity) until water saturation was detected, and breakthrough experiments were then started by adding dry CO_2_ to the binary gas mixture. Notably, breakthrough curves reproducible with those recorded under dry conditions were observed (Figure 26b), suggesting the negligible effect of water on the CO_2_ capture performance of the hydrophobic MOFs. The authors found that a CO_2_‐saturated ZIF‐300 sample could be regenerated (99.5% of adsorbed CO_2_) under mild conditions, namely, flowing N_2_ at the ambient temperature for 15 min.

**Figure 27:**
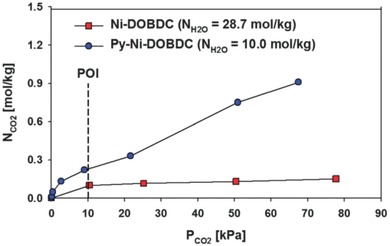
Experimental CO_2_ isotherms at 298 K for Ni‐MOF‐74 and Py‐Ni‐MOF‐74 after preloading H_2_O at ≈45% RH. Adapted with permission.^196^ Copyright 2014, Royal Society of Chemistry.

Bae et al. investigated the CO_2_ adsorption properties for Ni‐MOF‐74 and pyridine‐modified Ni‐MOF‐74 (denoted as Py‐Ni‐MOF‐74), which showed a more hydrophobic pore surface than that of Ni‐MOF‐74.^196^ According to the CO_2_ and H_2_O adsorption isotherms at 298 K, Py‐Ni‐MOF‐74 showed lower CO_2_ and H_2_O uptakes than did Ni‐MOF‐74. At 10 kPa, the CO_2_ uptake of Py‐Ni‐MOF‐74 was less than that of Ni‐MOF‐74 by 40%, but the decrease in H_2_O uptake after the pyridine modification was significantly higher. The effect of water on CO_2_ adsorption was further studied by measuring CO_2_ isotherms after Ni‐MOF‐74 and Py‐Ni‐MOF‐74 were preloaded with H_2_O at 45% relative humidity. Py‐Ni‐MOF‐74 showed higher CO_2_ uptakes than did Ni‐MOF‐74 at all pressures measured. At 10 kPa, the CO_2_ uptake of Py‐Ni‐MOF‐74 was 0.22 mmol g^−1^, over twice that of Ni‐MOF‐74 (0.10 mmol g^−1^) (**Figure** 27). The results clearly showed an improvement in the humid CO_2_ adsorption for the MOF after postsynthetic pyridine modification.

Ding et al. carried out CO_2_ adsorption studies for MOF‐5 and PN@MOF‐5, a hydrophobic derivative of MOF‐5 with polynaphthylene (PN) inside its channels. From the CO_2_ adsorption isotherms recorded at 273 K, PN@MOF‐5 presented a significantly higher uptake (78 cm^3^ g^−1^) than did MOF‐5 (38 cm^3^ g^−1^) at 760 Torr, although the BET surface area of PN@MOF‐5 (1200 m^2^ g^−1^) was considerably lower than that of MOF‐5 (3200 m^2^ g^−1^). While the isosteric heat of CO_2_ adsorption for PN@MOF‐5 decreased from 29 to 24 kJ mol^−1^, clearly higher than those of MOF‐5 (17–19 kJ mol^−1^). The CO_2_ affinity of PN@MOF‐5 being higher than that of MOF‐5 should result from the smaller pore size of the former (6 Å) than that of the latter (12 Å). Moreover, the N_2_ uptake of PN@MOF‐5 (0.8 cm^3^ g^−1^) at 273 K and 800 Torr was substantially lower than that of MOF‐5 (5.2 cm^3^ g^−1^). As a result, the calculated ideal adsorbed solution theory (IAST) CO_2_/N_2_ (14:86, v/v) selectivity at 1 bar and 273 K for PN@MOF‐5 (212) was clearly higher than that of MOF‐5 (9). Dynamic breakthrough experiments of a N_2_/CO_2_ (184:16, v/v) gas mixture for the two MOFs revealed dynamic CO_2_ adsorption capacities of 34 and 23 cm^3^ g^−1^ for PN@MOF‐5 and MOF‐5, respectively. Noteworthily, after the N_2_/CO_2_ gas mixture was humidified (RH = 65%), the dynamic CO_2_ adsorption capacity of PN@MOF‐5 was almost fully retained with respect to that achieved under dry conditions. In contrast, MOF‐5 showed 40% and 70% decreases in dynamic CO_2_ adsorption capacity after the first and second breakthrough tests under the humid condition, respectively. PXRD patterns revealed that MOF‐5 degraded, but PN@MOF‐5 retained its high crystallinity after the breakthrough experiments. It was also demonstrated that the dynamic CO_2_ capacities of MOF‐199 and NH_2_‐UiO‐66 were significantly reduced under similar humid conditions.

### 4.2 Alcohol/Water Separation

Biofuel is an alternative renewable energy source produced from plants through biological processes. The biofuel production processes commonly involve the separation of alcohol and water molecules. Hydrophobic MOFs are a class of promising materials for the selective adsorption of alcohol over water. Both membranes and packed columns of hydrophobic MOFs have been reported for alcohol/water separations.^78^, ^79^, ^176^, ^237^, ^242^, ^243^, ^244^, ^245^, ^246^, ^247^

**Figure 28:**
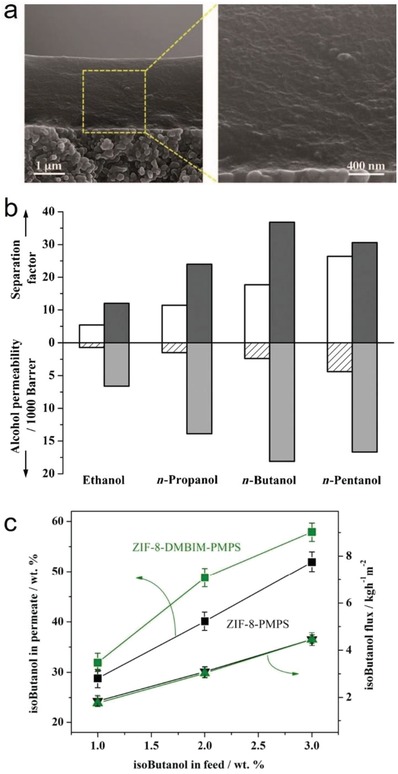
a) SEM images of the cross‐section of a ZIF‐8‐PMPS membrane. b) The separation factor and alcohol permeability of a pure PMPS membrane (open and line filled columns) and a ZIF‐8‐PMPS (gray and light gray columns) membrane for aqueous solutions of C2–C5 alcohols (1.0 wt% alcohols, 80 °C). c) The isobutanol concentrations in permeates (squares) and isobutanol fluxes (triangles) as functions of the feed concentration (upstream side, 80 °C) for the ZIF‐8–PMPS and ZIF‐8–DMBIM–PMPS membranes. Adapted with permission.^176^, ^242^ Copyright 2013, Royal Society of Chemistry for panel (c); and Copyright 2011, John Wiley and Sons for panels (a) and (b).

Liu et al. investigated the performance of a ZIF‐8‐PMPS mixed‐matrix membrane in recovering alcohols from their dilute aqueous solutions.^242^ Vapor adsorption studies showed a type V isobutanol adsorption isotherm for ZIF‐8 at 40 °C with a gate‐opening adsorption jump at 0.5 kPa and an uptake of 360 mg g^−1^ at 3.5 kPa, while no essential adsorption of water occurred below 3.5 kPa (*P*/*P*_0_ ≈ 0.48) due to the highly hydrophobic nature of ZIF‐8. A 2.5 mm‐thick ZIF‐8‐PMPS (weight ratio, W_ZIF‐8_/W_PMPS_ = 0.10:1; PMPS = polymethylphenylsiloxane) mixed‐matrix membrane was fabricated on the inside surface of alumina capillary substrates by the solution‐blending dip‐coating method (**Figure** 28a). Pervaporation experiments were carried out at 80 °C, and showed a high separation factor for isobutanol over water (34.9–40.1) with the ZIF‐8‐PMPS membrane for 1.0–3.0 wt% isobutanol aqueous solutions. In other words, the permeate contains ≈30 wt% isobutanol when a 1.0 wt% isobutanol aqueous solution was applied to the feed side of the ZIF‐8‐PMPS membrane. The isobutanol permeance of the ZIF‐8‐PMPS membrane was high up to 6000–7000 GPU (1 GPU = 1 × 10^−6^ cm^3^ (STP) cm^−2^ s^−1^ cmHg^−1^ or 3.35 × 10^−10^ mol m^−2^ s^−1^ Pa^−1^). The pervaporation performances of the ZIF‐8‐PMPS membrane in recovering ethanol, *n*‐propanol, *n*‐butanol, and *n*‐pentanol from water were also evaluated (Figure 28b). The alcohol/water selectivities and permeabilities of alcohols were all higher than those for a pure PMPS membrane. The same group later prepared a more hydrophobic and water‐stable MOF, ZIF‐8–DMBIM, by the external surface postmodification of ZIF‐8 with the ligand DMBIM^−^.^176^ The performance of ZIF‐8–DMBIM in the recovery of isobutanol from aqueous solutions was also evaluated. The isobutanol sorption isotherms of ZIF‐8–DMBIM at 40 °C showed a slightly lower uptake at 3.5 kPa than did ZIF‐8, but the gate‐opening pressure for the isobutanol sorption of ZIF‐8–DMBIM was evidently decreased compared to that of ZIF‐8. This observation was attributed to an improvement in the isobutanol transport diffusivity of ZIF‐8–DMBIM. After being treated by a 3.0 wt% isobutanol aqueous solution at 80 °C for 24 h, ZIF‐8–DMBIM retained its isobutanol uptakes after 5 cycles, but the isobutanol uptake of ZIF‐8 reduced to almost 0 at the third cycle. Similarly, a ZIF‐8–DMBIM–PMPS membrane was prepared, and pervaporation experiments for the separation of isobutanol and water were carried out with the membrane. The permeate contains ≈58 wt% isobutanol when a 2.0 wt% isobutanol aqueous solution was applied to the feed side of the ZIF‐8–DMBIM–PMPS membrane. The concentration was 1.4 times higher than that obtained by vapor–liquid equilibrium (VLE) evaporation. Higher separation selectivities were observed for the ZIF‐8–DMBIM–PMPS membrane than were observed for the ZIF‐8–PMPS membrane, and the fluxes were not compromised (Figure 28c). The researchers believed that the improved selectivity resulted from the increased hydrophobicity and stability of ZIF‐8–DMBIM with respect to those of ZIF‐8.

Zhang et al. recorded the adsorption isotherms of water, methanol, ethanol, 1‐propanol, 2‐propanol, and 1‐butanol for three hydrophobic MOFs, ZIF‐8, ZIF‐71, and ZIF‐90.^79^ Characteristic S‐shaped type V isotherms at 308 K were observed for methanol, ethanol, and 1‐propanol adsorption in ZIF‐8, all the alcohol adsorptions in ZIF‐71, and the methanol adsorption in ZIF‐90. From the adsorption isotherms, the affinities between the adsorbate–adsorbent pairs can be compared. The higher the critical pressure (above which pore‐filling occurs) is, the stronger the adsorbate–adsorbent interaction. Water adsorption isotherms were also recorded at 308 K for the three MOFs, showing type III isotherms for ZIF‐8 and ZIF‐71 and a type V isotherm for ZIF‐90. It was suggested that ZIF‐71 was most hydrophobic among the three MOFs, and ZIF‐90 was less hydrophobic than ZIF‐8 and ZIF‐71. The IAST adsorption selectivities of binary alcohol–water vapor mixtures were calculated for the three MOFs. The results revealed that ZIF‐8 and ZIF‐71 exhibit promising separation performances for 1‐butanol–water, 1‐propanol–water, and 2‐propanol–water pairs. The highest IAST adsorption selectivity was obtained for the 1‐butanol–water system in ZIF‐71. For a 0.25 mol% 1‐butanol (and 99.75 mol% water) feed, the IAST adsorption selectivity of ZIF‐71 for vapor‐phase 1‐butanol–water was estimated to be up to 290. However, the calculated IAST adsorption selectivities for the methanol–water system were low for both ZIF‐8 and ZIF‐71.

**Figure 29:**
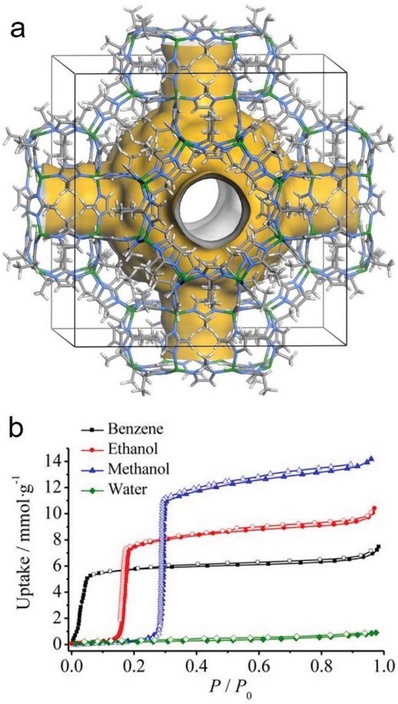
Framework structure of a) MAF‐6 and b) its benzene, ethanol, methanol, and water adsorption isotherms measured at 298 K. Adapted with permission.^78^ Copyright 2015, American Chemical Society.

He et al. investigated the water, methanol, ethanol, and benzene sorption properties at room temperature for a highly porous and highly hydrophobic MOF with the RHO zeolitic topology, MAF‐6 (**Figure** 29a).^78^ A type III water adsorption isotherm was observed for MAF‐6, which showed very low uptakes, even at *P*/*P*_0_ = 0.97 (0.90 mmol g^−1^ or 16.2 mg g^−1^) (Figure 29b). Type V (S‐shaped) isotherms with saturation uptakes of 9.15 and 13.27 mmol g^−1^ were observed for methanol and ethanol, respectively. The benzene adsorption isotherm of MAF‐6 showed a type IV characteristic, providing a saturation uptake of 6.36 mmol g^−1^. The critical pressures (above which pore‐filling occurs) for benzene, ethanol, and methanol adsorption were 0.02, 0.14, and 0.26 *P*/*P*_0_, respectively (Figure 29b). The difference between the critical pressures for ethanol and methanol adsorption in MAF‐6 (0.26−0.14 = 0.12) is higher than that of the other two representative MOFs, MAF‐4/ZIF‐8 (0.10−0.05 = 0.05), and ZIF‐71 (0.20−0.12 = 0.08).^79^ The adsorption results suggested the application potential of MAF‐6 in alcohol/water and even methanol−ethanol separations.

### 4.3 The Removal of Environmental Pollutants from Air or Water

The removal of pollutants in air or water by porous adsorbents has received tremendous attention for several decades.^46^, ^47^, ^248^ As water is ubiquitous, the desired adsorbents need to be capable of capturing environmental pollutants effectively, even when they are exposed to high humidity or immersed in liquid water. Hydrophobic MOFs are promising materials in this regard.

In 2008, Britt et al. investigated the performance of six MOFs, namely, MOF‐5, IRMOF‐3, Zn‐MOF‐74, MOF‐177, MOF‐199 (HKUST‐1), and IRMOF‐62, in the capture of eight harmful gases: sulfur dioxide, ammonia, chlorine, tetrahydrothiophene, benzene, dichloromethane, ethylene oxide, and carbon monoxide by breakthrough experiments.^249^ It was found that the two MOFs with open metal sites, Zn‐MOF‐74 and MOF‐199 (HKUST‐1), and the MOF with the amino functionality were effective in the capture of some of these harmful gases including ammonia and ethylene oxide; however, carbon monoxide could not be effectively captured by all the MOFs tested. Especially, MOF‐199 (HKUST‐1) showed an improved performance over that of BPL carbon in the capture of all the gases except chlorine. However, all the results were obtained from breakthrough experiments under dry conditions, and the performance of these MOFs in the capture of humid harmful gases was not tested.

Navarro and co‐workers reported the performance of a MOF‐5‐type hydrophobic MOF, Zn‐dmpc, in the capture of the harmful VOCs DIFP and DES, which are model compounds for the chemical warfare agents sarin nerve gas and mustard vesicant gas, respectively.^155^ The authors found that Zn‐dmpc was capable of capturing the harmful VOCs, even under humid conditions, and its performance was comparable to that of Carboxen (an activated carbon material). In addition, Zn‐dmpc outperformed HKUST‐1 because the open metal sites in HKUST‐1 were ineffective for the capture of VOCs under humid conditions. The same group later reported a series of MOFs with the 12‐connected fcu nets, [Ni_8_(L)_6_] ([Ni_8_(L_3_)_6_], [Ni_8_(L_4_)_6_], [Ni_8_(L_5_)_6_], [Ni_8_(L_5_‐CH_3_)_6_], and [Ni_8_(L_5_‐CF_3_)_6_], which showed high porosities (BET surface areas: 205–2215 m^2^ g^−1^), high stabilities in water and basic aqueous solutions, and tunable hydrophobicities.^140^ The MOFs also showed great potential in the capture of DES, a model compound for the chemical warfare agent mustard gas, from dry and humid Ar/N_2_ streams.

**Figure 30:**
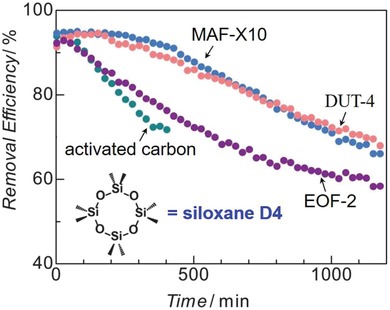
Breakthrough curves for siloxane D4 with MAF‐X10, DUT‐4, EOF‐2, and activated carbon (green) at room temperature and 50% relative humidity. Adapted with permission.^252^ Copyright 2013, Royal Society of Chemistry.

Mito‐oka et al. reported a study of two hydrophobic MOFs, MAF‐X10^157^, ^158^ and DUT‐4 (formulated as [Al(OH)(2,6‐ndc)]),^250^ a porous organic framework poly(4,4′‐biphenylene)silane, EOF‐2,^251^ and a conventional activated carbon for the selective adsorption of siloxane D4, which is one of the major impurities in biogas.^252^ The breakthrough experiments of siloxane D4 (5 ppm in air) under humid conditions (50% relative humidity) were carried out to evaluate the performances of the adsorbents. MAF‐X10 and DUT‐4 showed strong adsorption selectivities for ppm levels of siloxane D4 at high humidity, as indicated by the retention of high removal efficiencies (**Figure** 30). However, the removal efficiencies of EOF‐2 and activated carbon decayed more rapidly, and this decay was attributed to their weaker interaction with siloxane D4 and/or the competitive adsorption of water.

**Figure 31:**
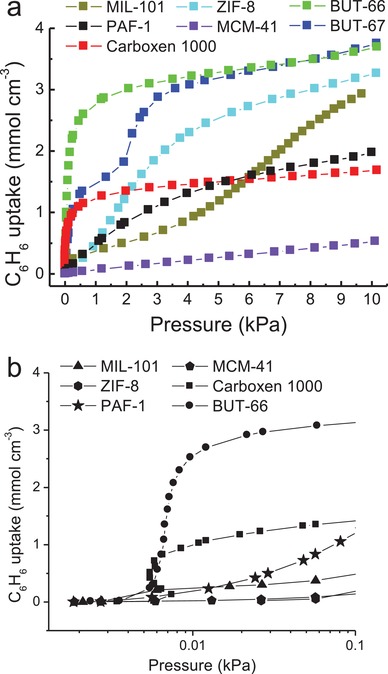
Benzene vapor adsorption isotherms recorded at 80 °C for Carboxen 1000, MIL‐101(Cr), ZIF‐8, MCM‐41, PAF‐1, BUT‐66, and BUT‐67 for the a) entire pressure range and b) the low‐pressure range. Adapted with permission.^88^ Copyright 2018, Elsevier.

Xie et al. recently evaluated the performance of 7 hydrophobic adsorbents (4 MOFs, MIL‐101(Cr), ZIF‐8, BUT‐66, and BUT‐67; a mesoporous silica, MCM‐41; a microporous organic polymer, PAF‐1; and a commercial carbon molecular sieve, Carboxen 1000) in the capture of trace benzene (10 ppm) from air.^88^ From benzene vapor adsorption studies, PAF‐1, MIL‐101(Cr), ZIF‐8, and MCM‐41 revealed high benzene uptakes at high pressures, but considerably lower uptakes in the low‐pressure range. In contrast, the benzene adsorption isotherms of Carboxen 1000, BUT‐66 and BUT‐67 resembled a typical type I isotherm, indicative of strong adsorbate–adsorbent interactions (**Figure** 31a). Especially, BUT‐66 showed a benzene uptake up to 1.75 mmol cm^−3^ at low pressure (0.12 kPa) and high temperature (80 °C), which is the highest among those of all the tested adsorbents (Figure 31b). Type I adsorption isotherms for toluene, ethylbenzene, *o*‐xylene, *m*‐xylene, and *p*‐xylene were also observed with BUT‐66 at 80 °C, showing high uptakes at low pressures. The results suggested the great potential of BUT‐66 for capturing those harmful aromatic VOCs in air, regardless of their concentration. The ability of BUT‐66 to capture low‐concentration benzene in ambient air was confirmed by gas breakthrough experiments of a gas mixture (10 ppm benzene in air) flowing through a packed sample of BUT‐66. The breakthrough curves suggested a benzene capture capacity of ≈0.27 mmol g^−1^ in the dynamic adsorption process at room temperature, and the capture capacity was not significantly reduced under humid conditions. The high performance of BUT‐66 in the trace benzene capture was attributed to a moderate hydrophobicity and the local flexibility of its framework, according to the single‐crystal structures of guest‐loaded phases.

In addition to the pollutants in air, hydrophobic MOFs have also been applied to remove pollutants in water. Jhung et al. early investigated the performance of MIL‐101(Cr) in the removal of benzene from water.^253^ The sorption isotherms of benzene were recorded for MIL‐101(Cr) and active carbon with aqueous solutions containing 1000 ppm benzene at 25 °C. A high adsorption capacity and fast adsorption kinetics were observed for MIL‐101(Cr) compared with those of active carbon, which was attributed to the effect of the large pores in MIL‐101(Cr). Wang et al. applied a bipyrazole ligand‐based hydrophobic MOF, Cu‐tebpz, to extract trace C6–C8 aromatic hydrocarbons from water.^141^ The adsorption saturation of benzene was achieved within 15 min, and the concentration of benzene was ultimately 0.0056 µL mL^−1^ (2.2% remaining), after Cu‐tebpz was placed in a 0.25 µL mL^−1^ aqueous solution. The performance was stable for five cycles of benzene extraction while the MOF was regenerated by heating under vacuum. Moreover, the researchers found that trace xylene (o/m/p = 1:1:1, v/v/v, ≈0.08 µL mL^−1^) in water could be completely removed by Cu‐tebpz (15 mg) after 10 min.

Chun et al. studied the adsorption of toluene in water by UiO‐66‐NH_2_ and 4 hybrid microporous materials, MOF@MON‐1, MOF@MON‐2, MOF@MON‐3, and MOF@MON‐4, which were obtained by coating the external surface of UiO‐66‐NH_2_ with microporous organic networks (MONs).^207^ It was found that MOF@MONs provided toluene adsorption capacities that were considerably higher than that of UiO‐66‐NH_2_, although UiO‐66‐NH_2_ showed higher adsorption capacities for both pure water and pure toluene than did MOF@MON‐1. The results indicated the presence of an adsorption competition between water and toluene in the adsorbents. Many studies have also shown the great potential of hydrophobic MOFs or their derived materials for bulk oil/water separation^127^, ^229^ and bulk oil adsorption from water for the cleanup of oil spills.^120^, ^177^, ^227^, ^228^, ^230^, ^231^, ^254^ The removal of other types of harmful or useful gases or organic substances, such as ethylene,^232^ acetic acid,^255^ thiophene,^180^ peptides,^233^ aromatic amino acids,^234^ and pharmaceuticals,^122^, ^235^, ^256^, ^257^ from air or water by hydrophobic MOFs and their derived materials has also been documented.

### 4.4 Catalysis

**Figure 32:**
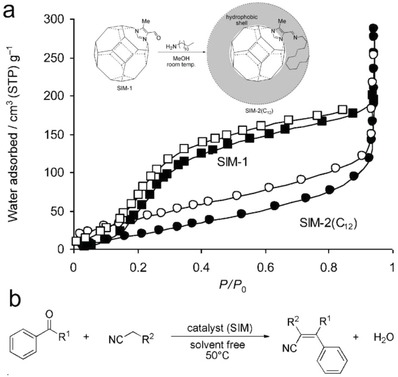
a) Water adsorption isotherms of SIM‐1 and SIM‐2(C12) at 303 K. b) The Knoevenagel condensation reaction catalyzed by SIM‐2(C12). Adapted with permission.^260^ Copyright 2011, John Wiley and Sons.

Among numerous reported studies on MOFs as catalysts, some works have demonstrated the high catalytic performance of hydrophobic MOFs.^258^, ^259^ In particular, due to their hydrophobic nature, some hydrophobic MOFs showed a selective catalytic activity for certain specific substrates. Aguado et al. reported a zinc carboxylimidazolate, denoted as SIM‐1 and formulated as [Zn(2‐mimc)_2_], which is isostructural to ZIF‐8 with the SOD zeolitic topology.^260^, ^261^, ^262^ SIM‐1 was obtained by the solvothermal reaction of zinc salt and the ligand 2‐Hmimc in DMF. Due to the presence of free aldehyde groups on the pore surface, SIM‐1 could be postmodified by the reaction of aldehyde groups and dodecylamine, with long alkyl C_12_ chains, resulting in the imino‐functionalized SIM‐2(C_12_). The ^1^H NMR analysis results indicated that approximately one‐fifth of the ligands in SIM‐2(C_12_) were grafted with the C_12_ chains. SIM‐2(C_12_) showed a good hydrophobicity compared to that of SIM‐1, as indicated by their water adsorption isotherms and water contact angles (**Figure** 32a). The water adsorption isotherms at 303 K showed that SIM‐2(C_12_) had lower uptakes for the entire pressure range (pore filling occurred at a high pressure of ≈0.9 *P*/*P*_0_) and a larger water contact angle (155° vs 85°) than that of SIM‐1. The catalytic activities of ZIF‐8, SIM‐1, SIM‐2(C_12_), and a reference molecular catalyst (dodecylamine) were tested in the Knoevenagel reaction (Figure 32b). The results of the catalytic reactions suggested that SIM‐2(C_12_) was the most active catalyst. The authors speculated that the active centers were on the external crystal surfaces of SIM‐2(C12), and the tenfold increase in catalytic activity of SIM‐2(C_12_) over that of SIM‐1 resulted from the presence of a hydrophobic environment surrounding the catalytic sites, as found in enzymes.

**Figure 33:**
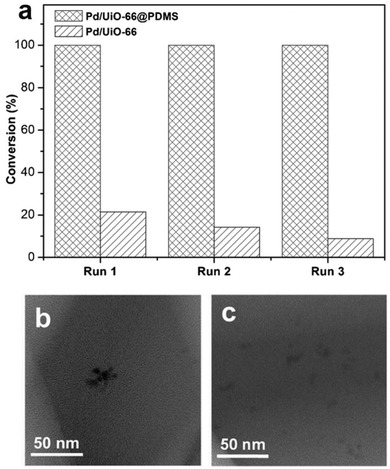
a) Catalytic conversions of 3 repeated runs of the styrene hydrogenation reaction with Pd/UiO‐66 and Pd/UiO‐66@PDMS as the catalyst. TEM images for b) Pd/UiO‐66 and c) Pd/UiO‐66@PDMS after recycling experiments. Adapted with permission.^263^ Copyright 2016, John Wiley and Sons.

Jiang and co‐workers reported a PDMS‐coated MOF with immobilized Pd nanoparticles, Pd/UiO‐66@PDMS, obtained by a chemical vapor deposition (CVD) approach whereby a sample of Pd/UiO‐66 nanocomposite (0.71 wt% Pd loading) together with a PDMS stamp were heated in a sealed glass container at 200 °C.^263^ The N_2_ adsorption isotherms at 77 K of Pd/UiO‐66 and Pd/UiO‐66@PDMS were similar in profile, and Pd/UiO‐66@PDMS showed a slightly lower BET surface area than did Pd/UiO‐66, suggesting that the PDMS layer on the surface of Pd/UiO‐66 was thin and permeable. After applying the PDMS coating, the surface of Pd/UiO‐66 turned from hydrophilic to hydrophobic, as indicated by their water contact angles (from 25° to 140°). The surface hydrophobicity of Pd/UiO‐66@PDMS was further supported by the fact that it could be gradually transferred from the aqueous phase to the organic phase in a water–ethyl acetate biphasic mixture. Compared with Pd/UiO‐66, the authors found that Pd/UiO‐66@PDMS exhibited a significantly improved catalytic efficiency for styrene hydrogenation reaction. The complete hydrogenation of styrene catalyzed by Pd/UiO‐66 took 255 min, but only 65 min was needed when Pd/UiO‐66@PDMS was used as the catalyst. The authors also pointed out that too thick of a PDMS coating decreased the catalytic efficiency of Pd/UiO‐66@PDMS. In addition, the catalytic activity of Pd/UiO‐66 gradually decreased after 3 repeated runs of the styrene hydrogenation reaction (**Figure** 33a), which was attributed to the gradual aggregation of Pd nanoparticles inside Pd/UiO‐66 during the catalytic processes (Figure 33b). In contrast, the catalytic activity of Pd/UiO‐66@PDMS was stable during repeated runs of the styrene hydrogenation reaction (Figure 33a). It was believed that the Pd nanoparticles in Pd/UiO‐66@PDMS were stabilized by the PDMS coating layer, as indicated by the retention of the Pd nanoparticle size, as well as the crystallinity and porosity of Pd/UiO‐66@PDMS, after catalysis (Figure 33c). The PDMS coating approach was also applied to Pd/C and Pd/SiO_2_ catalysts. The PDMS‐coated Pd/C and Pd/SiO_2_ also showed improved catalytic efficiencies and recyclability. Noteworthily, the selective conversion of hydrophobic reactants over hydrophilic ones to their respective products was observed for the PDMS‐coated catalysts. The complete hydrogenation of hydrophobic nitrobenzene was achieved in 60 min, but the hydrogenation of hydrophilic 4‐nitrophenol did not proceed when Pd/UiO‐66@PDMS was used as the catalyst. In contrast, Pd/UiO‐66 did not show such a selectivity. Both nitrobenzene and 4‐nitrophenol were reduced by Pd/UiO‐66, and the hydrogenation reaction was substantially faster for 4‐nitrophenol than for nitrobenzene.

**Figure 34:**
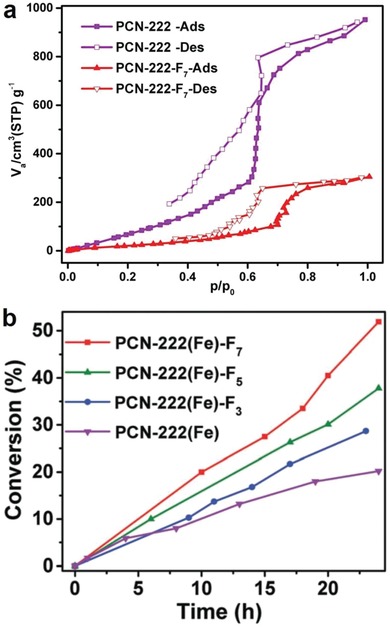
a) Water adsorption isotherms for PCN‐222(Fe) and PCN‐222(Fe)‐F_7_ at 298 K. b) The time‐dependent conversions for the cyclohexane oxidation reactions catalyzed by PCN‐222(Fe), PCN‐222(Fe)‐F_3_, PCN‐222(Fe)‐F_5_, and PCN‐222(Fe)‐F_7_. Adapted with permission.^264^ Copyright 2017, Royal Society of Chemistry.

The same group later reported that the postsynthetic hydrophobization of a porphyrinic Zr(IV)‐based MOF, PCN‐222(Fe) (formulated as [Zr_6_(µ_3_‐O)_8_(µ_3_‐OH)_4_(COO)_8_(OH)_4_(H_2_O)_8_(tcpp)_2_]), with perfluoroalkyl acids resulted in its enhanced catalytic activity and selectivity for the oxidation of cyclohexane to cyclohexanone and cyclohexanol (known as KA oil).^264^ PCN‐222(Fe)^265^ (also known as MOF‐545^266^ or MMPF‐6^267^) is an 8‐connected framework with the csq topology that is built from Zr_6_(µ_3_‐O)_8_(µ_3_‐OH)_4_(COO)_8_(OH)_4_(H_2_O)_8_ clusters and the tetratopic porphyrinic ligand TCPP^4−^; the MOF contains small triangular (8 Å) and large hexagonal (36 Å) 1D channels.^268^ PCN‐222(Fe) was hydrophobized by replacing terminal OH^−^ groups on its Zr_6_(µ_3_‐O)_8_(µ_3_‐OH)_4_(COO)_8_(OH)_4_(H_2_O)_8_ clusters with perfluorocarboxylate, trifluoroacetate, pentafluoropropionate or heptafluorobutyrate groups, giving PCN‐222(Fe)‐F_3_, PCN‐222(Fe)‐F_5_, and PCN‐222(Fe)‐F_7_, respectively. PCN‐222(Fe)‐F_3_, PCN‐222(Fe)‐F_5_, and PCN‐222(Fe)‐F_7_ showed lower surface areas than did the pristine PCN‐222(Fe), but their water contact angles were 51°, 110°, and 137°, respectively, significantly higher than that of PCN‐222(Fe) (≈10°). The hydrophobicity of PCN‐222(Fe)‐F*_n_* was further supported by the results from water adsorption (**Figure** 34a), dispersion of the MOFs in a water–cyclohexane biphasic mixture, and liquid cyclohexane adsorption studies. The oxidation reactions of cyclohexane were performed with the MOFs as catalysts and *tert*‐butyl hydroperoxide as an oxidant. PCN‐222(Fe)‐F*_n_* all showed improved catalytic activities (conversion: 45–50%) over that of PCN‐222(Fe) (conversion: 21%), especially PCN‐222(Fe)‐F_7_, as the one with the longest perfluoro chain (Figure 34b). In contrast, PCN‐222, without Fe(III) ions located in its porphyrin centers, and the homogeneous iron porphyrin were found to be nearly inactive toward the reaction. The authors proposed that a driving force to enrich the hydrophobic reaction substrates was induced by the hydrophobic inner pore surface, which promoted the catalytic reactions.

**Figure 35:**
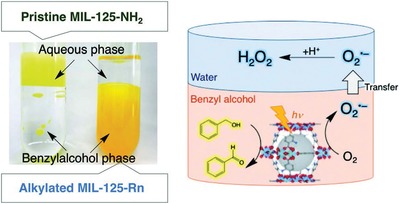
Digital photograph of the hydrophilic pristine MIL‐125‐NH_2_ in the aqueous phase of a benzylalcohol/water two‐phase system and of the hydrophobized MIL‐125‐NH_2_ in the organic phase of the two‐phase system (left); a schematic representation of the photocatalytic H_2_O_2_ production reaction with a hydrophobic MOF catalyst carried out in the benzylalcohol/water two‐phase system. Adapted with permission.^270^ Copyright 2019, John Wiley and Sons.

Zu et al. reported the preparation of hydrophobic HKUST‐1/graphite oxide composites and applied the composites toward the catalytic production of 2‐methoxy‐2‐phenylethanol from the ring opening of styrene oxide.^215^ It was found that HKUST‐1/graphite oxide composites showed improvements in both the hydrothermal stability and catalytic activity compared to those of HKUST‐1. The conversion of styrene oxide to 2‐methoxy‐2‐phenylethanol was 74.1% after the reaction proceeded with HKUST‐1/graphite oxide as the catalyst for the 20 min; in contrast, the conversion was only 10.7% for HKUST‐1 as the catalyst under the same reaction conditions. Abedi et al. reported 4 isostructural MOFs, TMU‐6(L1), TMU‐21(L2), TMU‐6(RL1), and TMU‐21(RL2), with slight differences in structure and internal hydrophobicity.^269^ The MOFs were used as catalysts for the aldol condensation reactions of malononitrile with ketone‐functionalized carbonyl substrates. The researchers found that the slight difference in internal hydrophobicities of the MOFs resulted in their preference for specific substrates in the aldol condensation reaction. Ying et al. reported the core‐shell‐structured MIL‐101(Cr)@mSiO_2_ obtained by coating MIL‐101(Cr) with a thin layer (≈30 nm) of hydrophobic mesoporous silica (mSiO_2_).^204^ Both MIL‐101(Cr) and MIL‐101(Cr)@mSiO_2_ were used as catalysts for two reactions: the oxidation of 1‐dodecene in acetonitrile (reaction I) and the dehydration of glucose in water (reaction II). It was observed that MIL‐101(Cr)@mSiO_2_ showed a significantly improved catalytic activity for reaction I, where a hydrophobic substrate and solvent were involved, and a lower catalytic activity for reaction II, where a hydrophilic substrate and solvent were involved. Logan et al. reported the *N*‐alkyl functionalization of a photoredox‐active MOF, [Ti_8_O_8_(OH)_4_(1,4‐bdc‐NH_2_)_6_] (MIL‐125‐NH_2_), leading to a series of *N*‐alkyl analogs, MIL‐125‐NHR, where R represents methyl ethyl, isopropyl, *n*‐butyl, cyclopentyl, cyclohexyl, and *n*‐heptyl groups.^166^, ^167^ Due to the introduction of hydrophobicity on pore surface and the inductive effects of the *N*‐alkyl groups, MIL‐125‐NHR showed improved stabilities and increased reaction rates and quantum yields in the photocatalytic reduction of carbon dioxide under blue light. Yamashita and coworkers recently reported that a photoactive MOF, MIL‐125‐NH_2_, could be hydrophobized by the alkylation of its linkers with different acid anhydrides^270^ or by the modification of its outermost surface with octadecylphosphonic acid (OPA)^271^ and that these hydrophobic phases exhibited high photocatalytic activities in the production of H_2_O_2_, which was carried out in a two‐phase system (benzylalcohol/water). Thanks to their hydrophobicity, the MOF catalysts stayed in the organic phase (**Figure** 35), and the highly acidic aqueous phase could be employed to facilitate H_2_O_2_ production. Moreover, during the reaction, the reaction products, H_2_O_2_ and benzaldehyde, were spontaneously separated and dissolved in the aqueous phase and organic phase, respectively.

### 4.5 Energy Storage

Hydrophobic microporous materials have been regarded as a class of promising energy storage materials, which store energy by the forced intrusion of water in their hydrophobic pores at high pressures.^272^ In such a way, molecular springs, bumpers and shock‐absorbers may be designed by the hydrophobic adsorbents to accumulate, restore and dissipate mechanical energy. Research studies on hydrophobic zeolites for this application began to emerge in 2001.^273^, ^274^, ^275^, ^276^, ^277^, ^278^, ^279^ Recently, two representative hydrophobic MOFs, ZIF‐8 and ZIF‐71, have also been evaluated for their potential use in energy storage.

**Figure 36:**
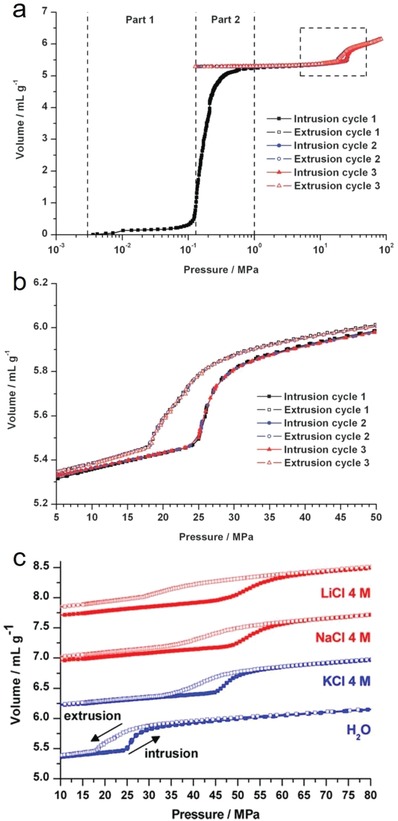
Water intrusion–extrusion diagrams for a) the ZIF‐8–water system with a logarithmic scale for pressure values and b) the enlargement of the pressure range from 5 to 50 MPa. c) Intrusion–extrusion diagrams for 4 m LiCl, NaCl, KCl aqueous solutions in ZIF‐8 and compared with that for pure water. Adapted with permission.^280^, ^283^ Copyright 2013, Royal Society of Chemistry for panels (a) and (b); and Copyright 2014, American Chemical Society for panel (c).

Ortiz et al. first reported high‐pressure water intrusion–extrusion experiments for ZIF‐8.^280^ Three steps of water intrusion were observed in the water intrusion–extrusion diagram of ZIF‐8 (**Figure** 36a,b). The authors proposed that the first step occurred from 0.003 to 0.13 MPa, the second step occurred from 0.13 to 1 MPa, and the third step started at 27 MPa, which resulted from the compressibility of the ZIF‐8 particles, water intrusion in interparticle voids, and water intrusion in the pores of ZIF‐8, respectively. Approximately 0.5 cm^3^ g^−1^ water was intruded into the pores of ZIF‐8, slightly lower than the pore volume of ZIF‐8 calculated from the N_2_ adsorption isotherms (0.59–0.63 cm^3^ g^−1^). The difference was explained by the fact that bulk water density at high pressures is lower than 1 g mL^−1^. The gradual increase in water intrusion after the complete pore filling of ZIF‐8 from 40 to 80 MPa was believed to result from a weak compression of the ZIF‐8–water system. The water intrusion–extrusion process was reversible, and a weak hysteresis was observed. Three repeated cycles of water intrusion–extrusion measurements revealed that the water intrusion–extrusion process was reproducible. PXRD patterns, SEM images and N_2_ adsorption isotherms, before and after the water intrusion–extrusion experiments, confirmed that ZIF‐8 remained stable after the treatments of 80 MPa force. According to the results, the authors concluded that ZIF‐8 could serve as a shock‐absorber and was capable of storing 13.3 J g^−1^ energy, which corresponded to the work (W) made by a mechanical displacement force and can be calculated by Equation (5), where *P* is the pressure and *V* is the intruded volume. The performance of ZIF‐8 was close to that of some other zeolites,^281^, ^282^ but the water intrusion started at a significantly lower pressure for ZIF‐8 (27 MPa) than that for those zeolites (130–190 MPa), potentiating new energy storage applications. The authors later investigated the high‐pressure intrusion−extrusion of aqueous KCl, LiCl, and NaCl solutions with ZIF‐8.^283^ It was found that the intrusion of the aqueous electrolyte solutions into the pores of ZIF‐8 occurred at higher pressures (29–52 MPa) than that for pure water intrusion (27 MPa) (Figure 36c), and the stored energy for a ZIF‐8–NaCl solution (4 m) system was increased to 26.0 J g^−1^

(5) $$\text{W}=\int_{0}^{V}-P\text{d}V$$

**Figure 37:**
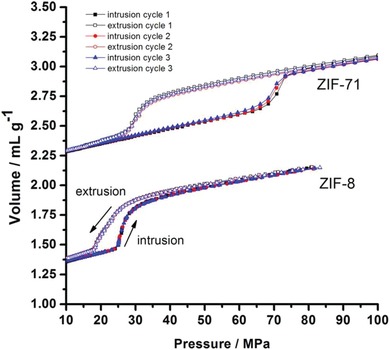
Water intrusion–extrusion diagrams for the ZIF‐71−water and ZIF‐8–water systems. Adapted with permission.^284^ Copyright 2014, American Chemical Society.

Ortiz et al. also carried out water intrusion−extrusion experiments for another hydrophobic MOF, ZIF‐71.^284^ It was observed that water intrusion started at ≈71 MPa, and 0.36 cm^3^ g^−1^ water was intruded into the pores of ZIF‐71, slightly lower than the pore volume of ZIF‐71 calculated from the N_2_ adsorption isotherm (0.39 cm^3^ g^−1^). Repeated water intrusion−extrusion experiments revealed fully reversible water intrusion−extrusion processes and a prominent hysteresis whereby the extrusion of water started at 30 MPa. It was suggested that the ZIF‐71−water system could serve as a shock‐absorber with an energy storage capacity of 26.0 J g^−1^, which was approximately twice of that achieved for the ZIF‐8−water system (**Figure** 37). The intrusion−extrusion experiments for a ZIF‐71−KCl aqueous solution systems were also investigated. The intrusion pressure increased to 74 and 96 MPa for 1 m and 4 M KCl aqueous solutions, respectively. However, the crystalline structure of ZIF‐71 collapsed after the intrusion−extrusion experiment with the 4 m KCl aqueous solution.

### 4.6 Anticorrosion

**Figure 38:**
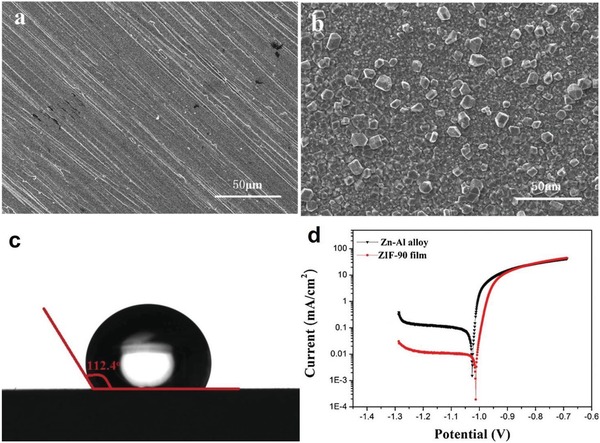
SEM images of the a) bare Zn–Al sheet and b) ZIF‐90 film on the Zn–Al alloy. c) Water contact angle of the ZIF‐90‐coated Zn–Al alloy. d) Potentiodynamic polarization curves of the bare Zn–Al alloy sheet and ZIF‐90‐coated Zn–Al alloy in 3.5% NaCl solutions after a 30 min immersion. Adapted with permission.^286^ Copyright 2018, Elsevier.

Although less attention has been focused on this topic, hydrophobic MOFs have showed potential in the anticorrosion of metals when they are grown on metallic surfaces.^285^ Zhang et al. reported the coating of a Zn–Al alloy with a layer of ZIF‐90 crystals, which improved the corrosion resistance of the Zn–Al alloy in a 3.5 wt% NaCl aqueous solution.^286^ The ZIF‐90 film was prepared by placing a Zn–Al sheet in the solvothermal reaction solution for ZIF‐90. After the reaction, a yellowish layer was formed and was confirmed to be ZIF‐90 by PXRD patterns. SEM images showed a continuous and uniform ZIF‐90 layer consisting of well‐intergrown polyhedron crystals completely coated on the surface of the Zn–Al sheet (**Figure** 38a,b). The adhesion between the ZIF‐90 film and the Zn–Al alloy substrate was tested because it is an important factor determining the durability of a thin film. The ZIF‐90‐coated Zn–Al alloy was subject to a cross‐cut treatment with a BYK5123 cross‐cut tester. A microscope photograph of the cross‐cut ZIF‐90 layer showed that the cuts were completely smooth and none of the squares of the lattice between the cuts were detached, suggesting a level 0 adhesion for the film (according to ISO 2409:2013). It was found that the water contact angles of the Zn–Al alloy and the ZIF‐90‐coated Zn–Al alloy were 71° and 112.4°, respectively, which indicated an enhancement in the hydrophobicity of the Zn–Al alloy surface with the ZIF‐90 coating (Figure 38c). The authors proposed that the large water contact angle of the ZIF‐90‐coated Zn–Al alloy partly resulted from the crystal structure of ZIF‐90 and partly resulted from the surface morphology of the ZIF‐90 film, whereby a large volume of air could be trapped between the micrometer peaks and valleys of the intergrown ZIF‐90 crystals. The corrosion resistance of the ZIF‐90‐coated Zn–Al alloy was tested by potentiodynamic polarization, which showed an *I*_corr_ of 4.266 × 10^−6^ A cm^−2^; this value was two orders of magnitude lower than that of the bare Zn–Al (1.051 × 10^−4^ A cm^−2^) (Figure 38d). The results suggested that the corrosion resistance of the Zn–Al alloy in a 3.5 wt% NaCl aqueous solution was greatly enhanced after the ZIF‐90 coating and was a result of the nonwetting surface induced by the ZIF‐90 coating.

Zhang et al. recently reported the significantly improved anticorrosion performance of a ZIF‐8‐coated Al substrate compared to that of the bare Al substrate.^287^ The ZIF‐8 layer was prepared by the solvothermal reaction of a ZnAl‐CO_3_ layered double hydroxide (LDH) precursor buffer layer on the Al substrate and the ligand 2‐Hmim. Before growing the ZIF‐8 layer, a ZnAl‐CO_3_ LDH buffer layer was prepared by introducing an Al plate to the hydrothermal reaction of Zn(NO_3_)_2_·6H_2_O and urea. The ZnAl‐CO_3_ LDH released Zn^2+^ ions during the solvothermal reaction in methanol in the presence of the acidic ligand 2‐Hmim, leading to the formation of ZIF‐8. The SEM images showed that a well‐intergrown ZIF‐8 layer evenly covered the surface of the Al plate, and no conspicuous intercrystal defects were observed. The thickness of the ZIF‐8 layer was ≈5 µm, close to that of the ZnAl‐CO_3_ LDH buffer layer. DC polarization tests were performed with the ZIF‐8‐coated Al plate, ZnAl‐CO_3_ LDH‐coated Al plate and bare Al plate to quantitatively evaluate their anticorrosion performances. The results showed an *I*_corr_ of ≈1 × 10^−6^ A cm^−2^ for the bare Al plate and a largely decreased *I*_corr_ (≈1 × 10^−8^ A cm^−2^) for the ZIF‐8‐coated Al plate, comparable to those of the best quality inorganic anticorrosive coatings. The researchers also demonstrated that the anticorrosion performance of the ZIF‐8‐coated Al substrate was stable in a slightly acidic (pH = 6) 3.5 wt% NaCl aqueous solution for 5 days. In addition, the strong adhesion of the ZIF‐8 layer to the Al substrate was observed in a scratch test, which yielded no peeling off of the ZIF‐8 layer from the Al substrate after cross‐cutting.

### 4.7 Self‐Cleaning

Self‐cleaning coating materials have received considerable attention due to their commercial market in many fields, such as window glass, ceramic tiles, and textiles.^288^, ^289^ Superhydrophobic nanoparticles represent a type of classic material for artificial self‐cleaning coatings.^290^ Mimicking many biological objects (e.g., the lotus leaf, the rice leaf, cicada wings, and butterfly wings) in nature,^289^ artificial self‐cleaning surfaces coated with superhydrophobic nanoparticles strongly repel water drops with high static water contact angles (>150°) and a low contact angle hysteresis (≈0°); thus, water drops roll easily on the surfaces, and their rolling motion removes hydrophilic submicron particles, such as dirt, pollen, viruses, and bacteria.

**Figure 39:**
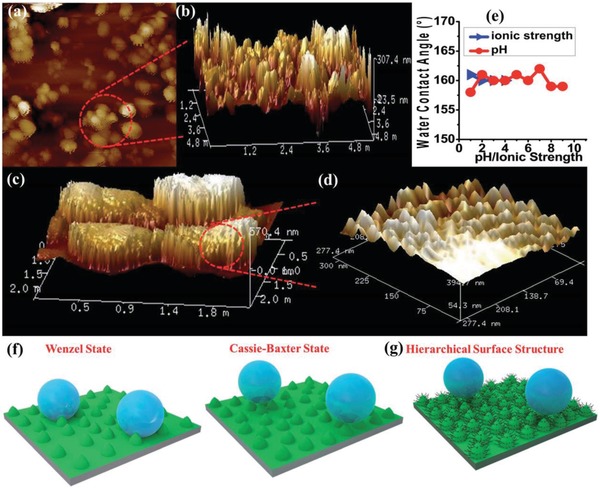
a–d) 2D and 3D AFM images of NMOF‐1 particles coated on glass. e) Contact angles measured with aqueous solutions at different pH or ionic strength. f) Schematic representations of two types of surface water repellence: the Wenzel state or the wetting state (left) and Cassie–Baxter state or the superhydrophobic state (right, rough texture with air‐pockets). g) The schematic hierarchical surface of NMOF‐1. Adapted with permission.^110^ Copyright 2016, Royal Society of Chemistry.

As pointed out in Section 3, a hydrophobic external surface of MOFs can be realized by constructing MOFs with carefully predesigned ligands or by the postsynthetic modification of MOFs via various methods. The MOFs with a superhydrophobic external surface are promising materials for self‐cleaning coatings. In 2016, Roy et al. reported a superhydrophobic and self‐cleaning MOF, [Zn(ope‐C_18_)(H_2_O)_2_] (NMOF‐1), which was synthesized from the solvothermal reaction of Zn^2+^ ions and the ligand H_2_ope‐C_18_, a dicarboxylic acid with long octadecyl alkyl chains.^110^ The crystal structure of NMOF‐1 was believed to consist of 1D coordination chains; in this configuration, Zn^2+^ ions are coordinated with four carboxylate O atoms from two ope‐C_18_^2−^ ligands and two coordinated water molecules, and each ope‐C_18_^2−^ ligand is bridging two Zn^2+^ ions. Gas adsorption studies showed type II or type III isotherms for N_2_ (77 K), CO_2_ (195 K), water (298 K), and benzene (298 K) with uptakes of ≈23, 35, 20, and 102 cm^3^ g^−1^, respectively, at ≈1 bar or a *P*/*P*_0_ close to 1, indicating the absence of micropores in NMOF‐1. SEM images revealed that the particles of NMOF‐1 were belt‐like with a length of 700–1000 nm and a width of 200–300 nm. Thanks to its nanoscale structure, NMOF‐1 was dispersible in ethanol. The water contact angle of a glass substrate coated with NMOF‐1 by its ethanolic dispersion was 160°–162°, suggesting its superhydrophobicity. The hydrophobicity should be inherited from the free ligand H_2_ope‐C_18_, which shows a water contact angle of 140°–147°. SEM and AFM measurements were carried out for the NMOF‐1‐coated glass surface and revealed that the aggregated NMOF‐1 was uniformly distributed on the surface as spherical particles, with a size of 10–30 µm and a height of 300–500 nm (**Figure** 39a,b). Moreover, the authors found that the NMOF‐1 particles possessed needle‐like nanoscale protrusions. Spikes 50–100 nm wide and 200–300 nm tall were observed on each particle, and the interspacing between these spikes was 10–50 nm (Figure 39c,d). The authors attributed the superhydrophobicity of the NMOF‐1‐coated surface to its hierarchical “hills and valleys” surface structure. A small contact angle hysteresis of 2° was observed for the surface. The self‐cleaning property of the surface was demonstrated by a recorded video, in which water droplets effectively removed preloaded dust from a slightly inclined glass coated with NMOF‐1. The results also proved that the NMOF‐1 coating was stable to acidic (pH: 1–6), weakly basic (pH: ≤ 9), and high‐ionic‐strength aqueous solutions, as suggested by the slight change in contact angles among the surfaces. A similar self‐cleaning property was also demonstrated for a superhydrophobic MOF (water contact angle: 161°), UiO‐66‐NH_2_‐shp, which was obtained by reacting phenylsilane with the hydroxyl groups in the Zr_6_O_4_(OH)_4_ clusters of UiO‐66‐NH_2_.^198^

## 5 Conclusions

The reviewed studies have demonstrated that hydrophobic MOFs with a wide range of pore sizes can be rationally designed by the deliberate choice of organic ligands and inorganic SBUs. Introducing hydrophobic groups onto the pore surface of MOFs reduces their affinity toward water; a substance that is ubiquitous. With this attribution, the hydrolytic stability of MOFs is normally improved. The reduction in water affinity and the enhancement in hydrolytic stability make hydrophobic MOFs promising advanced porous materials for many practical applications, especially for certain important separation systems in which water is unavoidable and negatively affects the performance of adsorbents, such as humid CO_2_ capture separation, alcohol/water separation, and the removal of environmental pollutants from air or water. In addition, some MOFs with a highly hydrophobic internal pore surface, such as ZIF‐8 and ZIF‐71, may serve as superior energy storage materials, which store energy by the forced intrusion of water into their hydrophobic pores at high pressures.

Alternatively, the hydrophobicity of some hydrophilic MOFs or some less‐hydrophobic MOFs can be enhanced by various postsynthetic methods. The postsynthetic hydrophobization can be carried out on the pore surface of the MOFs or only on the external surface of MOF particles with many kinds of hydrophobic substances via covalent bonding, coordination bonding, and even simply coating or encapsulating. The hydrophobization of MOFs is not always accompanied with a reduction in their porosity. On the other hand, the process may result in high water contact angles, an enhanced hydrolytic stability, a selective adsorption capacity, and/or an improved catalytic activity for some reactions. Some hydrophobized MOFs serve as high‐efficiency substrate‐selective catalysts or unique catalysts for certain reactions in which the hydrophilic nature of a catalyst is not favored. Some hydrophobic MOFs with high water contact angles repel water drops, and thus, the surfaces of specific substances (such as glass and metal substrates) coated with these materials can show the self‐cleaning and/or anticorrosion property. In these cases, the hydrophobic MOFs act more similar to a class of hydrophobic nanomaterials than hydrophobic porous materials. In the past, considerable attention has been paid to explore the potential usefulness of internal micropore in MOFs, while the significance of nanoscale MOF materials for certain applications, such as coating, catalysis, sensing and imaging, has been largely overlooked. As MOFs feature great structural diversity and designability, it can be foreseen that an increasing number of hydrophobic MOFs will be developed to meet the diverse application needs of the future, and the emergence of advanced multifunctional materials or devices based on both the hydrophobicity and porosity of MOFs can be expected.

## Conflict of Interest

The authors declare no conflict of interest.
